# A Systematic Review of Laser Photobiomodulation Dosimetry and Treatment Protocols in the Management of Medications-Related Osteonecrosis of the Jaws: A Rationalised Consensus for Future Randomised Controlled Clinical Trials

**DOI:** 10.3390/ph17081011

**Published:** 2024-07-31

**Authors:** Reem Hanna, Ioana Cristina Miron, Snehal Dalvi, Praveen Arany, René Jean Bensadoun, Stefano Benedicenti

**Affiliations:** 1 Department of Restorative Dental Sciences, UCL-Eastman Dental Institute, Medical Faculty, University College London, London WC1E 6DE, UK; 2 Department of Surgical Sciences and Integrated Diagnostics, University of Genoa, 16132 Genoa, Italy; dr.miron.ioana@gmail.com (I.C.M.); dr.dalvisnehal@gmail.com (S.D.); benedicenti@unige.it (S.B.); 3 Department of Periodontology, Swargiya Dadasaheb Kalmegh Smruti Dental College and Hospital, Nagpur 440001, India; 4 Department of Oral Biology ad Biomedical Engineering, University of Buffalo, Buffalo, NY 14215, USA; parany@buffalo.edu; 5 Centre de Haute Energie (CHE), 10, Bd Pasteur, 06000 Nice, France; renejean.bensadoun@che-nice.com

**Keywords:** bisphosphonates, consensus, guidelines, medication-related osteonecrosis of the jaws, MRONJ, photobiomodulation, preventive, therapeutic, oxidative stress, reactive oxygen species

## Abstract

Medication-related osteonecrosis of the jaw (MRONJ) is a debilitating adverse effect of bisphosphates, antiresorptive therapy or antiangiogenic agents that can potentially increase oxidative stress, leading to progressive osteonecrosis of the jaws. Despite the large number of published systematic reviews, there is a lack of potential MRONJ treatment protocols utilising photobiomodulation (PBM) as a single or adjunct therapy for preventive or therapeutic oncology or non-oncology cohort. Hence, this systematic review aimed to evaluate PBM laser efficacy and its dosimetry as a monotherapy or combined with the standard treatments for preventive or therapeutic approach in MRONJ management. The objectives of the review were as follows: (1) to establish PBM dosimetry and treatment protocols for preventive, therapeutic or combined approaches in MRONJ management; (2) to highlight and bridge the literature gaps in MRONJ diagnostics and management; and (3) to suggest rationalised consensus recommendations for future randomised controlled trials (RCTs) through the available evidence-based literature. This review was conducted according to the PRISMA guidelines, and the protocol was registered at PROSPERO under the ID CRD42021238175. A multi-database search was performed to identify articles of clinical studies published from their earliest records until 15 December 2023. The data were extracted from the relevant papers and analysed according to the outcomes selected in this review. In total, 12 out of 126 studies met the eligibility criteria. The striking inconsistent conclusions made by the various authors of the included studies were due to the heterogeneity in the methodology, diagnostic criteria and assessment tools, as well as in the reported outcomes, made it impossible to conduct a meta-analysis. PBM as a single or adjunct treatment modality is effective for MRONJ preventive or therapeutic management, but it was inconclusive to establish a standardised and replicable protocol due to the high risk of bias in a majority of the studies, but it was possible to extrapolate the PBM dosimetry of two studies that were close to the WALT recommended parameters. In conclusion, the authors established suggested rationalised consensus recommendations for future well-designed robust RCTs, utilising PBM as a monotherapy or an adjunct in preventive or therapeutic approach of MRONJ in an oncology and non-oncology cohort. This would pave the path for standardised PBM dosimetry and treatment protocols in MRONJ management.

## 1. Introduction

Medication-related osteonecrosis of the jaw (MRONJ) is a rare but a debilitating complication associated with antiresorptive (e.g., bisphosphonates (BPs) and denosumabs (DNBs)) [1,2], as well as angiogenesis-inhibitor (e.g., bevacizumab and sunitinib) medications [3,4], triggering a severe impact on patients’ quality of life (QoL). This is due to jawbone infection, chronic pain and compromised functionality, in which treatment can be very challenging [5]. MRONJ aetiopathogenesis remains unclear, despite the fact that a large number of patients suffer from this severe adverse event and great research effects have been invested [6].

### 1.1. Antiresorptive Agent

#### 1.1.1. Bisphosphonates

BPs and similarly acting bone antiresorptive agents, such as DNB, have become the principle, if not the sole, therapeutic agents for osteoporosis and primary metastatic skeletal malignancies, aiming to prevent bone fracture and minimise pain and metastatic spread [7]. BPs act as pyrophosphate analogues, which are a natural inhibitor of bone metabolism. Their mechanism of action has not yet been fully understood, but they are inhibitors of the osteoclast activity and inductors of their apoptosis, resulting in reduction of the bone remodelling process.

The main localised side effect of BP therapy is osteonecrosis of the jaw (ONJ) [8], but it can also be associated with systematic effects such as gastrointestinal disorders and atypical femoral fractures [9,10,11]. BPs are excreted in the kidney after being accumulated at the sies of active remodelling (both upper and lower jaws), due to their rapid deposition and long retention in the bones [12].

Zoledronic acid (ZA) is a third-generation BP and considered the most potent drug for clinical inhibition of bone resorption [13]. It accelerates osteogenesis of bone-marrow mesenchymal stem cells by attenuating oxidative stress (OS) via the SIRT3/SOD2 pathway and, hence, alleviates osteoporosis. Its half-shelf-life is 11.2 years when precipitated in the bones [12].

Oral BPs, on the other hand, are the most commonly prescribed in the treatment of osteoporosis and osteopenia. They are a medication of choice for bone diseases such as Paget’s disease, osteogenesis imperfecta, chronic recurrent multifocal osteomyelitis and for preventive heterotopic ossifications mostly of the spinal cord [14,15]. They are also indicated in the treatment of chronic kidney disease and transplantation, rheumatoid arthritis (RA) and spondylarthritis [16].

In terms of intravenous (IV) BPs, they are most potent in causing osteonecrosis [17] and are commonly utilised in various conditions associated with malignant diseases such as hypercalcemia caused by cancer and in bone metastases (secondaryism) [18], which release cytokines and growth factors to enhance osteoclasts activities. This can lead to bone resorption, favouring tumour growth. Intravenous BPs stimulate antitumour immune mechanisms, which inhibit growth, migration and secondary formation most commonly in breast and prostate cancers, multiple myeloma (MM) and aggressive chemotherapy (CT). Despite BPs initiate ONJ and have a great accumulative factor, they have positive effect on patient’s QoL [19]. It is noteworthy that ZA and DNB have similar potency in inducing osteonecrosis, but their times of accumulation in the body are shorter [20].

#### 1.1.2. Denosumabs (DNBs)

DNBs can induce ONJ (antiresorptive therapy (ART)-related osteonecrosis (ARONJ)). Patients who receive a high dose of ART are at slightly higher risk of developing ARONJ, and, hence, a multidisciplinary treatment approach in the prevention and therapeutic treatment is crucial [21].

DNBs are humanised monoclonal antibodies directed to a receptor activator of nuclear factor Kappa-B (RANK) ligand (modelling regulator), inhibiting osteoclasts and reducing bone resorption [22]. Their potency to induce ONJ alone has shown to be approximately similar to ZA potency [23], which is the most potent BP [24,25].

Additionally, the medication route of administration is an important factor in assessing the risk ONJ development. Oral BPs were shown to be safer than those administered IV [26,27], as their prevalence in developing ONJ (0–0.05%) is much lower than IV BPs and DNB prevalence (2–10%). It is noteworthy that the prevalence is increased with invasive dental surgical procedure and drug intake duration, as well as in patients with MM [28,29].

Another important factor in ONJ development is the duration of the ART. The literature states that after each year of therapy, the risk of MRONJ doubles [30]. They can be prescribed every day, once a week, once a month, once every three months or once every six months; hence, the incidence of ONJ increases with a higher dose and duration [31,32,33,34]. DNBs are administered subcutaneously and, unlike BPs, do not accumulate in the bone; therefore, their effect on bone remodelling is reversible and can last approximately for six months [20].

### 1.2. Antiangiogenic Drugs

These medications are tyrosine-kinase inhibitors: sunitinib/sorafenib, monoclonal antibodies targeting VEGF, preventing the formation of new blood vessels binding to various signalling molecules and inhibiting angiogenesis. Moreover, they cause an imbalance between bone deposition (osteoblastic activity) and resorption (osteoclastic activity) [5].

Bevacizumab is humanised monoclonal antibody binds selectively to a protein called vascular endothelial growth factor (VEGF) in the blood and lymph vessels [5]. It is utilised in the treatment of malignant diseases of the kidneys, gastrointestinal tract and lungs, as well as glioblastoma [35,36], whereas Sunitinib is used in the treatment of metastatic renal carcinoma and neuroendocrine tumours of the pancreas. It inhibits thyroxine kinase function [37], and if it is prescribed in conjunction with CT or BPs, it induces a high risk of ONJ development [38].

### 1.3. MRONJ Pathophysiology and Aetiopathogenesis

MRONJ aetiology and pathophysiology are complex and have been hypothesised its involvement with inflammation or infection, impaired bone remodelling and lack of angiogenesis due to the impact of bone-modifying agents, antiangiogenic drugs and some targeted medications. In the American Association of Oral and Maxillofacial Surgeons (AAOMS) Position Paper published in 2014, medical management was discussed as non-invasive therapy [27], and imaging investigation needs to be considered in MRONJ management, along with the clinical examination and staging assessment.

The precise mechanism of ONJ development remains unclear and could be multifactorial related to a combination of medication interactions, microbiological contamination of the affected area and local tissue trauma [39].

#### 1.3.1. Inhibition of Bone Resorption and Remodelling

ART inhibits osteoclast function and differentiation, leading to apoptosis and a reduction in the bone remodelling [40]. It also downregulates the activity of the osteoblasts, keratinocytes and the fibroblasts [41,42]. Of all the bones of the human skeleton, the jaws are the most susceptible bone for remodelling, and, hence, ONJ is triggered, whereas ART inhibits angiogenesis in the bone secondary to ONJ [31].

#### 1.3.2. Inflammation or Infection

Inflammation plays a significant role in ONJ development, and, also, OS is another contributing risk factor in this disease [43].

A pathohistological analysis of the necrotic bone revealed the presence of several types of bacteria, especially *Actinomyces* species in 70–100% of the cases [44]. Bacterial decontamination at the affected site with BPs has a synergistic effect in increasing the possibility of bacterial adhesion to hydroxyapatite of the jawbone [45,46], resulting in invasion of the microorganisms into the bone itself, due to a lack of angiogenesis [45].

#### 1.3.3. Mitochondrial Homeostasis and Oxidative Stress

A disturbance in the mitochondrial homeostasis due to pathological stress can lead to reactive oxygen species (ROS) production and energetic insufficiency [43]. On this note, OS was detected in patients with MRONJ, where the GSSG/GSH ratio was significantly reduced; hence, they are a significant biomarker in predicting ONJ development [47].

One of MRONJ pathogenesis theories is that drugs affect the fibroblasts directly, producing toxicity of the oral mucosa. Exposed bone and impaired oral mucosal healing play an important role in ONJ development [48]. Also, an increase in ONJ severity was observed in patients with ART together with other immunosuppressants such as corticosteroids or methotrexate [27].

### 1.4. Epidemiology of MRONJ Development

The incidence of MRONJ is multifactorial, and the factors are listed below.

#### 1.4.1. Local and Anatomical

Invasive dental procedures such as dentoalveolar surgery can increase the risk of MRONJ up to seven-fold [3], at a prevalence between 60 and 65% [47,49]. Also, dental diseases that patients have already overcome, such as periodontitis, periimplantitis, various inflammatory conditions of the jaw and poor oral hygiene, are among the additional risk factors contributing to ONJ development [49,50,51].

Mandible is the common site for ONJ (73%), whereas the incidence in the maxilla is about 22.5%, and it is very rare in both jaws simultaneously (4.5%) [27]. Interestingly, ONJ develops at sites of thinnest oral mucosa, such as the lingual aspect of the mandible, and at various exostoses sites [27,49]. On this note, patients who wear dental prostheses are subjected to a double-fold risk of developing ONJ [50].

#### 1.4.2. Systemic

The basic diseases that are fundamental in increasing the risk of ONJ development are as follows [52]: CT (40%), corticosteroid intake (25%) and diabetes mellitus (DM) (10%) [53,54]. Also, there are other diseases contributing to ONJ development, such as anaemia, systemic lupus erythematous, hypothyroidism, renal failure, RA, hypertension and smoking [55,56].

#### 1.4.3. Genetic

The risk of developing osteonecrosis is also associated with gene predisposition. There is an association between farnesyl diphosphate synthase gene (FDPS), encoding a key enzyme of the mevalonate pathway and ONJ development; hence, rs2297480 (a SNP region on the FDPS gene) is tested as a predicted biomarker [57].

### 1.5. Diagnostic Criteria

The AAOMS staging for MRONJ was published in 2014 [27]. Then, this was updated, and a Position Paper by AAOMS was published in 2022 [58]. Table 1 illustrates the details of the MRONJ staging.

### 1.6. MRONJ Clinical and Radiographical Presentations

A persistent exposure of the alveolar bone for >8 weeks in patients with a long-standing use of any bone modifying agent in the absence of head and neck radiation [1,2,58] is considered a diagnostic prediction of MRONJ [58]. Also, radiographic investigations are measured as good tools for MRONJ diagnosis and outcome evaluation [58].

### 1.7. MRONJ Management—Current Scientific Literature

Surgical and non-surgical approaches have been utilised in MRONJ management. However, each of them has its limitations [59]. This has led researchers, in recent years, to explore non-invasive treatment modalities; photobiomodulation therapy (PBM) has emerged to overcome the challenges, due to its analgesic [60,61], immunomodulatory, anti-inflammatory [62] and regenerative effects in wound [63,64] and bone repair [65,66]. Additionally, antimicrobial photodynamic therapy (aPDT) is considered an adjunct in MRONJ management [67].

These treatment modalities have been explored by the scientific literature, and, hence, we outlined these treatment strategies below.

#### 1.7.1. Medical Treatments

The updated AAOMS Position Paper published by Ruggiero et al. (2022) [58] stated that conservative (non-surgical) treatments, consisting of antibiotic therapy and antimicrobial mouth rinses, are considered the gold standard in MRONJ management, but the complete healing of lesions is not considered mandatory. However, stable lesion condition or MRONJ downstaging, according to the AAOMS, is considered the goal of conservative treatments [58,68,69].

It is noteworthy that the conservative approach can only lead to lesion resolution at an early stage of MRONJ and in a limited number of cases, according to the current AAOMS [58]. Hence, this treatment approach is poorly effective in more advanced stages of MRONJ.

#### 1.7.2. Invasive Treatment Modalities

The standard surgical approach (surgical drill)—either a minimally invasive (debridement) or extensive-resection approach—has been investigated.

Sequestrectomy is a minimally invasive surgical approach to removing necrotic bone sequestration, involving the margins of the adjacent healthy bone in order to generate bleeding to enhance vascularisation [70]. An extensive invasive surgical approach involving the removal of necrotic bone and its surrounding area (marginal mandibulectomy, or partial maxillectomy) is recommended for MRONJ advanced stages II and III [71].

Another surgical approach is using ultrasonic electric surgery, which is less invasive, offering a minimal trauma to the surrounding healthy bone tissue when the necrotic bone is removed. This approach maintains the continuity of the vital bone, which is beneficial for successful ONJ outcomes [72].

#### 1.7.3. Hormonal Therapy

Recombinant human parathyroid hormone (rhPTH, teriparatide) has been utilised as an adjunctive therapy in MRONJ management [73]. It assists in the healing process of MRONJ lesions by accelerating the resorption of the necrotic alveolar bone, reducing inflammation, enhancing neoformation of the alveolar bone and prompting epithelium regeneration [73].

An in vivo animal study was conducted by Jung et al. (2021) that demonstrated the potential effects of preoperative rhPTH as a preventive measure of ONJ development after dental surgical procedures in patients with a long-term history of BP intake or those at a high risk [74].

#### 1.7.4. Autologous Hemoderivatives

The autologous preparations are formulated from the patient’s blood and known as autologous platelet concentrates (APCs), such as platelet-rich plasma (PRP), platelet-rich fibrin (PRF) and concentrated growth factor (CGF), leading to a release of multiple growth factors to accelerate wound healing and tissue repair [75,76,77,78].

Currently, APCs are utilised in several clinical dental applications, due to their regenerative properties for hard and soft tissues [79,80,81], by stimulating the target cells to synthesise various growth factors such as transforming growth factor-β1 (TGF-β1), platelets derived growth factor-BB (PDGF-BB), VEGF-A and insulin growth factor-I (IGF-I). These growth factors are essential in cell proliferation and differentiation, chemotaxis, extracellular matrix production and tissue healing [82,83] because they promote leukocytes, increase collagen production, generate anti-inflammatory agents and enhance osteogenesis [84].

Several clinical trials showed the effectiveness of PRP, PRF and CGF [85,86] in reducing pain and post-operative infection [87,88], ultimately enhancing QoL in patients with MRONJ.

#### 1.7.5. Antimicrobial Photodynamic Therapy (aPDT)

aPDT can be effective when infection and/or suppuration are present [27]. It relies on activation of a photosensitiser by its compatible wavelength, resulting in production of a singlet oxygen and ROS, which ultimately lead to cell death [27].

a-PDT can be a promising preventive treatment in reducing the risk of ONJ development in non-oncologic osteoporotic patients, treated with non-intravenous ART or underwent dentoalveolar surgery. The clinical guidelines that were set up by Yarom et al. in 2019 [89] were designed to evaluate MRONJ outcome when aPDT was employed.

Due to the complex oral microbial environment, clinicians adopt more advanced therapies to tackle the multi-organism of MRONJ-associated biofilm. In this context, aPDT can be considered as an effective approach against many Gram-positive (Gram^+ve^) and Gram-negative (Gram^−ve^) bacterial pathogens, as well as parasites, fungi and viruses [90]. Care must be taken to differentiate aPDT from PBM as the primary goal of the former treatment is to debride or destroy its target, unlike PBM is non-destructive therapy that evokes stimulatory or inhibitory biological responses for tissue repair.

#### 1.7.6. PBM Therapy

There is a rapid expansion of the scientific research focusing on the potential of PBM in MRONJ management. Previous studies have shown that PBM can regulate critical cellular pathways and energetic cellular metabolism mediated by adenosine triphosphate (ATP), calcium (Ca^+2^) or ROS [91].

Several studies have indicated that PBM is a potential biophysical non-invasive treatment modality, contributing to wound healing by establishing homeostasis, reducing pain and inflammation and enhancing collagen accumulation and angiogenesis [92,93].

The infrared (IR) and near-infrared (NIR) laser irradiations can enhance bone repair and regeneration via two consecutive phases: cellular/intra-cellular and tissue modulating cascades, which are inter-dependent processes [94]. These two proposed phases are as follows [15]: (a) direct effects by stimulating the osteoblast proliferation, inhibiting the osteoclast activities, increasing the proliferation and differentiation of the fibroblast cells, upregulating the bone growth factors and modulating the cytokines and osteogenesis factors; and (b) indirect effects by modulating and enhancing bone formation and creating a friendly environment as a scaffold to facilitate bone regeneration and formation. This involves promoting cellular/tissue ion exchange, enhancing bone mineralisation and increasing NO, resulting in an increase in the vascularity and an improvement in the lymphatic circulation. Ultimately, it optimises bone healing and regeneration [66].

### 1.8. Rationale of Conducting the Present Systematic Review

The most profound effect of MRONJ is its negative impact on patients’ QoL. Thus, the challenge of the medical practitioner in treating these patients is undoubtedly to select the most appropriate medical protocols for maximising positive clinical outcomes for patients.

Currently, there is a lack of consensus regarding the most appropriate treatment strategy for MRONJ. This partially is due to the heterogeneity of MRONJ staging and available treatments; indeed, a majority of the proposed protocols are surgical (conservative or aggressive approach).

Despite the fact that MRONJ optimal treatment concept remains debatable, several adjunct therapies have been introduced. Among these adjunctive measures, PBM emerged as a promising alternative treatment due to its ability to modulate metabolic, biochemical and photophysical processes; promote analgesia and tissue repair; and modulate inflammatory cascades [6,7,8], but there is a lack of consensus in dosimetry and treatment protocols. Therefore, the present study aimed to evaluate the efficacy and dosimetry of laser-PBM as a monotherapy or combined therapy with the standard treatment approaches and aPDT (preventive or therapeutic) in MRONJ management. The objectives of the present review are as follows: (1) to highlight and bridge the literature gaps in the diagnosis and management of MRONJ, (2) to establish PBM dosimetry and treatment protocols for both preventive and therapeutic approaches in MRONJ management and (3) to offer consensus-based guidelines and recommendations for future randomised controlled clinical trials (RCTs).

## 2. Materials and Methods

### 2.1. Protocol and PROSPERO Registration

This systematic review was conducted according to Preferred Reporting Items for Systematic Reviews and Meta-Analyses (PRISMA) guidelines and Statement and Cochrane Collaboration recommendations (Supplementary File S1). The protocol was registered at PROSPERO, under the ID CRD42021238175. The review was conducted up to the period of 15 December 2023.

### 2.2. Focused Research Questions

(1) Can PBM as a monotherapy or adjunct to other standard treatment modalities provide superior positive effects by enhancing the healing process and reducing the recurrence rate of MRONJ disease?

(2) Do various PBM wavelengths have different effects on the healing mechanism in patients at a high risk of ONJ?

(3) Does a PBM preventive approach in MRONJ management improve patients’ QoL and prevent complications compared to therapeutic treatment modality?

### 2.3. Patient, Interventional, Comparative and Outcome (PICO)

**P:** Subjects ≥ 18 years old who are on BPs or oncology medications and developed MRONJ with different staging, as a result of oral intervention that affected the bone integrity and was diagnosed according to AAOMS [27,58] or any classification that fulfils the eligibility criteria.

**I:** PBM as a monotherapy or adjunct with other therapies for preventive or therapeutic or combined approach in MRONJ management.

**C:** Monotherapy or a combination of any of the following therapies: medical approach (Antibiotic and antifungal therapy); autologous hemoderivatives, (platelet-rich plasma (PRF) or leucocyte-PRF (L-PRF)); recombinant human bone morphogenetic proteins (BMPs); surgical approach—standard surgical debridement, sequestrectomy and surgical Er:YAG; piezo-surgery; ozone; and aPDT.

**O:** Clinical evaluation (soft- and hard-tissue healing); radiographical examination; histological analysis or microbiological assessment, as indicated.

### 2.4. Search Strategy

The search strategy included only terms related to or describing the study domain and intervention, which were conducted by two review authors (R.H. and I.C.M.) independently, and the studies were also screened by these reviewers independently, and a matrix of relevant data was produced. Inter-reviewer reliability was assessed using Kappa (κ) statistics, for which a minimum value of 0.8 was considered as acceptable [95]. In the case of any inconsistencies, a third review author (S.D.) was consulted to reach consensus. The following databases, using the relevant keywords and Medical Subjective Headings (MeSH) Terms were systematically searched: MEDLINE (NCBI PubMed and PMC), EMBASE, CINAHL, ClinicalTrials.gov, the Cochrane Library database, ProQuest, Scopus, Trial Registry for RCTs, Cochrane Central Register of Controlled Trials (CCRCT), ScienceDirect and Google Scholar, comparing PBM, as a monotherapy or adjunct therapy to placebo/PBM shame or other standard-care intervention. Additionally, the following journals were hand-searched: *Photomedicine and Laser Surgery*; *Journal of Oncology*; *Journal of Biophotonics*; *Oral Oncology*; *Journal of Osteoporosis*; *Journal of Dental Research*; *Lasers in Medical Sciences*; *Photodiagnosis and Photodynamic Therapy*; *Journal of Photochemistry and Photobiology*; *Craniomandibular Disorders*; *Laser Therapy*; *Oral Surg Oral Med Oral Pathol Oral Radiol Endod*; *Med Oral Patol Oral Cir Bucal*, *Lasers in Medical Science*; *Journal of Photochemistry and Photobiology B: Biology*; *British Journal of Oral and Maxillofacial Surgery*; *Frontiers in Oncology*; *International Journal of Oral and Maxillofacial Surgery*; *Journal of Oral Maxillofacial Surgery*; *Journal of Oral Pathology and Medicine; Journal of Clinical Oncology*; Journal of *Cranio-Maxillofacial Surgery*; *Oral Diseases*; *Oral Oncology*; *Oral Surgery, Oral Medicine, Oral Pathology, and Oral Radiology*; *J Clin Exp Dent*; and *Journal of Applied Oral Science*. The electronic search was meticulously explored up to 15 December 2023.

The search strategy included only terms related to or described the study domain and intervention. The terms were combined with the Cochrane MEDLINE filters for controlled trials of interventions.

MEDLINE (NCBI PubMed and PMC), the Cochrane Central Register of Controlled Trials (CENTRAL) (CCRCT), Scopus, ScienceDirect, Google Scholar, EMBASE and EBSCO were scanned for an investigation into the effectiveness of PBM therapy, as monotherapy or combined therapy, as a preventive or therapeutic approach, compared to the conventional methods and aPDT in the management of MRONJ.

Additionally, a hand (manual) search of references of the retained papers was undertaken to identify any further studies that the electronic search did not retrieve.

The search strategy included only terms related to or described the study domain and intervention. The terms were combined with the Cochrane MEDLINE filters for non-controlled trials of interventions.

### 2.5. Relevant Free Keywords and MeSH Terms

The resources Medical Subject Headings (MeSH), Health Sciences Descriptors (DeCS) and Embase Subject Headings were used to select the search descriptors as well relevant free keywords. The Boolean operators “**AND**” and “**OR**” were used to improve the search strategy through various combinations.

The following terms were searched in combination: “Photobiomodulation therapy” **OR** “phototherapy” **OR** “LLLT” **OR** “low-level laser therapy” **OR** “photochemotherapy” **AND** “surgical debridement” **OR** “photodynamic therapy” **OR** “medical therapy” **OR** “bone morphogenic protein therapy” **OR** “recombinant human bone morphogenetic proteins (BMPs)” **OR** “platelet-rich plasma (PRF) **OR** “leucocyte-PRF” **OR** “ozone” **AND** “Medications-related osteonecrosis of jaws” **OR** “MRONJ” **OR** “bisphosphate-induced bone necrosis of jaws” **OR** “BRONJ” **OR** “Antiresorptive-induced osteonecrosis of jaws” **AND** “Randomised controlled clinical trials” **OR** “case series” **OR** “non-randomised controlled clinical trials” **OR** “prospective randomised” **OR** “quasi-randomised” **OR** “non-randomised controlled clinical trials (CCTs)” **OR** “prospective clinical studies” **OR** “case series of more than 20 patients whereby ≥10 in each arm” **OR** “long-term follow-up (>3 months)” **OR** “retrospective clinical trials including RCTs” **OR** “cohort studies”.

Each of the below MeSH Terms used to find the relevant literature from the search engines in Section 2.4: Photobiomodulation (MeSH Major Topic) **OR** Low-level laser therapy (MeSH Major Topic) **OR** LLLT (MeSH Major Topic) **OR** Osteonecrosis of Jaw (MeSH Major Topic) **OR** MRONJ (MeSH Major Topic) **OR** ONJ (Mesh) **OR** BRONJ (MeSH Major Topic) **OR** aPDT (Mesh) **OR** PRF (Mesh) **OR** Ozone (Mesh) **OR** Bisphosphonates (Mesh) **OR** Medical therapy (Mesh) **OR** Surgical debridement (Mesh) **OR** Leucocyte-PRF (Mesh), **OR** PRF (Mesh).

### 2.6. Eligibility Criteria

**Inclusion Criteria**

1. Subjects ≥ 18 years old who were treated with bisphosphonate or oncology medications and developed ONJ by various degrees of MRONJ according to AAOMS, 2009, 2014 and 2022 [27,58], or any classification, as a result of oral intervention, that affected the bone integrity.
2. Subjects who did not receive radiotherapy in the craniofacial region and for whom the lesion has not healed during the 8 weeks following its identification by healthcare professional, according to the AAOMS.
3. Subjects who underwent various oral interventional procedures and subsequently developed ONJ.
4. Subjects who were on bisphosphates or any oncology medications regardless of dose, route of administration and treatment duration.
5. Studies utilised PBM as a monotherapy or combined with any of the following treatments and compared to any of them: medical approach (antibiotic and antifungal therapy); autologous hemoderivatives—PRF or L-PRF; BMPs; surgical approach—standard surgical debridement, sequestrectomy, surgical Er:YAG and piezo-surgery; ozone; and aPDT.
6. Studies utilised PBM wavelengths within the optical window (600–1100 nm).
7. Studies reported completer or incomplete PBM dosimetry and parameters.
8. Studies with a mean follow-up period of ≥3 months.
9. Studies treated any size of bone lesion.
10. Prospective randomised, quasi-randomised, non-randomised controlled clinical trials (CCTs), prospective clinical studies, case series of ≥20 patients or retrospective clinical trials, including RCTs and cohort studies.
11. Studies included ≥10 subjects in each interventional arm.
12. Studies in the English language.
13. The period of the search was up to 15 December 2023.

**Exclusion Criteria**

1. In vitro and in vivo animal studies.
2. Case reports or short communications.
3. Letter to the editor or any type of literature.
4. Case series studies of <20 patients.
5. Studies utilised < 10 subjects per interventional arm.
6. Studies used PBM wavelengths outside the optical window.
7. Studies utilised aPDT as a primary treatment or any other treatment modalities apart from PBM.
8. Subjects who had radiotherapy and developed osteoradionecrosis of the jaws.
9. Studies utilised home-based devices approach as part of the treatment protocols.
10. Studies utilised pharmacotherapy or any conventional treatment as a primary outcome.
11. Subjects with active malignant tumours.
12. Studies utilised homeopathic therapy as a comparative therapy.
13. Studies with the mean follow-up <3 months.

### 2.7. Review Outcome and Assessment Measures

The authors of this review employed specific criteria to evaluate the primary endpoint to allow them to extrapolate the optimal outcomes and their assessment tools for future RCTs in terms of preventive or therapeutic approach whether the cohort was oncology or non-oncology.

#### 2.7.1. Primary Endpoints

- Mucosal healing.

We employed the criteria of [89] to evaluate the mucosal healing based on “resolved”, “improving”, “stable” and “progressive”, taking into account symptoms, mucosal coverage and radiographic interpretation.

#### 2.7.2. Secondary Endpoint

- Healing time;
- QoL;
- Recurrence rate;
- Rate of complications and side effects of the intervention.

The breakdown of the secondary endpoints for PBM as preventive or therapeutic approach in MRONJ management is as follows:

1. Preventive Approach:
  - QoL;
  - Time-to-event;
  - Rate of complications and side effects of the intervention.
2. Therapeutic Approach:
  - QoL;
  - Recurrence;
  - Rate of complications and side effects of the intervention.

### 2.8. Data Extraction

A detailed electronic and hand search using our search strategy were performed. Studies obtained through duplicate searches were eliminated and the title and abstract of probable studies fulfilling the eligibility criteria were included in the review. Furthermore, studies that did not satisfy the eligibility criteria were removed from the cohort.

All the eligible studies were assessed for study quality and evidence synthesis. The data extracted from studies were noted in MS Excel. The data are categorised as study’s reference, study design, sample size, participants’ demographic chrematistics, baseline symptoms, MRONJ staging characteristics, intervention and comparator groups, risk factors, underlying comorbidity, primary diagnosis, MRONJ type, route of administration, doses, duration, bone lesion localisation, management description, duration and outcomes; and laser/LED dosimetry, number of sessions, duration of treatment, follow-up timepoints, statistical tests performed and outcomes. Two review authors (R.H. and I.C.M.) independently extracted the data. If any discrepancies or disagreement identified, they were resolved through a discussion until consensus was reached, and a third author (S.D.) would be consulted if needed. A similar search strategy and similar eligibility criteria were applied to obtain grey literature (unpublished data). In the case of missing information, authors were contacted and given 6 weeks to respond. If the information was not provided, the missing data were recorded as “not mentioned” (NM) in the text and in the tables.

### 2.9. Qualitative Analysis

As the included study cohort did not include any RCTs, as per the Cochrane collaboration’s guidelines, the evaluation of risk of bias (RoB) of observational and quasi-experimental studies was performed using the Risk of Bias in Non-Randomized Intervention Studies (ROBINS-I) tool [96,97]. The evaluated criteria were divided into pre-intervention, intervention and post-intervention categories. The questions pertaining to each domain were carefully and critically answered and the RoB was individually analysed for each study, and, consequently, every study received an overall score classified as low, moderate, serious, critical and no information [96,97]. In order to avoid any reviewer bias, two primary reviewers (R.H. and S.D.) carried out the ROBINS-I-tool assessments independently. For mitigation of any discrepancies in the findings in between the primary reviewers, discussions with a third author (I.C.M.) were conducted sequentially in order to obtain a final judgement [96,97]. A tabular, as well as graphical, representation was made to collect the data and present the results of the assessment.

### 2.10. Quantitative Analysis

At the time of project planning, a potential quantitative analysis was planned in order to assess the improvement in clinical signs of healing, if any, from the baseline visit to the final follow-up visit independently for studies utilising PBM monotherapy/combined therapy in comparison to other forms of treatment, as specified in our PICO for the management of ONJ. Accordingly, all relevant numerical data were to be extracted from the included studies, and the pooled data would be statistically analysed using the RevMan software (Version 5.4.1) [98]. A random-effects meta-analysis (MA) for continuous outcome measures would be utilised to assess heterogeneity. The plan was to group studies with similar a study design and other essential criteria and to conduct a distinctive MA on each cohort.

The use of pooled standardised mean differences (SMDs) with 95% confidence intervals (CIs) would be performed in order to calculate the treatment effects. A *p*-value of *p* < 0.05 was considered significant for the statistical analysis of pooled overall effect [99]. Furthermore, heterogeneity assessment would be carried out using forest and funnel plot analysis (I^2^ statistics for homogeneity that ranged from 0% to 100% with the following interpretation: 0% = no evidence of heterogeneity; 30–60% = moderate heterogeneity; and 75–100% = high heterogeneity) [99,100,101,102].

We would like to point out that an MA with significant findings could not be conducted since a high level of heterogeneity and failure to find uniformity amongst the eligible studies was noted by the authors. All the additional information obtained by the authors during data collection still was not useful enough to justify a meta-analysis. We highlight the discrepancies in our Results section.

## 3. Results

### 3.1. Study Selection

One thousand and ten study titles were obtained from a combined electronic and manual search. One hundred and twenty-six study titles were removed due to duplication (inter-reviewer agreement, κ = 0.92). Hence, a total of eight hundred and eight four study titles were included, from all the databases, in the preliminary screening process. Six hundred and seventy-seven articles were excluded by title and the remaining two hundred and seven records were further evaluated (κ = 0.90). One hundred and eight-one articles were excluded based on their abstracts, mainly due to an inappropriate study design (κ = 0.92). Thus, twenty-six articles were evaluated for eligibility criteria of this review, whereby twelve articles [103,104,105,106,107,108,109,110,111,112,113,114] were included in the review, and the remaining fourteen studies [115,116,117,118,119,120,121,122,123,124,125,126,127,128] were excluded due to the following reasons: <10 patients per group were in five studies [115,116,117,118,119]; PBM was not the primary intervention in four studies [120,121,122]; PBM wavelength outside the optical window in one study [123]; the mean follow-up <3 months in one study [124]; no follow-up timepoints in two studies [125,126,127]; and cohort underwent radiotherapy in one study [128] (κ = 0.94).

**Figure 1:**
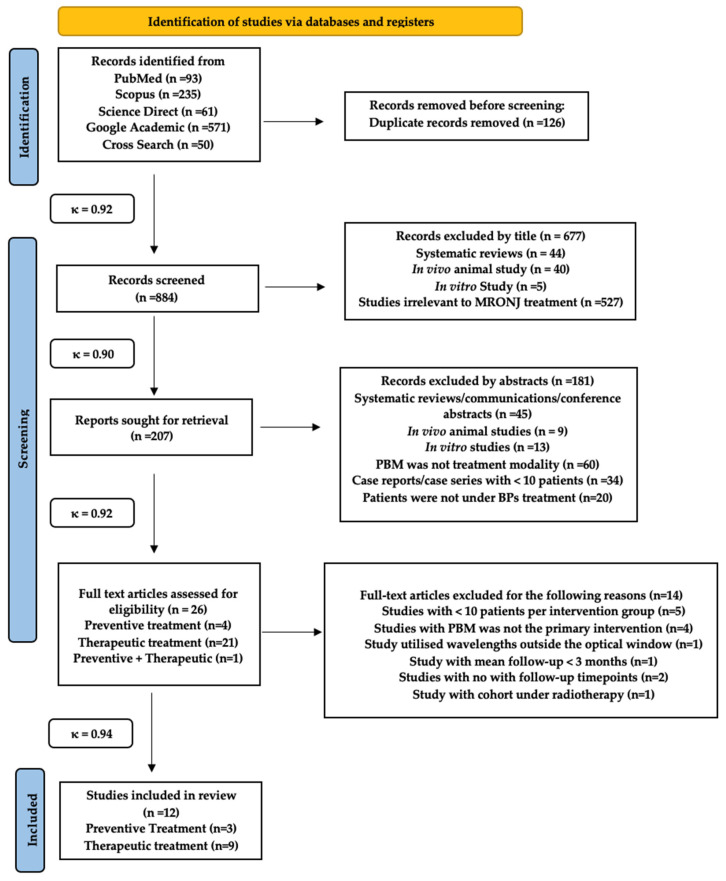
PRISMA diagram for the review search strategy.

Figure 1 PRISMA flow diagram for the search strategy in the present systematic review [129].

### 3.2. Study Characteristics

#### 3.2.1. Country of Origin

Italy was the predominant country of origin of the majority of the included papers, followed by Turkey and then France. The distribution of the studies was as follows: seven from Italy [103,106,110,111,112,113,114], four studies [104,105,108,109] from Turkey and one study [107] from France.

#### 3.2.2. Study Design

Nine studies were conducted using a retrospective study design [104,105,106,107,108,109,111,112,113], whereas a prospective study design was utilised in two studies [103,110], and the remaining one study [114] was a case series. None of the studies mentioned the eligibility criteria (inclusion and exclusion) in detail, but in six studies [103,105,108,109,111,113], the authors mentioned only one or two items for either inclusion or exclusion criteria, whereas the remaining six studies [104,106,107,110,114] did not specify. Moreover, none of the included studies documented their consort flowchart.

It is noteworthy that no RCT or quasi RCT studies were reported in the current scientific literature.

### 3.3. Participants Demographic Characteristics

#### 3.3.1. Age and Gender

Eleven out of twelve studies [103,104,105,106,107,108,109,110,112,113,114] mentioned the age of their recruited subjects; the mean age ranged between 55.4–72.6-year-old. The remaining study [111] failed to mention this. In terms of gender, all the studies mentioned this, and it appeared that both genders were employed in all the studies with unequal number. The percentage of total number of females treated in all the eligible studies was 26.07% versus (vs.) 73.81% in males. Table 2 shows all the data in detail.

#### 3.3.2. Sample Size and MRONJ Diagnostic Criteria

The distribution of the sample size in each study in relation to its interventional groups is as follows: 20 [103], 11 [104], 20 [105], 106 [106], 21 [107], 44 [108], 21 [113], 36 [110], 128 [111], 91 [112], 190 [113] and 217 [114]. Table 3 shows all the data details. None of the studies reported sample size calculation to determine the sample size of their studies. In terms of the employed MRONJ diagnostic criteria in the subject-recruitment process, a wide variation observed among the eligible studies, and the distribution was as follows: three studies [104,111,113] employed AAOMS 2009, two studies [105,112] utilised AAOMS 2007, three studies [106,107,109] used AAOMS 2014 and one study [103] employed Marx 2007 criteria. Meanwhile, the remaining three studies [108,110,114] failed to report.

#### 3.3.3. Number of the Lesions/Sites in Studies Employed Therapeutic Approach

One out of twelve studies [103] failed to mention the number of lesions and their sites. Three out of twelve employed preventive approach [108,110,114]. The remaining eight studies [104,105,106,107,109,111,112,113] reported a varied number of the lesions per site (maxilla or mandible or combined) and per study, and, hence, the total lesion per study varied from 15 to 190. Table 3 illustrates the number of the total lesions per study and their site distribution.

#### 3.3.4. Underlying Comorbidity and Other Medical Conditions

Six out of twelve studies [103,104,105,106,107,110,112] failed to mention the underlying comorbidity, whereas the remaining six mentioned the following comorbidities: hypertension [107], *DM* (did not specify the type) [107,108,109,111,113,114], arrythmia [107], renal failure [114] and vascular diseases [114]. In terms of other medical problems, ten out of twelve studies [103,106,107,108,109,110,111,112,113,114] mentioned the following conditions, and their distribution was as follows: corticosteroids in nine studies [103,106,107,108,109,110,111,112,113,114], CT in four studies [103,109,112,114] and hormonal and anticoagulant therapies in one study [114]. The remaining two studies did not specify [104,105].

Table 2 illustrates the details of all the above-mentioned data and their percentages.

#### 3.3.5. Type of Predisposing Trauma

In the studies that aimed for therapeutic MRONJ, dental extraction and minor oral surgery (MOS), respectively, were the predominant causes of MRONJ in eight studies [103,104,105,106,109,112], and the distribution of the rest of the causes was as follows: prosthetic denture irritation [103,104,107,109], dental implant [103,107,109] and spontaneous cause [107,112]. It is very clear that there is a lack of homogenous subjects in each group of each study. It is evident in the studies that aimed for a preventive approach to tackle MRONJ that there was no predisposing trauma [108,110,114].

All the above-mentioned data are illustrated in Table 2, along with their percentage values.

#### 3.3.6. Smoking Status

Seven out of twelve studies [103,107,108,109,111,113,114] mentioned the smoking status of their cohort, whereas the remaining studies failed to specify [104,105,106,110,112]. All the data, along with their percentage values, are illustrated in Table 2.

#### 3.3.7. Bisphosphonates Type, Duration and Route of Administration

Five out of twelve studies mentioned the use of BPs alone among their cohorts, without specifying the type [106,110,111,112,113], whereas the remaining seven studies mentioned the type of BPs/monoclonal antibodies used alone or in combination with another type of BPs or with monoclonal antibodies [103,104,105,107,108,109,114], and their distributions were as follows: ZA alone [103,104,105,107,108,109,114]; Alendronate (ALE) alone [103,108,114]; Pamidronate (PAM) [103,114]; Ibandronate [108]; Risedronate [114]; Clodronate [114]; monoclonal antibodies (DNB + Sunitinib) [107]; DNBs [106]; and BPs (type was not mentioned)+ DNB [106]. Ten out of twelve studies mentioned that the duration of BP intake by the cohort ranged between 2 and 164 months, and the distribution of the mean duration (month) was as follows: 42.95 ± 32.16 [103], 21.27 [104], 32.35 [105], 54.53 (61.9% cohort) and 38.1% cohort: NM [107]; 44.6 for IV route and 36.3 for oral route [108]; 64.76 ± 21.53 [109]; 28 [111]; 25 [112]; 26 ± 20 for oncology and 90 ± 40 for non-oncology [113]; and 17 for oncology and 53 for non-oncology [114], whereas the remaining two studies did not specify [106,110]. Nine out of twelve studies [103,104,105,106,107,108,109,114] mentioned the BPs’ route of administration, and the distribution was as follows: orally [103,106,107,108,109], IV [103,104,105,106,107,108,109] and intramuscularly (IM) [106]. The remaining four failed to mention. Only three out of twelve studies mentioned the BPs dose, as follows: ZA, 4 mg/monthly/IV (60%); ALE, 70 mg/weekly/orally (30%); ZOL + PAM, 90 mg/monthly/IV (20%) [103]; ZOL, 4 mg/28 d/IV (90% of cohort); ZOL, 3 mg/21 d/IV (10%) [104]; and ZA, 4 mg/month/IV (100%) [109].

#### 3.3.8. Bisphosphonate Treatment Break Prior to Dental Intervention (Drug Holiday)

The participants of eight out of twelve studies [104,105,106,109,110,111,112,114] stopped BPs, but with the number of cohorts who stopped BPs in each study varied, and their distribution was as follows: all the participants stopped the drug in two studies [104,109]; 19 out of 20 in one study [105]; 85 out of 106 in one study [106]; and 49 out of 217 in one study [114].

Two studies failed to mention the number of the participates who stopped BPs [111,112], whereas one study [110] mentioned 2 out of 36 subjects in which one of them stopped and the other one did not. Table 4 is a representation of BPs’ drug holiday. In two studies [103,108], all of the cohorts did not stop the drug, where four studies [111,112,113,114] had a cohort who stopped and others did not, and four studies had all of their cohort stop the drug. The remaining two studies [107,113] failed to mention the status of their cohort. In terms of the duration of the drug holiday, it varied, and the distribution was as follows: prior to surgery, 3 months [106] and 4.5 months [105,109]; 2 months pre- and post-surgery [114]; and until mucosal healing [104]. Three studies failed to specify [110,111,112,113].

### 3.4. Interventional Groups and Primary Disease Distribution

#### 3.4.1. Only Oncology Cohort

Three out of the twelve included studies [104,105,109] had only oncology cohort in which all received therapeutic approach in MRONJ management. The distribution of the primary diagnosis for this cohort was as follows: breast cancer [104,105,109], MM and PC [104,105,109], lung cancer [104,109], neuroendocrine malignancy [105] and kidney carcinoma [109]. Table 2 shows all the values including their percentages.

#### 3.4.2. Mixed Cohort: Oncology and Non-Oncology Cohort

A total of nine out of twelve studies [103,106,107,108,110,111,112,113,114] had oncology and non-oncology cohorts, and six of them offered a therapeutic approach [103,106,107,111,112,113], whereas the remaining three [108,110,114] received a preventive treatment approach. In the studies that undertook preventive approach, the distribution of the primary diagnosis of their cohort is as follows: breast cancer (BC) [103,108], MM [110,114], osteomyelitis [108,114], prostate cancer (PC) [13], nasopharyngeal cancer [(NPC) 108], bone metastasis [110,114], RA [114] and Paget’s disease [24]. Meanwhile, in the studies that undertook the therapeutic approach, the distribution of the primary diagnosis was as follows: BC [103], MM [103,111,112,113], osteomyelitis [103,111,112,113], PC [103], bone metastasis [111,112,113], RA [113] and solid tumour and osteo-metastatic disease [107], and only one study [107] failed to mention the primary diagnosis. Table 3 shows all the above details, including their values in percentage.

### 3.5. MRONJ Staging

Prior to the treatment, stage II and III of MRONJ staging were observed in the majority of the studies. There was a mixed of various MRONJ staging in each group of subjects for each study. There was a ack of homogenous subject recruitments: stage I [103,105,106,107,111,112,113], stage II [103,104,105,106,107,109,111,112,113] and stage III [103,104,106,107,109,111,112,113]. The remaining studies [108,110,114] were in the preventive approach of MRONJ. Table 2 illustrates the data.

### 3.6. Presenting Symptoms

Three out of twelve studies [108,110,114] employed preventive approach, and, hence, the initial symptoms were not applicable (NA), and one study failed to mention any symptoms, whereas the distribution of the symptoms in the remaining eight studies was as follows: bone exposure mentioned in eight studies [103,104,105,106,109,111,112,113], oroantral communication (OAC) in five studies [103,104,109,111,113], symptomatic/mobile teeth in two studies [104,106], pathological fracture in one study [103], extraoral fistula in one study [103], pus in three studies [103,106,112], paraesthesia in one study [112], inflamed mucosa in two studies [109,113], swelling in two studies [109,112], facial oedema in one study [103], halitosis in two studies [103,112], pain in two studies [103,104,112] and asymptomatic in one study [103]. Table 5 illustrates all the above-mentioned data.

### 3.7. Documentation of the PBM Parameters

Table 6 shows the results of the dosimetry parameters and treatment protocol.

#### 3.7.1. Utilised Wavelength (λ)

All the studies reported utilised wavelength. The distribution of PBM wavelengths was as follows: 660 nm [103,104,106,107], 904 nm [103], 810 nm [106], 808 nm [104,107] and 1064 nm [105,108,109,110,111,112,113,114].

#### 3.7.2. Power Output/Therapeutic Power Output (W, mW)/Emission Mode

A wide diversity in the employed power output was observed. It varied between 0.25 and 1.25 W. Seven out of twelve studies [108,109,110,111,112,113,114] employed 1.25 W in a pulsed emission mode (the same PBM laser protocol); one study [107] employed 1 W in CW; one study [104] utilised 0.5 W in CW emission mode; one study employed [105] 0.25 W in pulsed mode; and one study [106] used 0.5–1 W in CW. Only one study [103] failed to mention the power output but mentioned the emission mode (pulsed). Importantly, none of the studies mentioned whether the reported output power was therapeutic and whether a power meter was utilised. Table 6 illustrates these parameters.

#### 3.7.3. Power Output/Therapeutic Power Output (W, mW)/Emission Mode

A wide diversity in the employed power output observed. It varied between 0.25 and 1.25 W. Seven out of twelve studies [108,109,110,111,112,113,114] employed 1.25 W in a pulsed emission mode (those studies utilised the same PBM laser protocol); one study [107] employed 1 W in CW; one study [104] utilised 0.5 W in CW emission mode; one study employed [105] 0.25 W in pulsed mode; and one study [106] used 0.5–1 W in CW.

Only one study [103] failed to mention the power output in a pulsed-emission mode. Importantly, none of the studies mentioned whether the reported output power was therapeutic and measured with a power meter.

#### 3.7.4. Irradiation Time and Points

Nine out of twelve studies [104,105,107,108,109,110,111,112,113] mentioned the irradiation time, which has a wide range, from 3 to 60 s/spot; out of those nine, one study [104] reported 3 s/point (total 120 s) and eight studies [105,107,108,109,110,111,112,113] reported 60 s/point. The remaining three studies [103,106,114] failed to mention this parameter. Moreover, none of included studies mentioned the number of irradiated points.

#### 3.7.5. Reported Energy

Only two out of twelve studies [104,105] mentioned the energy parameter; of those two studies, one study reported 1.4 J [104], and the second study [105] reported 2.5 J. The remaining ten studies [103,106,107,108,109,110,111,112,113,114] failed to mention it.

#### 3.7.6. Energy Density (Dose, J/cm^2^)

Nine out of twelve studies [103,104,105,107,110,111,112,113,114] reported the fluence, and the distribution is as follows: 28.4 J/cm^2^ [103]; 5 J/cm^2^ [104]; 6.25 J/cm^2^ [105]; 21231 J/cm^2^ (theoretical according to the authors) [107]; 7 J/cm^2^ [110]; 14.37 J/cm^2^ [111]; 2.01 J/cm^2^ [112]; 14.37 J/cm^2^ [113]; and 7 J/cm^2^ [114]. The remaining three studies [106,108,109] failed to mention it.

#### 3.7.7. Irradiance (W/cm^2^)

Five out of twelve studies [110,111,112,113,114] reported irradiance (W/cm^2^) parameters, and their distributions as follows: 1262.5 [110], 268.81 [111], 268.57 [112], 268.81 [113] and 1562.5 [114]. These studies were conducted by the same research group and utilised the same protocol. The remaining seven studies failed to mention the irradiance parameters [103,104,105,106,107,108,109].

#### 3.7.8. Pulse Width (s, μs) and Frequency (Hz)

Nine out of twelve studies [103,104,105,109,110,114] employed pulsed emission mode, and all of them mentioned the frequency, but none of them mentioned the pulse width. The distribution of the frequency is as follows: 50 Hz [103], 15 Hz [104,109,110,111,112,113,114] and 10 Hz [105].

#### 3.7.9. Spot Size/Spot Area/Beam Diameter/Beam Profile

All the studies mentioned the spot size, but it varied in terms of the diameters. A spot size of 320 μm was utilised in eight studies [106,108,109,110,111,112,113,114], as they utilised the same protocol, whereas only one study for each of the following parameters was reported: 0.8 cm^2^ [103], 0.28 cm^2^ [104], 0.4 cm^2^ [105] and 600 μm [107]. None of the studies mentioned the utilised beam profile delivery system.

#### 3.7.10. Distance of Laser Tip-to-Target Tissue (Contact/Non-Contact)

Ten out of twelve studies [103,104,105,108,109,110,111,112,113,114] employed non-contact mode, and one of them failed to report the distance [103], whereas the other nine studies reported the distance and the distribution as follows: 0.5–1 cm [104], 4 cm [105], 1–2 mm [108,109] and 2 mm [110,111,112,113,114]. The remaining two studies [106,107] failed to mention it.

#### 3.7.11. Frequency and Treatment Duration

The treatment protocol varied. Table 6 represents the treatment frequency (number of sessions per week) and duration.

### 3.8. Interventional Groups Number and Associated Treatment Modalities

One study utilised PBM as a monotherapy with no comparative arms [103]. Five studies [104,108,109,110,114] had one group with different treatment modalities: PBM + med + CS [104,110,114], PBM + med + CS +PRF [108]; and PBM + med + CS + piezo + PRF [109].

Two studies [105,106] had two groups: PBM + med + SL vs. med + CS [6]; and PBM + med vs. med + CS [106].

Two studies [111,112] had four groups with different treatment modality protocols, and their distributions were as follows: G1: med, G2: PBM + med, G3: med + CS, G4: PBM + med + CS + SL (Er:YAG) [111]; G1: med, G2: PBM + med, G3: CS, G4: PBM + SL [112].

One study [113] had five groups of different treatment modalities protocol, and its distribution was as follows: G1, med; G2, PBM + med; G3, CS; G4, PBM + CS; and G5, SL. One study [107] had seven groups, and the distribution of the treatments was follows: G1, PBM + CS + SL + piezo+ PRP; G2, PBM + CS+ piezo + PRP; G3, PBM + CS+ SL + PRP; G4, PBM + CS+ PRP; G5, PBM + SL + piezo + PRP; G6, PBM + piezo + PRP; and G7, PBM + SL + PRP. The results of this subsection are illustrated in Table 3.

It is noteworthy that none of the included studies utilised aPDT as an adjunct therapy to PBM.

### 3.9. Medical and Antiseptic Treatment Regimens

Seven out of twelve studies [7,10,13,14,18,20,21] employed antibiotics and antiseptic (mouthwash) regimens pre- and post-operatively for 14 d; however, the antibiotic type, dose and route of administration varied. The remaining studies employed these regimen protocols either pre- or post-operatively.

Table 7 illustrates the medical and antiseptic mouthwash protocols that were employed in the eligible studies in terms of type, dose, frequency and duration of the antibiotics, as well the antiseptic mouth-rinse schedule. Additionally, Table 7 represents the missing data.

### 3.10. Outcome Assessment Tools

Table 8 shows outcome assessment measures that were employed in all the included studies, which varied in terms of mucosal healing, clinical examination and clinical photos and imaging (cone beam computer tomography (CBCT) and orthopantomogram (OPT)) were employed, whereas, in relation to pain intensity, visual analogue scale (VAS) was employed.

### 3.11. MRONJ Diagnostic Tools

Table 8 illustrates the tools in which MRONJ lesion was diagnosed. Six out of twelve studies [104,107,111,112,113,114] employed OPT and CBCT, whereas five studies [105,106,108,109,110] utilised only OPT, and the remaining study did not specify [103]. A histological examination was employed in three out of twelve studies [104,106,107], whereas one study utilised of CTX test [104].

### 3.12. Evaluation of MRONJ Outcomes

Table 9 represents the MRONJ outcomes (resolved/improves/stable/progressive/ recurrence [89]), including staging improvement after treatment.

### 3.13. Representation of MRONJ Staging Downscaled

Table 10 illustrates the results of the MRONJ staging downscaled after the treatments in the studies employed the therapeutic approach. There was a wide range in the percentage of downscaling MRONJ staging among various treatment protocols in the same study and among the included studies. Moreover, there was no information in relation to the initial staging of the cohort and for each interventional arm. Downscaled staging of MRONJ was not applicable in three studies [108,110,114] that employed preventive approach.

### 3.14. Declaration of Funding

Only one out of twelve studies [108] declared no funding, whereas the remaining eleven did not specified.

### 3.15. Assessment Clinical Parameters

Table 11 describes clinical parameters assessment used for the included studies.

With regard to pain and infection, statistically significant results were obtained in one study [103]. Meanwhile, in two studies [110,114], the statistical data were not specified. There was no relevant information in the remaining studies [104,105,106,107,108,109,111,112,113].

No information was available on the clinical parameters, paraesthesia, OAC, no clinical response and lesion recurrence amongst the eligible studies [103,104,105,106,107,108,109,110,111,112,113,114]. One study [103] reported no statistically significant difference for bone exposure, whereas no information on this parameter was available for the remaining eleven eligible studies [104,105,106,107,108,109,110,111,112,113,114].

A statistically significant difference in complete mucosal healing was observed in four out of twelve studies [106,107,109,110,114], whereas it was not statistically significant in two studies [105,108]. Five studies [106,107,109,110,114] did not specify the statistical information on this parameter, whereas one study [104] provided no relevant information at all. In terms of a complete lesion resolution, two studies [103,113] mentioned statistically significant improvement in results, and one study [114] did not specify the statistical data, whereas the remaining nine studies [104,105,106,107,108,109,110,111,112] provided no relevant information on this parameter.

### 3.16. Qualitative Assessment

**Figure 2:**
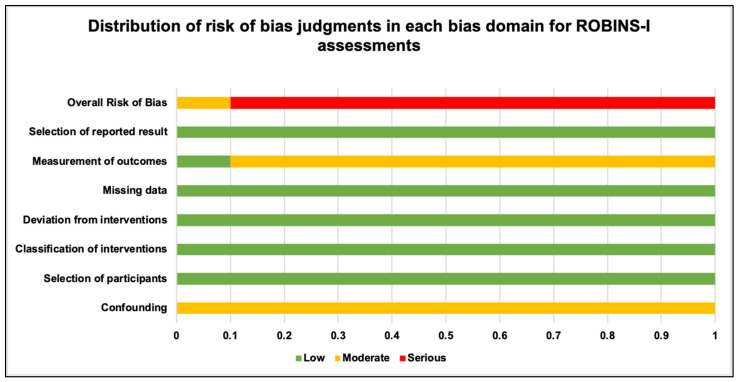
Graphical summary of distribution of risk-of-bias judgments in each bias domain for ROBINS-I assessment.

Each included study was assessed for its methodological quality using the ROBINS-I tool, which is specially designed for observational and quasi-experimental studies [96,97]. Figure 2 is a graphical summary of the scores, in a percentage format, that each of the included studies of this review received in the five domains of this tool, and an overall risk-of-bias score is also denoted for each study.

Table 12 is an overview of the results of the ROBINS-I assessment. The results of our qualitative analysis are based on the joint agreement of two independent reviewers (R.H. and S.D.) (κ = 0.90). A third reviewer of this project (I.C.M.) was contacted to discuss any disparities in the individual analysis of the above two reviewers [96,97]. The results were elaborated as shown in Table 12.

All included studies have received a “moderate score” for the confounding domain (100%). In terms of the domains based on selection of participants, classification of interventions, deviation from interventions, missing data and selection of reported results all included studies that received 100%, “low score”.

For the domain on measurement of outcomes, 10% of the included studies received a “low score”, and 90% of the studies received a “moderate score”. Overall, 90% studies reported a high risk of bias, and 10% studies reported a low risk of bias based on the guidance document provided with the tool [96,97].

### 3.17. Quantitative Assessment

In our Methodology section, we mentioned our intention to conduct a meta-analysis. However, owing to several notable discrepancies, as well as lack of numerical data in the included studies, a meta-analysis of the reported outcomes could not be carried out. Table 13 highlights all the key findings of our project analysis. Some noteworthy confounding features amongst the eligible studies are as follows: study design, study type, study protocol, laser parameters, risk-of-bias analysis, variations in outcome measures, lack of/disparities in numerical-data presentation, diverse follow-up durations, etc., in the individual eligible study results. Thus, owing to the large amount of clinical, statistical and methodological heterogeneity, relevant numerical data could not be procured from the included studies; hence, it was agreed by all review authors that a meta-analysis was not justifiable at this time. Furthermore, the authors believe that the confounding elements noted in this process could be utilised by researchers to imply in their research in order to conduct a potential MA on this subject in the future.

## 4. Discussion

MRONJ is a debilitating adverse effect of BPs, ART or antiangiogenic agents potentially can lead to progressive ONJ. Despite the large number of systematic reviews examined the potential preventive/therapeutic protocols for oncology and non-oncology cohorts, it remains a conflicting issue among the scientific community without agreed consensus.

Based on the hypothesis of PBM as a monotherapy or as an adjunct to several treatment protocols that can enhance the clinical or microbiological or immunological profile, a critical appraisal of the available scientific evidence was conducted. After meticulous scrutiny of the literature, twelve studies satisfied the eligibility criteria and were included in the present systematic review.

Due to the heterogeneity in the methodology, diagnostic criteria and assessment tools, as well as reported outcomes, it was impossible to conduct a meta-analysis of the included papers.

In order to gain an insight on the merits and inadequacies of the included studies, a comprehensive systematic investigation of the pertinent literature was performed and described below, aiming to establish a rationalised consensus and recommendations, if possible, for future well-designed, prospective, randomised clinical trials in the management of MRONJ, as such trials are lacking in the current scientific literature.

### 4.1. Description Analysis of Demographic Characteristics

The prevalence of ONJ in the recruited subjects of all the eligible studies who were treated with ART and BPs varied among genders, in which female was predominant (73.81%) compared to male (26.07%) (Table 14). This was observed in a recent systematic review and meta-analysis conducted by Diioguardi et al. (2023) [130].

In this context, it was evident that osteoporotic patients were mainly female largely involved with MRONJ, as well as subjects with BC among the eligible studies of the present systematic review.

Additionally, the mean age of the recruited subjects in this review was 67-years-old (52%), taking into account 8.33% of the total studies failed to specify. This is supported by a systematic review conducted by Gaudin et al. (2015) [131].

### 4.2. MRONJ Incidence, Associated Risk Factors and Affected Site

Taking the above-mentioned notes into consideration, it is noteworthy that oncology and non-oncology scenarios are likely to determine the differences in the proportion of osteonecrosis onset tendency for both genders who are undergoing MOS, knowing that ART for oncology increases the risk of ONJ development [132], including the drug route of administration and duration (Table 14). Hence, the authors of the present review indicate that the primary pathology can determine the incidence of MRONJ rather than the gender-dependent response. Further research is required.

In all the eligible studies, the percentage distribution of the contributed risks factors in increasing MRONJ incidence was as follows: 77% extraction/MOS, 75% corticosteroids, 50% DM, 44% denture wearer, and 22.22% absence of traumatic factors.

Depending on the drug type, dosage and the duration of treatment exposure, drug adverse reaction may rarely occur following oral administration of BPs or denosumab for osteoporosis, or antiangiogenic agent-targeted cancer treatment, or commonly occurs in more than 90% of MRONJ cases, receiving high doses of IV BPs or SC denosumab (120 mg every 4 weeks) for cancer treatments.

An IV route was the predominant route of drug administration (66.66%) among the cohort of the included studies in the present systematic review. Zoledronic acid (58.33%) was the prevalent drug of choice, and this in agreement with a recent meta-analysis conducted by Momesso et al. (2020) [133].

The introduction of denosumab in oncology and osteoporosis patients has generated a higher rate of spontaneous ONJ development than in BPs [134]. An increase in the proportion of cases of spontaneous MRONJ does not eliminate the risk factor of MOS induced ONJ in this population, and, hence, precautions should be followed, according to the American Society of Clinical Oncology (ASCO) clinical practice guidelines of July 2019 [89].

The patient’s immune response was negatively influenced by treatment duration, trigger factor, lesion location in the mandible and recurrence rate [135]. Hence, follow-up timepoint should be at least three years for this primary outcome.

MRONJ involves progressive destruction of bone in the mandible or maxilla, and its occurrence depends on the medication type, dosage and duration of exposure. Mandible is the most affected site of osteonecrosis lesion (66.23%) (Table 3), and this finding is in agreement with a meta-analysis conducted by Momesso et al. (2020) (64.5%) [133].

On a molecular level, BPs can induce ROS production, resulting in the inhibition of oral fibroblasts’ proliferation and migration [136]. Although BPs affect osteoclast function throughout the skeletal system, only the jawbones can suffer from MRONJ by which the mandible is two-fold more often affected than the maxilla [137,138]. This is due to a low vascularity in the mandible; hence, it is more susceptible for a higher infection rate [139].

Bone exposure (100%) was present in the cohort of all the nine studies [103,104,105,106,107,109,111,112,113] that employed the therapeutic approach of MRONJ. Local infection (55.55%) and pain (44.44%) were noted in all the nine studies. On this note, stage II of MRONJ was prevalent in all the nine studies that employed therapeutic approach (100%), and this finding is in agreement with a meta-analysis conducted by Momesso et al. (2020) (68.9%) [133], followed by stage III (88.88%) and stage I (77.77%).

A study conducted by Querrer et al. (2021) reported an increase in bone sequestra, cortical bone necrosis and less bone density observed in BP-related ONJ, whereas larger bone sequestra, more frequent periosteal reactions and mandibular canal enhancement were noted in denosumab-related ONJ [140]. In the present review, the cohort of only two out of twelve studies [106,107] were on denosumab. On this note, drug holiday of BPs, ART and antiangiogenic was implemented at least 3 months prior to treatment to all the cohort, but none of those two studies reported a correlation between type of drug and the risk of ONJ development.

### 4.3. Methodology Quality

#### 4.3.1. Evaluation of Study Design

Nine studies [104,105,106,107,108,109,111,112,113] in the present review were retrospective, whereas a prospective study design was utilised in two studies [103,110], and the remaining one study [114] was a case series. None of the studies mentioned the eligibility criteria (inclusion and exclusion) in details, but in six studies [103,105,108,109,111,113], only one or two items for either inclusion or exclusion criteria mentioned. Moreover, none of the included studies reported consort flowchart.

It is noteworthy, according to the review’s eligibility criteria, that there was no evidence of any RCT or quasi-RCT studies published in the scientific literature during the review timeframe (up to 15 December 2023). This was supported by several systematic reviews [141,142] employed different eligibility criteria, review research-focused questions and PICO.

Table 2, Table 3 and Table 4 illustrate discrepancy and inconsistency between/among the interventional arms of the included studies in terms of the number of recruited subjects and their gender, number of lesions and sites, comorbidity, primary disease, oncology and non-oncology cohort, BPs or ART duration, MRONJ grading, drug holiday and duration. Moreover, none of the studies employed sample size calculation to determine the number of recruited subjects in each interventional arm to fulfil the study endpoints and objectives. Identifying the correct sample size of any study is critical to determine the confidence level of the results. A large sample size increases the statistical power, leading to higher precision in study estimation and a smaller margin of error.

#### 4.3.2. Role of Diagnostic Criteria and Outcome Tools in MRONJ Prediction

There is a discrepancy among the current European and American Guidelines in the diagnostic criteria and assessment tools in MRONJ prediction. This inconsistency was evident among the included studies of the present systematic review. We comprehensively outlined the current evidence-based literature in the MRONJ prediction below.

- MRONJ Staging

Several modifications have been reported since the first AAOMS 2009 staging proposal, reflecting scientific progress. However, controversies persist when comparing the main available staging systems.

The AAOMS guidelines primarily staged MRONJ based on clinical presentation, while the SICMF–SIPMO guidelines [143] incorporated detailed clinical and radiological criteria. The American board has been hesitant to include radiological findings as a diagnostic or staging criterion due to inconsistencies in the clinical studies.

According to AAOMS classification 2022 [58] (Table 1), MRONJ is categorised into four stages. In contrast, the Italian Socies of Maxillofacial Surgery (SICMF)–Italian Societies of Oral Pathology and Medicine (SIPMO) 2020 recommendations outlined three stages based on the clinical and radiological findings, omitting “stage 0” that was proposed by the AAOMS counterpart. Stage 0 refers to patients who exhibit suggestive symptoms without obvious radiological or clinical evidence of MRONJ. This classification rationale aligns with orthopaedic practices, where “stage 0” encompasses patients at risk of developing avascular necrosis without evident disease findings [58,144]. Moreover, the rational of AAOMS adopting different perspective was based on the reported evidence, highlighting the advantages of early surgical treatment.

Both the Italian and American committees have acknowledged that a significant percentage of patients with “stage 0” MRONJ would progress to more severe stages. Based on this evidence, however, the SICMF–SIPMO board declined to include it in their proposed staging system [58,143]. Notably, the AAOMS guidelines published in 2022 [58] provided more clinical definition based on the presence of the following features: (1) current or previous treatment with antiresorptive therapy alone or in combination with immune modulators or antiangiogenic medications; (2) exposed bone or bone that can be probed through an intraoral or extraoral fistula in the maxillofacial region that has persisted for > eight weeks; and (3) no history of radiation therapy to the jaws or metastatic disease in the jaws.

Despite Italian expert board introduced radiological criterion based on computer tomography scans in their guidelines, certain controversies remain, particularly in relation to standardised use of radiological findings for staging purposes. However, all the included studies in the present review employed imaging investigations for diagnosis and outcomes assessments. It is important to highlight one out of twelve studies [103] in the present review included osteonecrosis lesions of <2.5 cm in size, whereas one study [106] included any lesion size without specification in their inclusion criteria. The remaining ten studies failed to mention.

Despite some initial controversies in the two sets of recommendations (AAOMS 2022 and SICMF-SIPMO 2020) were resolved, differences in relation to diagnosis and staging remain controversial [145].

In the present review, a wide variation was observed among the eligible studies in terms of employed diagnostic criteria, and this could be partly due to the time period in which six studies were conducted between 2007 and 2013 [103,105,111,112,113,114], whereas the remaining six studies were published in the period between 2014 and 2021 [104,106,107,108,109,110] in which only three utilised AAOMS 2014 criteria [104,106,107,109], two studies failed to report and one study utilised AAOMS 2009, despite the fact that it was published in 2014.

Only one study [103] that was published in 2010 employed Marx criteria [146], and none of the included studies employed SICMF–SIPMO 2020 criteria.

- Molecular Biomarkers

In terms of molecular biomarkers, extensive efforts have been invested to explore whether biomarkers can add value to MRONJ diagnostic criteria and endpoints assessment. Nevertheless, the scientific literature remains debatable on the validity of the association between predictive biomarkers and risk of MRONJ.

A systematic review conducted by Moraschini et al. (2019) [147] investigated the evidence of 22 biomarkers in the diagnosis, prediction and severity of MRONJ. The following eleven biomarkers showed positive evidence: CTX, bone alkaline phosphate (BAP), IL-17, neutrophil function, OS, N-telopeptide (NTX), metalloproteinase-9 (MMP-9), VEGF, c-reactive protein (CRP) and leukocyte count. Meanwhile, the remaining eleven biomarkers showed no evidence and were as follows: osteocalcin (OCN), parathyroid hormone (PTH), triiodothyronine (T3), thyroxine (T4), thyroid stimulating hormone (TSH), vitamin D 25-hydroxy, dihydropyrimidine dehydrogenase (DPD), procollagen type I N-terminal propeptide (P1NP), sclerostin, alkaline phosphate (ALP) and RANKL/OPG ratio. There is a little clinical evidence to support the validity of these markers; hence, any association between biomarkers and MRONJ should be interpretated with caution. This was supported by another systematic review conducted by Lorenzo-Pouso et al. (2019) [148], reporting no useful biomarkers are currently available to evaluate MRONJ onset, but it indicated a paradigm shift from bone turnover biomarkers to angiogenesis and endocrine markers, paves the path for future research. Another systematic review conducted by Prá et al. (2017) indicated that CTX is not a predictive tool in determining the onset risk of BRONJ in patients taking BPs [149].

Only one out of twelve studies [105] (Table 5) in the present systematic review utilised CTX as diagnostic and outcome assessment tools. Nevertheless, all the eligible studies employed clinical and imaging instrumentations, as diagnostic and treatment outcomes tools and this was supported by the scientific literature [150].

Interestingly, the availability of reliable salivary biomarkers for an early diagnosis of MRONJ could make a major contribution in prescribing appropriate management strategy, aiming to reduce morbidity.

Hypotaurine is an intermediate in the biosynthesis of taurine, which acts as an antioxidant in cellular defence against OS, and the detection of an increase in salivary levels may be an indicative tool for early MRONJ diagnosis [151], but the authors suggested further studies to further validate the biomarkers.

Began et al., in 2012 and 2013 [152,153], observed an increase in IL-1RA, IL1α, IL1β and IL6 in the saliva of patients with MRONJ compared to healthy individuals. Two studies conducted by Thumbigere-Math et al. (2013 and 2015) [154,155], showed elevated salivary levels of MMP-9 in patients with MRONJ; hence, they proposed this protein as a biomarker of this disease.

- Correlation between Duration of BPs Intake and MRONJ Prediction

The evidence suggests an association between the duration of BPs intake and risk of MRONJ [156] due to BPs’ half-life of 11.2 years, and all BPs connect severely to bone mineralised matrix. Hence, this cumulative mechanism of BPs molecules in the alveolar bone (rapid turnover) can be considered the pathogenetic cause of MRONJ involving the jaw bones [156]. On another hand, DB has a short half-life (26 d) and does not bind to the bone, but it is potent enough to induce ONJ, affecting osteoclast growth in the bone marrow [157].

In the present systematic review, the duration of BPs intake varied among oncology and non-oncology cohorts (Table 4) at an average of 66 months and 22 months respectively. None of the studies’ results indicated whether the duration of BPs or DB intake had an impact on the healing outcome of the osteonecrosis lesion.

Currently, there is no robust predictive tools identifying individuals who take BPs can be at a higher risk of developing MRONJ. Equally, there are no prognostic indicators That can predict outcomes. This was supported by the AAOMS 2022 [58]. The authors of the present systematic review encourage future studies to determine this link.

- Histological Analysis

Microbiological analysis of osteonecrosis lesions revealed the presence of species such as *Fusobacterium*, *Eikenella*, *Bacillus*, *Actinomyces*, *Staphylococcus* and *Streptococcus* [158,159,160]. The majority of the microbes in affected patients appears to be facultative anaerobes. These organisms are predisposed to survive in oxygen-depleted areas of necrotic bone associated with a lack of adequate blood supply, typically in MRONJ cases [161]. This diagnostic tool can indicate the nature of the microorganisms and the relevant effective systemic antibiotic, if the medical therapy was appropriate.

Taking into consideration all the above-mentioned data, currently, the most reliable tools for the onset of MRONJ risk and outcomes evaluation are the clinical examination (clinical photographs/clinical symptoms) (Table 5) and patient’s thorough medical history supplemented with imaging investigations.

The authors of the present review encourage more studies evaluating further the validity of the proinflammatory salivary and bone molecular biomarkers, as well as the link between duration of BPs and DB intakes and risk of ONJ.

Based on the above best critically appraised evidence-based scientific literature, including the present systematic review, the authors proposed suggested recommendations for the diagnostic and outcome assessment tools that can be employed in future extensive RCTs.

### 4.4. Evaluation of Holiday Protocols in MRONJ Reduction

A drug holiday is a temporary discontinuation of a drug and has been suggested among risk reduction strategies, but up to date, there is a lack of evidence-based science and practice among the scientific community and literature and hence drug holiday remains controversial and this is due to a limited number of eligible patients, and a great variation among them, as well as the difficulty to obtain data of ART holiday cohort [162].

Interestingly, employing a drug-holiday protocol prior to any invasive procedure remains a controversial issue [163]. Ottesen et al. (2020) [164] suggested that high-dose of AR drug holiday at the time of dental extraction or any MOS intervention should be considered to prevent MRONJ development in oncology cohort AAOMS position paper on MRONJ stated that two-month drug holiday before and after dental surgery in patients receiving oral BPs may be prudent. An international ONJ task force recommended that treatment should be withheld after invasive dental surgery in patients receiving high-dose BPs or DB [165,166].

The AAOMS recommendations 2014 and 2022 acknowledged the evidence of BPs drug holiday is scary; hence, the concept remains controversial.

Table 4 shows the variation in the drug-holiday protocols employed in the eligible studies of the present review. Only one study [114] adhered the AAOMS recommendations. Four studies [111,112,113,114] had their cohort stopped others, whereas four studies had all their cohort stopped the drug. Due to the inconsistency and diversity in the drug holiday protocols in the present review, as well as its controversial in literature, the authors of the present review would not be able to offer suggested recommendations for future RCTs, but they truly believe a consensus between physicians, oncologists and dentists would be the appropriate approach, weighting the benefits versus the risks.

### 4.5. Therapeutic Protocol Strategy

Despite several therapeutic protocols have been proposed in the literature, there is no common rationalised consensus. Hence, the authors of this review scrutinised the included studies, as well as the scientific literature in this section, to establish proposed suggested recommendations of therapeutic PBM as a single or as an adjunct therapy.

#### 4.5.1. Medical Regimen (Antibiotics and or Antiseptic Mouthwash)

There is inconsistency and diversity in the protocol and effectiveness of medical therapy among the scientific community and available literature in the treatment of MRONJ.

Different types, doses, route of administration, frequency and duration of antibiotics combined with antiseptic mouth rinse have been utilised with different protocols either pre- and post-treatment or only pre- or only post-treatment (Table 7), at different MRONJ staging, either as a single or combined therapy with CS + PBM or SL + PBM or SC or PBM or CS + LS + Piezo + PRF + PBM or CS + PRF + PBM or CS + LS + PRF + PBM or CS + Piezo + PRF + PBM. Studies showed statistically significant improvement in combined Med + PBM protocol than Med + CS + PBM and Med + SL protocols among oncology and non-oncology cohort [113].

Several studies suggested antibiotics should be given either for one week [104,106], ten days [127], 14 d [107,108,109,110,111,112,113,114], three or four weeks, or until complete mucosal healing [167].

In recent treatment guidelines, there was a consensus that prolonged antibiotic treatment was indicated in MRONJ patients with signs of infection, i.e., in all patients with MRONJ stage II or III [27,89]. Contradictory, a study conducted by De Bruyn et al. (2018) [168] reported no significant advantage of metronidazole and doxycycline treatment in patients with MRONJ and concluded the total bacterial level in MRONJ patients was higher even when treated with systemic antibiotics significantly different bacterial amounts of the selected species, suggesting an alteration in the microbial population. This was supported by studies [169,170] reporting various bacterial species associated with MRONJ, such as *Aggregatibacter actinomycetemcomitans*, *Prevotella* spp., *Fusobacterium* or *Capnocytophaga* spp., *Streptococcus mitis*, *Streptococcus gordonii*, *Actinomyces odontolyticus* and *Veillonella* spp.

Medical protocols (antibiotics and antiseptic mouthwashes) have been utilised as standard pitfalls associated with long-term antibiotic use, resulting in the development of antimicrobial resistance (AMR) and cumulative risk of adverse events [171]. The latter was reported in a study conducted by Walker et al. (2019) [172] highlighting frequent colonisation of Gram^-ve^ bacteria in necrotic bone lesions, and such bacteria are known to have a high probability of intrinsic or acquired resistance toward penicillin. Interestingly, a study conducted by Ji et al. (2012) [173] reported antibiotics should not be abused at any MRONJ stage because infection does not directly lead to ONJ development.

Another limitation in the included studies of the present review was the heterogeneity in reported antibiotics indications and regimens. Although this limits our ability to draw specific conclusions, it reflects the real-life challenges of managing these heterogeneous and often medically complex patients on long-term antibiotics. Our study highlights the importance of susceptibility testing, as recommended by the AAOMS [58] and Multinational Association of Supportive Care in Cancer (MASCC)/International Society Oral Oncology (ISOO)/American Society Clinical Oncology (ASCO) [89].

Since most patients are treated at outpatient setting, antibiotic treatment should not only be effective against Gram^-ve^ bacteria, but also provides a good oral bioavailability and hence, currently a routine use of fluoroquinolones (moxifloxacin or ciprofloxacin) instead of penicillin in MRONJ patients with stage II or III of the disease. Nevertheless, the use of fluoroquinolones can have severe side effects, especially in elderly patients and those with multiple comorbidities.

Using routine susceptibility testing may help to avoid using fluoroquinolones in cases where it may not be necessary. If antibiotic treatment is warranted in patients with MRONJ, the empiric choice of antibiotics should consider the high rate of Gram^-ve^ bacteria, or utilisation of cultivation methods as a guide for antibiotic treatment.

The frequent use of antibiotics especially clindamycin or amoxicillin in dental or oral surgical procedures can lead to an increase in the bacterial resistance, resulting in serious consequences, especially in patients with MRONJ. Hence, antibiotic treatment should be reconsidered in each case and each patient (case-dependent decision), especially in MRONJ patients. Therefore, further research is warranted to evaluate and develop potentially more rational antibiotic therapies, with a special emphasis on the efficient antibiotic delivery to the hypovascular bone matrix [174].

The AAOMS’ guidelines [27] demonstrated that rinsing the mouth with different antiseptic solutions for 10 min results in various effects on the bone samples. For example, rinsing with CHX significantly maintained a higher cell viability and protein release of growth factors, which are potent to the bone remodelling cycle [175]. The percentage of CHX at value of 0.2% or povidone iodine (PI) at value of 0.5% increases the cellular viability and the release of potent growth factors, inducing bone remodelling and angiogenesis; hence, this was recommended as an antiseptic-rinse protocol [175]. In the present review, 0.2% CHX mouth rinse pre- and post-operatively protocol was employed in the majority of the included studies [104,105,106,108,109,113], which was aligned with AAOMS guidelines.

#### 4.5.2. Autologous Platelet Concentrates (APCs)

The scientific evidence in utilising APCs as a single therapy (preventive or therapeutic approach) in MRONJ management remains debatable. The results of a recent systematic review that was conducted by Fortunato et al. (2020) [176] did not provide unequivocal findings on the effectiveness of this therapy but did not exclude further studies that can improve otherwise. This was supported by two studies concluded that there was an uncertain true effect of APC in MORNJ management; hence, the overall level of evidence is low [177,178].

PRP was utilised in two studies [107,109], as an adjunctive therapy to various treatment protocols as follows: G1b: Med + CS + PRP + PBM; G1c: Med + CS + SL + PRP + PBM; G1d: Med + CS + Piezo + PRP + PBM; G1e: Med + Piezo + PRP + PBM; G1f: Med + S + PRP + PBM; and G1g: Med + SL + Piezo + PRP + PBM [107] and Med + CS + Piezo + PRF + PBM protocol [109]. Hence, it would be difficult to conclude whether PRP has added a value to the clinical outcomes. Well-designed RCTs are warranted to justify this.

#### 4.5.3. Surgical Approaches

Despite several surgical approaches (CS, LS and piezo) were utilised for various MRONJ staging for therapeutic strategy, this treatment modality remains controversial.

The surgical approach indeed allows ablation of necrotic tissues with minimal regenerative and restorative capacity, which can interfere with wound healing [179]. Nevertheless, a major challenge of the surgical treatment is to differentiate between the viable and necrotic bone [180], which allows a minimal amount of bone removal, facilitates healing with minimal jawbone weakening and maximises dental prosthetic rehabilitation [180]. Fluorescence-guided surgery can be helpful in determining the resection margins; however, it did not improve patients’ QoL instead of CS techniques [181]. On this note, importantly to mention that surgeon’s skill and expertise are crucial to achieve minimally required necrotic bone removal [180].

A 2940 nm surgical laser and piezo surgical tool offer a minimally invasive ablation of the necrotic bone without compromising the integrity of the adjacent healthy bone, but they are slower compared with the standard drill machine [182].

There is no clear guidance on how to determine the surgical plan for patients with MRONJ stages II and III for whom conservative treatment was ineffective. Also, there is no defined guidelines to determine the incisal margin safety and soft tissue management. Hence, CS is advocated [183]. In the present review, the following three surgical approaches were implemented as a single or combined therapy: CS, piezo surgery and SL (2940 nm).

Despite knowing the surgical approach offers good results for cohort with stage I and II ONJ aiming to control the long-term effects of the disease [184,185], but it does not allow wound healing and closure [179]. It is advised to manage the disease, as conservative as possible, since the surgical management is not always successful in creating a new surgical site in avascular region.

Reduced angiogenesis at MRONJ site compromises the access for monocytes/macrophages and infection-fighting cytokines, reaching the affected area [186]. Hence, different therapies are recommended depending on MRONJ staging defined by the AAOMS. Conservative treatments can be performed in cases of early stages (I and II) of MRONJ and in less complex cases. Whereas surgical treatment is more frequently performed in advanced stages of MRONJ (stage III) or when medical therapy is ineffective to improve stage II symptoms [89,187]. Nevertheless, this pattern has increasingly been questioned and debated.

Ewald et al. (2021) [174] reported systemic antibiotic treatment is a key component in the treatment of MRONJ stage II and III, according to the recent guidelines and recommendations. The complete removal of the necrotic bone, smoothing sharp bony edges and obtaining a complete wound closure, accompanied by perioperative antibiotic treatment, are generally considered to be the most suitable approach to achieve ONJ healing [23,76,77,87]. This needs to be considered with caution, taking into account the evidence the AMR that compressively explained in subheading 4.5.1.

It remains unclear which subset(s) of patients can derive most benefit from CS and hence multiple treatment approaches, as a single or combined therapy were as follows: Med, SL (2940 nm), APC, PBM, piezo surgery, ozone, exhibited promising results in facilitating the healing process. The current clinical practice guidelines predominantly recommend antibiotic therapy as a non-surgical approach, primarily to address secondary infections in the necrotic areas. Adjunct PRP therapy to CS tends to improve the recurrence rate with good healing in 85–90% of the cases [177,178,188].

Med + ozone + CS + LS treatment protocol achieved complete clinical and radiographic recovery (100%), with a complete remission of osteonecrosis [189]; hence, ozone therapy can also be used in patients with stages II and III of MRONJ, where surgical approach is not suitable and prolonged Medication therapy is unadvisable especially in medically compromised oncology cohort. It is noteworthy that an average of 7.6 months of complete remission was reported with surgical treatments compared with a period between 13 and 19 months in conservative treatments [190].

The mean duration of the lesion healing time in the eligible studies of the present review was 40.24 months in 83.33% of them and the remaining 16.66% failed to mention. Also, no significant association was observed between the outcomes and MRONJ localisation and stage, drug treatment duration, gender, *DM*, corticosteroid therapy, smoking habits, underlying disease and history of CT at both the three- and six-month follow-up. Contradictorily, a study that was conducted by Khan et al. (2015) [165] reported that multiple variables, such as age, gender, disease status, MRONJ stage and lesion size, played a role in MRONJ outcomes. This controversial could be due to the subjects’ heterogeneity associated with different systemic diseases, comorbidities (e.g., *DM* type II), local factors, mixed oncology (various type of tumour) and non-oncology (osteoporosis, Paget disease).

#### 4.5.4. Therapeutic PBM

According to the Clinical Practice Guidelines of MASCC/ISOO/ASCO, for patients with confirmed MRONJ, the treatment goal is to alleviate pain, control infection in soft and hard tissues and decrease the progress or occurrence of ONJ [89]. Hence, PBM can be considered an ideal therapy in modulating pain intensity [60,61,62,63,64], prompting wound healing [191] and regulating cell metabolism [117]. In the present review, several treatment protocols were employed in which therapeutic PBM was utilised as a monotherapy or combined to one or more of the treatment modalities mentioned in Section 4.5.

It is important to highlight that one study employed PBM as a monotherapy [103], and two studies utilised PBM either as an adjunct therapy to combined Med + CS protocol, [104] or to multiple therapies protocols, Med + CS + Piezo + PRF [109].

A study conducted by Nica et al. (2021) showed the overall success of PBM as a monotherapy with a complete wound healing (96%) was reported in 241 patients who were exposed to antiresorptive or antiangiogenic therapy and developed MRONJ at various stages. Of the 108 patients treated with CS + Med + PBM, 96% resolved, with only one case progressing [125], indicating PBM effectiveness as a single therapy or combined in achieving a complete wound closure. This is in agreement with a study conducted by Scoletta et al. (2010) [103], utilising PBM as a monotherapy for mixed cohort (oncology and non-oncology), which significantly decreased the fistula after 4 weeks, where 60% improved and 40% stable at one-month follow-up with no adverse effects reported. However, at 8 months, 85% reported stable. In this context, a study conducted by Altay et al. (2014) [104] utilised PBM + Med + CS protocol for oncology cohort and reported 100% lesion resolution and another study [109] employed PBM to Med + CS + Piezo + PRF protocol for oncology cohort reported similar results (100% resolution). This is in agreement with another study conducted by Tenore et al. (2020) [115].

The above-mentioned notes indicate the therapeutic PBM as an adjunct to Med + CS protocol or Med + CS + Piezo + PRF protocol for oncology cohort have optimised the clinical outcomes to 100% MRONJ lesion resolution compared with PBM as a monotherapy.

Taking into account the adverse effects of antibiotic resistance, aPDT has emerged as a viable alternative to conventional antimicrobial agents due to its ability to modulate metabolic, biochemical, photophysical and inflammatory processes and promote analgesia without the adverse effects of drug resistance.

aPDT and PBM attenuate the severity of ONJ, promote reduction of necrotic lesion expansion and MRONJ downstage from III to II. They also reduce the inflammatory activities of the macrophages and T lymphocytes and upregulate cytokines that stimulate cell proliferation and differentiation [192]. Importantly, a complete mucosal closure is crucial in osteonecrosis lesion healing rather than bone healing. Hence, PBM can improve primary soft tissue healing by IL-1RA-mediated tissue inflammation inhibition and epithelial cell migration, resulting in promoting the underlying osseous tissue repair and preventing MRONJ development. Additionally, PBM plays a crucial role in regulating transforming growth factor β1 (TGF-β1) signalling pathway, which relatively certain is involved in MRONJ development [193,194,195]. Extensive studies to explore the latter is important to optimise clinical outcomes.

A study conducted by Abdolrahmani et al. (2024) [67] reported combined Pentoxifylline and alpha tocopherol (PENT-E), teriparatide, PBM, aPDT and the use of growth factors have shown to enhance tissue healing in MRONJ patients. Implementing these methods alone or in conjunction with surgical treatment has been linked to reduce discomfort, improve wound healing and enhance bone neoformation. Further studies are required to validate this protocol.

Taking the above-mentioned notes into consideration, we summarised the therapeutic approach for MRONJ and, hence, suggested recommendation based on evidence-based science and practice in utilising PBM as adjunct therapy to combined CS + aPDT +/− piezo and PRF (oncology and non-oncology cohorts).

### 4.6. Assessment of the Reported PBM Parameters

Controversies remain on tissue biostimulation induced by laser irradiation due to a lack of uniform reported physical and biological variables, such as type of laser, output power, frequency of light pulse, fluence, time of application, distance of source from the irradiated tissue and histological differences between treated tissues; thus, summarising the results is extremely difficult. Also, the molecular factors such as light absorption by mitochondrial enzymes, cytochromes, flavins are poorly reported.

In terms of therapeutic PBM in oncotherapy associated bone necrosis, WALT recommends intraoral visible (red 630–680 nm) or transcutaneous NIR (800–1100 nm) wavelength LED/laser device with a power density (treatment surface irradiance) of 10–150 mW/cm^2^ for a total dose of 2 Einstein (photon fluence at 810 nm = 9 p.J/cm^2^) per treatment field performed [196]. They stated that “*the treatments should be repeated 3 to 4 times a week for 4 to 6 weeks, or until clinical benefit is evident*”. The latter more or less coincides with the findings of the present review where the treatment protocol was five sessions per week either for 6 weeks or for 8 weeks.

Interestingly, in terms of wavelength, the majority of the included studies in the present review utilised 1064 nm [105,108,109,110,111,112,113,114], but the remaining studies utilised 808, 810 and 909 nm (Table 6). There was no distinguished protocol whether the cohort was oncology or non-oncology within each interventional arm. Moreover, none of the included studies utilised intraoral visible laser light (WALT recommendations), but all of them utilised NIR (808–1064 nm) intraorally. Nevertheless, we suggest in advanced cases of MRONJ (stage III) where extraoral sinus or fistula is present, an extraoral PBM approach can be employed based on WALT recommended dosimetry noted above.

It is noteworthy that a study conducted by Merigo et al. (2018) [107] reported a fluence value of 21231 J/cm^2^ (theoretically) for PBM. We need to emphasise that such a fluence value is not possible for PBM, as it would definitely generate thermal responses. Equally, the following PBM irradiance values (W/cm^2^) would generate thermal effects: 1262.5 [110], 268.81 [111], 268.57 [112], 268.81 [113] and 1562.5 [114].

Taking all the above-mentioned notes into account, we extrapolated the laser dosimetry of only two out of twelve included studies in the present review [104,105], reporting the most required parameters and are within the range of the WALT recommendations. The laser parameters are as follows: 808 nm (laser), 0.5 W, CW, 3 s/spot, 1.4 J/spot, 5 J/cm^2^, 0.28 cm^2^, laser–tissue distance was ranged between 0.5–1 cm [104] and 1064 nm, 0.25 W, pulsed, 10 Hz, 1.25 J, 60 s, 6.25 J/cm^2^, 0.4 cm^2^; and laser–tissue distance was 4 cm [105]. Based on the latter, we calculated the suitable laser dosimetry for future RCTs, which are as follows: 808 nm at 5 J/cm^2^ is 7.5 p.J/cm^2^ which is 1.7 Einstein. Moreover, 1060 nm at 6.5 J/cm^2^ is also 7.5 p.J/cm^2^, which is 1.7 Einstein.

### 4.7. Preventive Protocol Strategy

MRONJ detection and diagnosis at an early stage to avoid the risk of progression and effectively prevent its occurrence are the fundamental keys for MRONJ management, by screening high-risk patients (AAOMS) [89].

The diagnosis and prevention of ONJ play a significant role not only in QoL improvement in MRONJ cohort, but also in decision-making process among the majority of Multidisciplinary team (MDT) (doctors, dentists, oncologists and dental hygienists), to screen high-risk patients [89,197].

#### 4.7.1. Preventive PBM

The prevention of MRONJ has been indicated as a crucial factor in those patients receiving ART. In the present review, 3 out of 12 studies [108,110,114] employed preventive approach in MRONJ management based on one group in each study, and their protocols were as follows: PBM as a monotherapy [108], PBM as an adjunct to CS + Med in two studies [110,114]. The results were positive.

Based on the above-mentioned notes, PBM therapy as preventive measure to avoid MRONJ following MOS procedure exploit biostimulation of the mitochondrial cells induced by the irradiation, resulting in improved healing of both hard and soft tissues. After tooth extraction, however, bone directly exposed to the oral environment is vulnerable to colonisation by organised microbial biofilms. Such polymicrobial communities composed of bacteria and occasionally yeast, fungi and viruses embedded in extracellular polymeric substance, have been found in bone specimens from sites affected by MRONJ [120]. Therefore, aPDT can be effective prior to the dental procedure as a preventive measure [120].

MDT should collaborate and work with patients at an early stage of this disease, addressing the risk factors for MRONJ. Additionally, oncologists, dentists and dental specialists should be practised at every period of treatment, sharing treatment information contemporaneously. Preventive measures of preoperative professional oral hygiene and antimicrobial mouthwashes, as well as post-operatively, until complete mucosal healing [167,198,199,200]. Additionally, to optimise the preventive approach of MRONJ, a meticulous physical and subjective examination, periodic correction of dental prostheses, as well as any necessary dental treatments are necessary to prevent or minimise the risk of ONJ. This is illustrated in Section 4.7.2’s subheading.

#### 4.7.2. Oral and Dental Care Pathways

Primary prevention aims to eliminate oral and dental risk factors by focusing on restoring and/or maintaining good oral health, thereby reducing the risk of pathological conditions or other adverse events. MRONJ prevention should be tailored, considering the individual patient’s risk of MRONJ, frailty and life expectancy. It is essential to identify and remove all oral conditions that are known to trigger MRONJ and restore meticulous oral health, starting pre-treatment and continuing throughout the course of BPs therapy. Oral surveillance and appropriate dental care should be prolonged after drug cessation, due to its known long-standing inhibition of jawbone remodelling [89,197]. The authors summarised the oral and dental pathway protocols in Table 15.

### 4.8. Limitations of the Quantitative Analysis

A very first systematic review was by Rupel et al. (2014) [203] aimed to identify different treatment approaches for bisphosphonate related osteonecrosis of the jaws (BRONJ) that are reported in literature and to assess healing rates for each category of treatment and for each BRONJ stage. The authors found out that the overall outcome results and results for every disease stage were the highest when patients were treated with extensive surgery or extensive laser assisted surgery.

Another systematic review was conducted by Weber et al. (2016) [204] aimed to assess the efficacy of laser therapy in the management of BRONJ and concluded in their review that combined treatment with antibiotics, minimally invasive surgery (including Er:YAG LS) and PBM therapy in the early stages of the disease should be the gold standard for bisphosphonate-related osteonecrosis of the jaw management.

A systematic review conducted by Li et al. (2020) [205] assessed the effectiveness of laser-assisted treatments for MRONJ. The authors found that PBM therapy using visible and infrared GaAs laser demonstrated significant differences in pain scores across the included studies. The effectiveness of other laser assisted treatments was uncertain [205]. They concluded that more RCTs of a good quality with a low risk of bias are needed to examine whether laser-assisted treatment should be a routine part of management of patients with MRONJ.

A recent systematic review with a pooled analysis was conducted by Di Fede et al. (2021) [141] in order to compare MRONJ surgical techniques (conservative or aggressive) versus combined surgical procedures (surgery plus a non-invasive procedure). They found that a statistically significant difference in the 6-month improvement rate, comparing combined conservative surgery versus only aggressive (91% versus 72%, *p* = 0.05), was observed [141]. No significant difference regarding any group with respect to the 6-month total resolution rate (82% versus 72%) was demonstrated [141].

Another key finding was that CS combined with various, adjuvant, non-invasive procedures (ozone, PBM, or blood component + LS) were reported to achieve partial or full healing in all stages, with improved results and the improvement of many variables. The reviewers further emphasised on the need for more well-controlled studies on this topic in order to obtain definitive results [141].

Razavi et al. (2022) [206] aimed to assess the efficacy of adjunctive PBM in the management of MRONJ. Based on their findings, the authors concluded that PBM, as an adjuvant therapy, can significantly improve the outcomes of each treatment plan; however, surgical intervention for the complete healing of the lesions is suggested.

The results of our systematic review analysis are in accordance with the existing research on this subject. A common agreement amongst all reviewers is the lack of well controlled trials with robust methodology quality and long-term follow-up. Till date, a meta-analysis on this topic remains unaccomplished. The high heterogeneity and lack of uniformity in the methodology of the available research along with high levels of risk of bias affecting the quality of the conducted research are a few major concerns that may be accountable for the lack of quantitative analysis existing on this topic.

### 4.9. Suggested Rationalised Recommendations and Consensus for Future RCTs

Addressing the results of all the subheadings in this discussion section, it is evident there is a discrepancy in the methodology, diagnostic and outcomes assessment criteria, which undoubtedly have a great impact on the optimal outcomes of the treatment commensurate with statistically significant inconsistency, despite significant MRONJ improvements and stage downscaling at follow-up timepoints. Importantly, these methodological inadequacies can increase the risk of inconsistency of the general guidelines worldwide, resulting in lack of effectiveness in handling patients at risk or affected by MRONJ.

We employed Level of Evidence assigned to address the above-mentioned indications grounded by the study design, according to Somerfield criteria [207].

A suggestion was possible for lower-level evidence, only when consistent evidence from multiple studies and panel consensus on the interpretation of this evidence. Thus, to enable maximal, practical clinical use and promote future research for PBM in MRONJ. Hence, the results of the included studies were categorised as “*suggested*” levels (IV, V and panel consensus) as follows: Level IV [103,104,105,106,107,108,109,110,111,112,113] and Level V [114].

Based the Somerfield criteria, the authors suggested rationalised consensus and recommendations for robust and valid methodology for future multi-centre well-designed RCTs based on the Level of Evidence of the extrapolated data and the available scientific literature. This is illustrated in Table 16.

## 5. Conclusions

The present systematic review, for the first time, showed that PBM, as a monotherapy or as a primary adjunct therapy to aPDT or to any standard treatment(s), is a promising modality for therapeutic or preventive approach in MRONJ management.

The authors concluded that the preventive approach in MRONJ management is significant in prompting bone healing, minimising complications and enhancing patient’s QoL; hence, they recommend this approach. Moreover, MDT consultation and patients’ oral and dental assessment, as well as disease awareness prior to ART commencement, play a crucial role in minimising ONJ complications. Moreover, the author suggested bone and salivary biomarkers that can be utilised for MRONJ risk prediction. Nevertheless, robust studies are warranted to validate the most suitable predictive biomarkers for ONJ risk.

The authors established a proposed PBM laser dosimetry protocol for future RCTs, for the first time, which is as follows: 808 nm at 5 J/cm^2^ is 7.5 p.J/cm^2^, which is 1.7 Einstein; 1060 nm at 6.5 J/cm^2^ is also 7.5 p.J/cm^2^, which is 1.7 Einstein.

As there are no clinical RCTs in the current scientific literature employing PBM as a single or adjunct modality (preventive or therapeutic) for oncology or non-oncology cohort in MRONJ management, the authors produced suggested a rationalised consensus and recommendations, for the first time, as a guide for future multi-centre, well-designed, prospective clinical RCTs with a long-term follow-up.

## Figures and Tables

**Table 1:** AAOMS staging for MRONJ, 2022 [58].

Category	Description
**Stage 0**	No apparent necrotic bone in asymptomatic patients who have been treated with IV or oral ART. Patients with no clinical evidence of necrotic bone but who present with non-specific symptoms or clinical and radiographic findings, such as the following: **Symptoms** Odontalgia not explained by an odontogenic cause. Dull, aching bone pain in the jaw, which may radiate to the temporomandibular joint region. Sinus pain, which may be associated with inflammation and thickening of the maxillary sinus wall. Altered neurosensory function. **Clinical findings ** Loosening of teeth not explained by chronic periodontal disease. Intraoral or extraoral swelling—radiographic findings. Alveolar bone loss or resorption not attributable to chronic periodontal disease. Changes to trabecular pattern sclerotic bone and no new bone in extraction sockets. Regions of osteosclerosis involving alveolar bone and/or the surrounding basilar bone. Thickening/obscuring of periodontal ligament (thickening of the lamina dura, Sclerosis and decreased size of the periodontal ligament space).
**Stage I**	Exposed and necrotic bone or fistula that probes to the bone in patients who are asymptomatic and have no evidence of infection/inflammation. these patients also may present with radiographic findings mentioned for Stage 0 that are localised to the alveolar bone region.
**Stage II**	Exposed and necrotic bone or fistula that probes to the bone, with evidence of infection/inflammation. These patients are symptomatic and may present with radiographic findings mentioned for Stage 0 localised to the alveolar bone region.
**Stage III**	Exposed and necrotic bone or fistulae that probes to the bone, with evidence of infection and one or more of the following: Exposed necrotic bone extending beyond the region of alveolar bone (i.e., inferior border and ramus in the mandible, maxillary sinus and zygoma in the maxilla). Pathologic fracture. Extraoral fistula. Oral antral/oral–nasal communication. Osteolysis extending to the inferior border of the mandible or sinus floor.

**Table 2:** Tabular representation of eligible non-randomised clinical studies in terms of demographic characteristics, primary diagnosis, lesion sites and numbers, type of dental trauma and timing and symptoms’ onset. Abbreviations: NM, not mentioned; M, male; F, female; BMC, bone metastatic cancer; EO, extraoral; Patho, pathological; yrs, years; Max, maxilla; Mand, mandible; OS, oral surgery; MOS, minor oral surgery; PDD, periodontal disease; LC, lung cancer; BC, breast cancer; PC, prostate cancer; NPC, nasopharyngeal cancer; RA, rheumatic arthritis; DM, *Diabetes mellitus*; CT, chemotherapy; EOF, extraoral fistula; KC, kidney carcinoma; BM, bone metastasis; DVT, deep venous thrombosis; HTN, hypertension; AC, anticoagulant; NA, not applicable; Y, yes; pt, patient.

Study Reference	Mean Age (yrs)	Sample Size pt/Lesion (M/F)	Smoking	Underlining Diseases	Associated Treatments	Primary Diagnosis	Site/s of Lesion/No. of Lesion	Initial MRONJ Staging	Type of Dental Trauma/Timing	Symptoms’ Onset
[103]	71.3	20 [6(30%)/14 (70%)]	2/20 (10%)	NM	Steroids CT	BC: 6/20 (30%)/ MM 6/20 (30%)/osteoporosis 5/20 (25%)/ PC: 3/20 (15%)	NM	Stage IA 1/20 (5%)/Stage IB 1/20 (5%)/Stage IIA 13/20 (65%)/Stage IIB 3/20 (15%)/Stage IIIA 2/20 (10%)	Dental extractions: 15/20 (75%); PDD: 2/20 (10%); Prosthetic trauma: 1/20 (5%); Dental implant: 1/20 (5%); MOS 1/20 (5%)	EO oedema; pus; EOF; halitosis; bone exposure; pain; patho fracture; EOF; asymptomatic
[104]	64.1	11 (7/4) 63.63%/36.36%	NM	NM	NM	BC: 3/11 (27.27%); MM 4/11 (36%); PC: cancer 3/11 (27.27%); LC: 1/11 (9.09%)	Max: 7/11 (63.63%); Mand: 2/11 (18.18%); max and Mand: 2/11 (18.18%)	Stage II 9/11 (81.81%); Stage III 2/11 (18.18%)	Extraction 9/11 (81.81%) Denture irritation 2/11 (18.18%)	Pain; necrotic bone; granulation tissue; OAC
[105]	55.4	20 [7 (35%)/ 13(65%)]	NM	NM	NM	BC: 1/20 (5%); PC: 1/20 (5%); MM 7/20 (35%); neuro-endocrine tumour 1/20 (5%)	20/20 Max: 11/20 (55%); Mand: 9/20 (45%)	Stage I 6/20 (30%) Stage II 14/20 (70%)	MOS 100%	Necrotic bone exposure
[106]	71	106/131 [32 (30.18%)/74 (69.811)]	NM	NM	Steroids	Oncology: 72.51% non-oncology: 27.48%	Max: 46/131(35.11%) Mand: 85/131 (64.88%)	Stage I 11/131 (8.39%); stage II 65/131 (49.61%); stage III 55/131 (41.98%)**/**(34.57%)	Spontaneous 44/131 (33.58%); OS: 87/131 (10.68%)	Bone exposure; pus discharge; tooth mobility
[107]	72.6	21[5 (23.80)/16 (76.19%)]	3/21 (14.28%)	HTN; arrythmia; DVT; DM (NM, type);	Steroids	Solid tumour: 11/21 (52.38%). Osteometabolic diseases: 10/21 (47.61%)	Max: 6/21 (28.6%); Mand:15/21 (71.4%)	Stage I 2/21 (9.6%); Stage II 15/21 (71.4%); Stage III 4/21 (19%)	Dental Prosthesis 8/21 (38.05%); Dental implant 1/21 (4.76%); NM 12/21 (57.14%)	NM
[108]	66.3	44 [12 (27.27%)/32 (72.72%)]	7 /44 (15.90%)	DM (NM, type)	Steroids	BPs for oncology: 21/44 (42.72%): BC: 14/21 (66.66%); PC: 6/21 (28.57%), NPC: 1/21 (4.76%); BPs for non-oncology: osteoporosis: 23/44 (52.27%)	NA	NA	NA	NA
[109]	68.04	21 (7/14) 33.33%/66.66%	6/21 (28.57%)	DM (NM, type)	Steroids CT	BC: 14/21 (66.66%); PC: 3/21 (14.28%); LC: 2/21 (9.52%): KC: 1/21 (4.76%); MM: 1/21 (4.76%)	Max: 8/21 (38.05%) Mand: 13/21 (61.90%)	Stage II 15/21 (71.42%) Stage III 6/21 (28.57%)	Extraction 18/21 (85.71%); Dental implant 2/21 (38.05%); Prosthesis pressure: 1/21 (4.76%)	Bone exposure; Swelling; inflamed mucosa; OAC
[110]	68.5	36 (12/24) 33.33%/66.66%	NM	NM	Steroids	BM: 18/38 (50%); MM 11/36 (30.55%); osteoporosis 7/36 (19.44%)	NA/ 82 Extractions/ Max: 31/82 (37.80%)/Mand: 51/82 (62.19%)	NA	NA	NA
[111]	NM	128 (33/95) 25.78%/74.21%	26/128 (20.31%)	DM: 7.03%	Steroids	MM 52/128 (40.62%) BM: 53/128 (41.40%) Osteoporosis 23/128 (17.96%)	Max: 30/128 (23.43%); Mand: 85/128 (66.4%); Max + Mand: 13/128 (10.15%)	Stage I 17/128 (13.28%);; Stage II 92/128 (71.87%); Stage III 19/128 (14.84%)	NM	Bone: Exposed/ unexposed; OAC
[112]	67	91(25/66) 27.47%/ 72.52%	NM	NM	CT	MM 39/91 (42.85%); BCM: 33/91 (36.26%); Osteoporosis 16/91 (17.58%); NM: 3/91 (3.26%); 1 pt bilateral femur bone necrosis	Max: 21/91 (23.07%) Mand: 62/91 (68.13%) Max+ Mand 8/91 (8.79%); Treated lesions: Max: 10/55 (18.18%); Mand 45/55 (81.81%)	Stage I 8/91 (8.79%); Stage II 66/91 (72.52%); Stage III 17/91 (18.68%)	Spontaneously 41/91 (45.1%); After surgical procedures 50/91 (54.9%)	Bone exposure; Pain; Swelling; Pus discharge; Halitosis; Paraesthesia
[113]	67.3	190 (52/138) 27.36%/ 72.63%	39/190 (20.5%)	DM:11.5%	Steroids	MM 62/190 (32.63%); BM: 85/190 (44.73%); Osteoporosis 43/190 (22.63%)	Max: 53/190 (27.89%); Mand: 120/190 (63.15%); Max+ Mand: 17/190 (8.94%)	Stage I 34/190 (17.89%); Stage II 126/190 (66.31%); Stage III 30/190 (15.78%)	NM	Necrotic bone exposure; inflamed mucosa; OAC
[114]	68.72	217 (38/179) 17.51%/ 82.48%	Y (pt. no. NM)	DM; vascular disease; renal failure	Steroids; chemo; hormonal; AC	MM 23/217 (10.59%); BM: 72/217 (33.17%); non-oncologic: (osteoporosis; RA; Paget’s disease) 122/217 (56.22%)	NA	NA	NA	NA

**Table 3:** Representation of treatment approach, interventional groups, number of recruited subjects, the total number of lesions in each included study and its distribution in each affected site. Also, the total number of lesions in all the included studies, as well as the percentage of lesions in each site for all the eligible studies. Abbreviations: Med, medications; CS, conventional surgery; LS, laser surgery; PRP, platelets rich plasma; PBM, photobiomodulation; G1, group1; G2, group 2; G3, group 3; G4, group 4; G5, group 5; pt, patient; No., number; Max, maxilla; Mand, mandible.

Treatment Approach	Study Reference	Group No.	Treatment Protocol		No. of Treated pt.	Lesion No.		Lesion Distribution in Affected Site		
								Max	Mand	Combined Max & Mand
Preventive	[108]	G1	PBM		20	NA		NA		
	[110]	G1	Med + CS + PBM		36			NA		
	[114]	G1	Med + CS + PBM		217			NA		
Therapeutic	[103]	G1	PBM		20	NM		NM	NM	NM
	[104]	G1	Med + CS + PBM		11	15		2	7	6
	[105]	G1	Med + SL + PBM		20	20		11	9	0
		G2	Med + CS							
	[106]	G1	Med + CS		106	131		46	85	0
		G2	Med + PBM							
	[107]	G1	G1a	Med + CS + LS + Piezo + PRP + PBM	21	21		6	15	0
			G1b	Med + CS + PRP + PBM						
			G1c	Med + CS + LS + PRP + PBM						
			G1d	Med + CS + Piezo + PRP + PBM						
			G1e	Med + Piezo + PRP + PBM						
			G1f	Med + LS + PRP + PBM						
			G1g	Med + LS + Piezo + PRP + PBM						
	[109]	G1	Med + CS + Piezo + PRF + PBM		21	21		8	13	0
	[111]	G1	Med		128	12	101	30	85	13
		G2	Med+ PBM			27				
		G3	Med+ CS			17				
		G4	Med + CS + LS + PBM			45				
	[112]	G1	Med		48	13	55	10	45	0
		G2	Med + PBM			17				
		G3	Med + CS			13				
		G4	LS + PBM			12				
	[113]	G1	Med		190	32	166	53	120	17
		G2	Med + PBM			37				
		G3	Med + CS			17				
		G4	Med + CS + PBM			39				
		G5	Med + LS			41				
Total of lesions in all the eligible studies							530	166	379	36
% of total lesions in each affected site								28.57	66.23	6.19

**Table 4:** Representation of BP duration and drug holiday and its duration. Abbreviations: BPs, bisphosphonates; NM, not mentioned; G1, group 1; Y, yes; N, no; IV, intravenous; PO, orally.

Study Reference	Duration of BP Medication in Months (Range)	BPs Drug Holiday					Duration-BPs Drug Holiday-Month
		Y	N	All Subjects	Few No. of Cohort “Y”	NM	
[103]	42.95 ± 32.16	-	x	x	-	-	NA
[104]	21.27 (9–629)	x	-	x	-	-	Until complete mucosal healing
[105]	32.35 (6–132)	x	-	-	19/20	1/20	4.5
[106]	NM	x	-	-	85/106	21/106	3 pre-op in G1 (85/106)
[107]	8/21 subjects-NM 13/21 subjects—54.53 (5–164)	NM					
[108]	IV—44.6 (25–108); PO—36.3 (18–96)	-	x	x	-	-	NA
[109]	64.76 ± 21.53 (39–96)	x	-	x	-	-	4.52 ± 1.12
[110]	NM	x	x	-	1/36	34/36	NM
[111]	28 (1–96)	x	x	-	x	x	NM
[112]	25 (2–120)	x	x	-	x	x	NM
[113]	Oncology, 26 ± 20 (3–72); Non-oncology, 90 ± 40 (24–144)	NM					
[114]	Oncology, 17 Non-oncology, 53 (1–92)	x	-	-	49/217	168/217	2 prior and after tooth extraction

**Table 5:** Representation of initial symptoms distribution of the eligible studies. Abbreviations: EO, extraoral; OAC, oroantral communication; EOF, extraoral fistula; BE, bone exposure; PF, pathological fracture; IM, inflamed mucosa.

Study Reference	Asympto-Matic	Pain	Halitosis	EO Oedema	Swelling	IM	Paraest-Hesia	Pus	EOF	BE	Symptomatic/Mobile Teeth	OAC	PF
[103]	x	x	x	x	-	-	-	x	x	x	-	x	x
[104]	-	x	-	-	-	-	-			x	x	x	-
[105]	-	-	-	-	-	-	-			x	-		-
[106]	-	-	-		-	-	-	x		x	x		-
[107]	NM	NM	NM	NM	NM	NM	NM	NM	NM	NM	NM	NM	NM
[108]	NA (preventive)												
[109]	-	-	-	-	x	x		-	-	x	-	x	-
[110]	NA (preventive)												
[111]	-	-	-	-	-	-	-		-	x	-	x	-
[112]	-	x	x	-	x	-	x	x	-	x	-		-
[113]	-	-	-	-	-	x	-	-	-	x	-	x	-
[114]	NA (preventive)												

**Table 6:** Represents the laser dosimetry and treatment protocols of all the eligible studies, including the percentage of the missing data for each parameter. Abbreviations: NM, not mentioned; Hz, frequency; G, group, NA, not applicable; min, minute; W, watt; J, joule; λ, wavelength; d, day.

Study Reference	λ (nm) Laser	Emission Mode	Contact/Non-Contact	Energy (J) per Spot	No. of Irradiation Points	Power Output (W)	Use of Power Meter	Laser–Tissue Distance	Spot Size/Fibre Diameter/Spot Diameter	Energy Density (J/cm²)	Irradiance (W/cm²)	Exposure Time (min/s)	Irradiation Frequency/ Time Interval	Treatment Duration/No. of Sessions
[103]	904	Pulsed/40% duty cycle	Contact	NM	NM	NM	NM	NA	0.8 cm	28.4	NM	NM	1st week: 4 sessions; 2nd week: 3 sessions; 3rd week: 3 sessions	20 d/10
[104]	808	CW	Non-contact	1.4	NM	0.5	NM	0.5–1 cm	0.28 cm² R = 6 mm	5	NM	3 s/point total: 120 s	At day 1, 3, 5, 7, 10	10 d/5
[105]	1064	LP mode/ 10 Hz	Non-contact	2.5	NM	0.25	NM	4 cm	0.4 cm²/950 μm	6.25	NM	1 min	5 sessions for 10 d.	10 d/5 sessions
[106]	810	NM	NM	NM	NM	0.5–1	NM	NM	320 μm	NM	NM	NM	monthly	NM
[107]	808	CW	Non-contact	NM	NM	1	NM	NM	600 μm	Theoretical 21231	NM	1 min	5 times/session	1st session immediately after surgery; then twice a week until mucosal closure.
[108]	1064	15 Hz	Contact	NM	NM	1.25	NM	1–2 mm	320 μm	NM	NM	1 min repeated 5 times	At day; 2,5,7, 10, 14,21,28	1 month/7 sessions
[109]	1064	15 Hz	Non-contact	NM	NM	1.25	NM	1–2 mm	320 μm	NM	NM	1 min, repeated 5 times	At day;2,5, 7,10,14,21,28	1 month/7 sessions
[110]	1064	15 Hz	Non-contact	NM	NM	1.25	NM	2 mm	320 μm	7	1562.5	1 min	Repeated 5 times	Weekly for 1st six weeks + until mucosal closure
[111]	1064	Pulsed/5%; 15 Hz	Non-contact	NM	NM	1.25	NM	2 mm	320 μm	14.37	268.81	1 min	Repeated 5 times	G2: once a week for 2/12; G4: during surgery, then weekly for 2/12
[112]	1064	Pulsed, 15 Hz	Non-contact	NM	NM	1.25	NM	2 mm	320 μm	2.01	268.57	1 min	Repeated 5 times	once a week for 2/12
[113]	1064	Pulsed: VSP/15 Hz	Non-contact	NM	NM	1.25	NM	2 mm	320 μm	14.37	268.81	1 min	Repeated 5 times	Once a week for 2/12
[114]	1064	15 Hz	Non-contact	NM	NM	1.25	NM	2 mm	320 μm	7	1562.5	NM	Repeated 5 times.	6 sessions (once a week), until complete healing
**Missing data (%)**	**0**	**0**	**8.33**	**83.33**	**100**	**8.33**	**100**	**16.66**	**0**	**16.66**	**58.33**	**25**	**0**	**8.33**

**Table 7:** Representation of the medical and antiseptic treatment regimens employed in the eligible studies in terms of type of medications, the dose whether pre-operatively post-operatively or both, route of administration, frequency and duration. Abbreviations: pre-op, pre-operatively; post-op, post-operatively; Po, orally; IV, intravenously; OD, once a day; BD, twice a week; TDS, three times a week; QDS, four times a week; NM, not mentioned; X, yes.

Study Reference	Type of Antibiotics and Antiseptic Mouthwash		Pre- and Post-op		Pre-op	Post-op	Dose	Route of Administration	Frequency	Duration (Day)
[103]	Antibiotics	NA								
	Antiseptics	NA								
[104]	Antibiotics	Amoxicillin clavulanate		x			1000 mg	Po	BD	7
		Clindamycin		x			150 mg	Po	BD	7
		Sulbactam–ampicillin	x				1500 mg	IV	QDS	7
	Antiseptics	Benzylamine hydrochloride Chlorhexidine digluconate		x			0.15% 0.12%	Mouth rinse Mouth rinse	NM NM	NM NM
[105]	Antibiotics	Amoxicillin + Clavulanic acid				x	1000 mg	Po	BD	NM
		Metronidazole				x	500 mg	Po	BD	NM
	Antiseptics	Chlorhexidine gluconate				x	0.2%	Mouth rinse	BD	10
[106]	Antibiotics	Ceftriaxone	x	x			1 g	IM	OD	7
		Metronidazole	x	x			500 mg	Po	BD	7
	Antiseptics	Chlorhexidine	x	x			NM	Mouth rinse	NM	NM
[107]	Antibiotics	Amoxicillin clavulanate	x	x			2 g	Po	OD	14
		Metronidazole	x	x			500 mg	Po	OD	14
		Clindamycin	In case of allergy							
	Antiseptics	NM	NM	NM	NM	NM	NM	NM	NM	NM
[108]	Antibiotics	Amoxicillin + Clavulanic acid	x	x			1000 mg	Po	NM	14
		Metronidazole	x	x			500 mg	Po	NM	14
	Antiseptics	Chlorhexidine digluconate	x	x			0.12%	Mouth rinse	NM	14
[109]	Antibiotics	Amoxicillin + Clavulanic acid	x	x			1000 mg	Po	NM	14
		Metronidazole	x	x			500 mg	Po	NM	14
	Antiseptics	Chlorhexidine digluconate	x	x			0.12%	Mouth rinse	NM	14
[110]	Antibiotics	Amoxicillin	x	x			2 g	Po	OD	14
		Metronidazole	x	x			1 g	Po	OD	14
	Antiseptics	Chlorhexidine digluconate				x	0.2%	Mouth rinse	TDS	Until mucosal healing
[111]	Antibiotics	Amoxicillin	x	x			1 g	Po	BD	14
		Metronidazole	x	x			250 mg	Po	BD	14
	Antiseptics	Chlorhexidine Hydrogen peroxide				x x	0.2% 3%	Mouth rinse Mouth rinse	BD BD	NM NM
[112]	Antibiotics	Amoxicillin	x	x			1 g	Po	TDS	14
		Metronidazole	x	x			250 mg	Po	BD	14
	Antiseptics	Chlorhexidine Hydrogen peroxide				x x	NM NM	Mouth rinse Mouth rinse	BD BD	NM NM
[113]	Antibiotics	Amoxicillin	x	x			1 g	Po	BD	14
		Metronidazole	x	x			250 mg	Po	BD	14
	Antiseptics	Chlorhexidine Hydrogen peroxide	x	x			0.2% 3%	Mouth rinse Mouth rinse	BD BD	NM NM
[114]	Antibiotics	Amoxicillin	x	x			2 g	Po	OD	14
	Antiseptics	Chlorhexidine				x	NM	Mouth rinse	TDS	Until mucosal healing

**Table 8:** Illustrates the outcome variables, diagnostic and outcomes assessment tools. Abbreviations: 1/12, one month; 2–8/12, 2–8 months; 3/12, 3 months; NM, not mentioned; N, no; Y, yes; VAS, visual analogue scale; OPT, orthopantomogram; CBCT, cone beam computer tomography; CTX, c-terminal telopeptide; 1, primary; 2, secondary; CH, complete healing, ICH, incomplete healing; DH, delayed healing.

Study Reference	Outcomes (Variables)			Diagnostic Tools				Outcome Assessment Tools			
	Mucosal Healing	Healing Time	Pain VAS	Clinical Exam + Photos	Imaging (OPT/ CBCT)	Histology	CTX	Clinical Exam + Photos	Imaging (OPT/ CBCT)	Histology	CTX
[103]	CH	1/12	Y	Y	N	N	N	Y	N	N	N
[104]	1° and 2° healing	NM	N	Y	OPT+ CBCT	Y	N	Y	N	N	N
[105]	CH/ICH	NM	N	Y	OPT	N	Y	Y	OPT	N	Y
[106]	CH/DH	NM	N	Y	OPT	Y	N	Y	OPT	N	N
[107]	CH; Recurrence	NM	N	Y	OPT+ CBCT	Y	N	Y	N	N	N
[108]	CH	1/12	N	Y	OPT	N	N	Y	OPT	N	N
[109]	CH	3/12	N	Y	OPT	N	N	Y	OPT	N	N
[110]	Complete healing @ 2 weeks	2–8/52	N	Y	OPT	N	N	Y	OPT	N	N
[111]	CH	NM	N	Y	OPT+ CBCT	N	N	Y	CBCT	N	N
[112]	CH	3/12	N	Y	OPT+ CBCT	N	N	Y	N	N	N
[113]	CH	NM	N	Y	OPT+ CBCT	N	N	Y	CBCT	N	N
[114]	10% DH, 90% NM	NM	N	Y	OPT+ CBCT	N	N	Y	OPT	N	N

**Table 9:** Representation of MRONJ lesion outcomes and staging improvements in relation to the interventional groups for each study and follow-up timepoints. Abbreviations: P, preventive; T, therapeutic; CTX, c-terminal telopeptide; NM, not mentioned; G1, group1; Med, medications; PBM, photobiomodulation; SL, surgical laser; CS, conventional surgery; PRP, platelet-rich plasma; OAC, oroantral communication; 1/12, one month; 3/12, 3 months; 5/12, five months; 8/12, eight months; 9/12, nine months; 33/12, 33 months; 2/52, two weeks; NM, not mentioned.

Study Reference	P/T	Oncology/Non-Oncology/Mixed	Interventional Groups	Follow-Up (Mean Value)/Statistical Significance	MRONJ Staging Improvement	Resolved/Improved/ Stable/Progressive	Recurrence
[103]	T	Mixed	G1: PBM	At 1/12 A statistically significant difference was observed for reported pain, lesion size, oedema and presence of pus and OAC	NM	40% Stable; 60% Improved. No adverse effects reported	No
				At 8/12	NM	85% Stable (symptoms); 80% Stable (lesion size); 10% Progressive (exposed bone); 15% Progressive—new lesion	25%
[104]	T	Oncology	G1: Med + CS + PBM	NM	NM	100% Resolved: 63% healing with primary closure; 36.36% by secondary closure	No
[105]	T	Oncology	G1: Med + SL + PBM G2: Med + CS	Nothing mentioned about the follow-up timepoints. Statistically significant difference in MRONJ stage healing *p* = 0.050. Statistical comparison of treatment-type healing (*p* = 0.370). No significant correlation between CTX and healing status.	Complete healing: Stage I: 16.7%, Stage II: 71,4%; Incomplete healing: Stage I: 83.3%, Stage II: 28.6%	55% Resolved (complete healing) 45% Improved (incomplete healing)	No
[106]	T	Mixed	G1: Med + CS G2: Med + PBM	NM about statistics	G1: 86.5%—stage III to I, 13.5%—III to I	G1: 100%, complete healing—86% resolved and 13.5% improved	One
					G2: 0%—complete healing 2.2%: from stage II to I; and III to II	G2: Complete healing (0%); 87.5% stable, 2.2% Improved; 1.14% Progressive	
[107]	T	Mixed	G1a: Med + CS + SL + Piezo + PRP + PBM G1b: Med + CS + PRP + PBM G1c: Med + CS + SL + PRP + PBM G1d: Med + CS + Piezo + PRP + PBM G1e: Med + Piezo + PRP + PBM G1f: Med + SL + PRP + PBM G1g: Med + SL + Piezo + PRP + PBM	95.23%—complete healing. Statistical analysis: NM	NM	95.23% Resolved	One
				At 9/12	One patient—recurrent—Stage III	4.76% progressive	
[108]	p	Mixed	G1: PBM	At 1/12; No recurrence in long-term follow-up based on clinical and radiological assessment	NA	100% resolved	No
				No significant difference between the variable (age, gender, BPs type/duration and healing time)			
[109]	T	Oncology	G1: Med + CS + Piezo + PRF + PBM	At 3/12: Complete mucosal healing. No significant difference between each variable and delayed healing	100% Resolved, but 3% showed delayed healing		No
[110]	P	Mixed	G1: Med + CS + PBM	At 2/52	100% Resolved	100% Resolved	No
				At 5/12, follow-up: 2 cases developed delayed healing in which one of them was under antiangiogenic therapy	100% resolved, but with 2 cases delayed healing		
				At 33/12 follow-up	100% Resolved	100% Resolved	
[111]	T	Mixed	G1: Med G2: Med + PBM G3: Med + CS G4: Med + CS + SL + PBM	At mean of 3/12	G1: 16.6% transition to stage 0 G2: 33% transition to stage 0 G3: 52.9% transition to stage 0 G4: 88.8% (73.3% permanent transition to stage 0, 15.5% transition to a lower stage)	G1: 25% improved; 16.6% resolved G2: 66.6% improved in which 33% had complete healing (resolved) G3: 52.9% resolved G4: 88.8% improved	No
				Statistical significance comparing G1 with G2 (*p* = 0.0346), G4: healing improved compared with G1, G2 and G3 *(p* < 0.05); Comparing G1 + G2 vs. G3 + G4 showed complete healing and clinical improvement in all (*p* = 0.0003). No statistical difference in healing between G1 + G3 vs. G2 + G4, but clinical improvement in G4 (*p* = 0.0003); None of the results influenced by site (*p* = 0.28) or underlying diseases (*p* = 0.088). No significant difference in drug holiday protocols observed (*p* = 0.4656). No significant difference between smoking, type of BPs and lesion improvement *p* = 0.9027	BRONJ stage III clinical improvement (*p* = 0.0007)		
[112]	T	Mixed	G1: Med G2: Med + PBM G3: Med + CS G4: SL + PBM	Follow-up timepoints not specified	Nothing mentioned in terms of staging improvement for G1, G2 and G3; G4, transition to stage 0	G1: 0% healed, but NM in reasons and course of action. G2: 41%—Resolved G3: 46%—Resolved G4: 100% improvement, but a relapse of one patient after 10 months—Progressive	One at 10/12
				Statistically significant (*p* < 0.001) in G4 compared to G1. Statistically significant (*p* = 0.03) in G4 compared to G3. G4 had best mucosal healing *p* < 0.0001. Slight clinical improvement difference between CS 46% and PBM alone 41%			
[113]	T	mixed	G1: Med G2: Med + PBM G3: Med + CS G4: Med + CS + PBM G5: Med + SL	Non-oncology: Statistically significant improvement in G1 + G2 compared with G3 + G4 + G5 *p* = 0.0080. Statistically significant in wound healing in G1 + G2 compared with G3 + G4 + G5 *p* = 0.00001. In Oncology: Statistically significant improvement in G1 compared to G2; Statistically significant improvement in G1 + G2 compared with G3 + G4 + G5 *p* < 0.0001. Statistically significant in terms of healing G1 + G2 compared with G3 + G4 + G5 *p* < 0.0001. Statistically significant improvement in G1 + G2 compared with G3 + G4 + G5 *p* = 0.0061; Comparing oncology and non-oncology patients in terms of complete healing for G3 + G4 + G5 showed statistically significant for surgical approach, indicating complete mucosal healing and clinical improvement with surgical treatment at early stages (better results)	Stage I: 75%, Stage II: 54.24% Stage III: 33.3%	Improved: 81.57% sites treated in non-oncology patients and 68.75% in oncology patients. Complete healing: 71.05% sites treated in non-oncology patients and 53% sites in oncology patients. Resolved; Nonsurgical approach adopted on 69 sites induced an improvement in 35 sites and complete healing in 19 sites, while surgical approach performed on 97 sites induced an improvement in 84 sites, of which 78 completely healed.	No
[114]	P	mixed	G1: Med + CS + PBM	Only 15 patients had a delayed healing with minimal bone exposure. NM for the rest of the recruited patients; follow-up, healing time	NM	NM	No

**Table 10:** Representation of MRONJ Staging downscaled after treatment compared to initial staging among the studies employed therapeutic approach.

Study Reference	Therapeutic Protocol	Initial Stage	Downscaling MRONJ Grade and %
[103]	PBM	I:5%; IB: 5%; IIA: 65%; IIB:15%; IIIA: 10%	Stage I + II + III → downstaged → 60%
			Stage I + Stage II → 40%
[104]	Med + CS + PBM	II: 81.81%; III: 18.18%	NM the grade
[105]	Med + SL + PBM Med + CS	I: 30% II: 70%	16.7% Stage I + 71.4% Stage II → Stage 0 83.3% Stage I + 28.6% Stage II → Downscaled (NM)
[106]	Med + CS	I: 8.39%; II 65/131 (49.61%); I II: 55/131 (41.98%)	100% I + Stage II → Stage 0 31/37 (86.48%) Stage III → Stage 0 5/37 (13.51%) Stage III → Stage I
	Med + PBM		1/24 (4.16%) Stage II → Stage I 1/24 (4.16%) Stage III → Stage II 21/24 (87.5%) → Stable 1/24 (4.16%) Stage II → Stage III
[107]	G1a: Med + CS + SL + Piezo + PRP + PBM G1b: Med + CS + PRP + PBM G1c: Med + CS + SL + PRP + PBM G1d: Med + CS + Piezo + PRP + PBM G1e: Med + Piezo + PRP + PBM G1f: Med + SL + PRP + PBM G1g: Med + SL + Piezo + PRP + PBM	I: 9.6%; II: 71.4%; III:19%	I + II + III → 0 (100%)
[109]	Med + CS + Piezo + PRF + PBM	II: 71.42%; III: 8.57%	III → II
[111]	Med	I: 17/128 (13.28%) II: 92/128 (71.87%) III: 19/128 (14.84%)	3/12 (2%) → Stage 0
	Med + PBM		66.6% → downscaled, in which 50% → Stage 0
	Med + CS		9/17 (52.9%) → downscaled,
	Med + CS + LD + PBM		88.8% → downstaged
[112]	G1: Med	I: 8.79% II: 72.52% III: 8.68%	0% → 0
	G2: Med + PBM		41% → Stage 0
	G3: Med + CS		46% → Stage 0
	G4: LS + PBM		83.3% → downstaged
[113]	G1: Med	I: 17.89% II: 66.31% III: 15.78%	Stage I → Stage 0 → 75% Stage II → Stage 0 → 54.2% Stage III → Stage 0 → 33.33%
	G2: Med + PBM		
	G3: Med + CS		
	G4: Med + CS + PBM		
	G5: Med + LS		

**Table 11:** Tabular representation describing the assessment of the clinical parameters used for the selected eligible studies. Abbreviations: Y = yes; N = no; NS = not specified; NI = no information; NSS = not statistically significant; SS = statistically significant.

Study Reference	Pain		Infection		Paraesthesia		Bone Exposure		Oro-Antral Communication		Complete Mucosal Healing		Complete Resolution		No Response		Lesion Recurrence	
	SS Y/N/NS/NI	NSS Y/N/NS/ NI	SS Y/N/ NS/NI	NSS Y/N/ NS/NI	SS Y/N/NS/NI	NSS Y/N/NS/NI	SS Y/N/NS/NI	NSS Y/N/NS/NI	SS Y/N/NS/NI	NSS Y/N/NS/NI	SS Y/N/NS/NI	NSS Y/N/NS/NI	SS Y/N/NS/NI	NSS Y/N/NS/NI	SS Y/N/NS/NI	NSS Y/N/NS /NI	SS Y/N/NS/ NI	NSS Y/N/NS/NI
[103]	Y	N	Y	N	NI	NI	N	Y	NI	NI	Y	N	Y	N	NS	NS	NS	NS
[104]	NI	NI	NI	NI	NI	NI	NI	NI	NI	NI	NI	NI	NI	NI	NI	NI	NI	NI
[105]	NI	NI	NI	NI	NI	NI	NI	NI	NI	NI	N	Y	NI	NI	NI	NI	NI	NI
[106]	NI	NI	NI	NI	NI	NI	NI	NI	NI	NI	NS	NS	NI	NI	NI	NI	NI	NI
[107]	NI	NI	NI	NI	NI	NI	NI	NI	NI	NI	NS	NS	NI	NI	NI	NI	NS	NS
[108]	NI	NI	NI	NI	NI	NI	NI	NI	NI	NI	N	Y	NI	NI	NI	NI	NI	NI
[109]	NI	NI	NI	NI	NI	NI	NI	NI	NI	NI	NS	NS	NI	NI	NI	NI	NI	NI
[110]	NS	NS	NS	NS	NI	NI	NI	NI	NI	NI	NS	NS	NI	NI	NI	NI	NI	NI
[111]	NI	NI	NI	NI	NI	NI	NI	NI	NI	NI	Y	N	NI	NI	NI	NI	NI	NI
[112]	NI	NI	NI	NI	NI	NI	NI	NI	NI	NI	Y	N	NI	NI	NI	NI	NI	NI
[113]	NI	NI	NI	NI	NI	NI	NI	NI	NI	NI	Y	N	Y	N	NI	NI	NI	NI
[114]	NS	NS	NS	NS	NI	NI	NI	NI	NI	NI	NS	NS	NS	NS	NI	NI	NI	NI

**Table 12:** Summary of ROBINS-I tool assessment of included studies.

Domains								Overall Risk of Bias
Study Reference	Pre-Intervention		Intervention	Post-Intervention				
	Bias Due to Confounding	Bias in Selection of Participants for the Study	Bias in Classifying Interventions	Bias Due to Deviations from Intended Interventions	Bias Due to Missing Data	Bias to Measuring Outcomes	Bias in Selecting Reported Results	
[103]	Moderate	Low	Low	Low	Low	Low	Low	Moderate
[104]	Moderate	Low	Low	Low	Low	Moderate	Low	Serious
[105]	Moderate	Low	Low	Low	Low	Moderate	Low	Serious
[106]	Moderate	Low	Low	Low	Low	Moderate	Low	Serious
[107]	Moderate	Low	Low	Low	Low	Moderate	Low	Serious
[108]	Moderate	Low	Low	Low	Low	Moderate	Low	Serious
[109]	Moderate	Low	Low	Low	Low	Moderate	Low	Serious
[110]	Moderate	Low	Low	Low	Low	Moderate	Low	Serious
[111]	Moderate	Low	Low	Low	Low	Moderate	Low	Serious
[112]	Moderate	Low	Low	Low	Low	Moderate	Low	Serious
[113]	Moderate	Low	Low	Low	Low	Moderate	Low	Serious
[114]	Moderate	Low	Low	Low	Low	Moderate	Low	Serious

**Table 13:** Tabular representation of limitations for meta-analytical assessment of the included studies.

Study Reference	Important Characteristics to Consider for a Potential MA
[103]	Therapeutic PBM approach, prospective cohort study, no control group, study groups—only PBMT (diode GaAs 904 nm laser), follow-up duration: 242 d (±38)–8 months, moderate RoB
[104]	Therapeutic PBM approach, retrospective case series, no control group, study groups—PBM (GaAlAs diode 808 nm/laser) + medical/surgical interventions, missing numerical data, follow-up duration: 11.72 m (range between 6 m and 25 m), severe RoB
[105]	Therapeutic PBM approach, retrospective study, study groups—PBMT (1064 nm Nd:YAG laser) + laser surgery vs. conventional surgery, follow-up duration: 13.3 m (range between 3 m and 28 m), severe RoB
[106]	Therapeutic PBM approach, retrospective study, study groups—surgical vs. non-surgical t/t (diode 810 nm/laser), missing numerical data, follow-up duration: 18 m (range 12–28), severe RoB
[107]	Therapeutic PBM approach, prospective study, study groups surgery vs. laser surgery vs. PRP vs. PBMT (diode 808 nm laser), missing numerical data, follow-up duration: 9.6 m (range 2–24 months), severe RoB
[108]	Preventive PBM approach, retrospective study, study groups—antibiotics vs. surgical vs. PRP vs. PBMT (1064 nm Nd:YAG laser), missing numerical data, follow-up duration: 1, 3, 6 m (mean follow-up 14.2 m), severe RoB
[109]	Therapeutic PBM approach, prospective study, study groups—surgical vs. PRP vs. PBMT (1064 nm Nd:YAG laser), missing numerical data, follow-up duration: 18.04 ± 2.14 months, severe RoB
[110]	Preventive PBM approach, prospective study, study groups—Group 1 (G1) included extractions performed in patients previously treated and completely healed for MRONJ in a different site from extraction. Group 2 (G2) included extractions performed in patients previously affected with MRONJ in the same site of extraction, laser parameters (1064 nm Nd:YAG laser), missing numerical data, follow-up duration: 33 m, severe RoB
[111]	Therapeutic PBM approach, retrospective study, study groups—medical or surgical, traditional or laser-assisted approach, with or without PBMT (1064 nm Nd:YAG laser), missing numerical data, follow-up duration: 16 months (range of 6–54 months), severe RoB
[112]	Therapeutic PBM approach, retrospective study, study groups—medical vs. surgical vs. PBMT (1064 nm Nd:YAG laser), missing numerical data, follow-up duration: G1: 4.1 m, G2: 7.5 m, G3: 8.8 m, G4:13 m), severe RoB
[113]	Therapeutic PBM approach, retrospective study, study groups—oncology vs. non-oncology, medical or surgical, traditional or laser-assisted approach, with or without LLLT (1064 nm Nd:YAG laser), missing numerical data, follow-up duration: 16.44 ± 10.95 months, severe RoB
[114]	Preventive PBM approach, case series, study groups—no control group, study groups—only LLLT (1064 nm Nd:YAG laser), follow-up duration: 15 m (4–31 months), severe RoB

**Table 14:** Summary representation of the recruited subjects’ demographic and clinical variables of the recruited subjects of the eligible studies of the present systematic review.

Demographic and Clinical Variable of all the Eligible Studies		% of Data Present	% of NM
Gender		M: 26.07; F: 73.92	NA
Mean age (yrs)		67.52	8.33
Primary disease	Malignant	25	NA
	Mixed (Malignant/ Non-malignant)	75	NA
Type of malignant	Prostate	41.66	8.33
	Breast	41.66	
	Kidney	8.33	
	Others	83.33	
Risk factors	Extraction/MOS	77.77	NA
	Denture wearer	44.44	
	Absence of traumatic factors	22.22	
	Systematic corticosteroids	75	
	DM	50	
Comorbidities	1	33.33	50
	2	0	
	≥3	16.66	
ART/antiangiogenic medications	Zoledronic acid	58.33	NA
	Alendronic acid	25	
	Ibandronic acid	8.33	
	Pamidronate	16.66	
	Denosumab	8.33	
	Sunitinib	16.66	
Route of drug administration	IV	66.66	NA
	PO	41.66	
	IM	8.33	
Length of the administration (months) (mean)		40.24 (in 83.33%)	16.66

**Table 15:** Representation of the oral and dental pathway strategies.

ART Timeframe	Risk	Oral and Dental Care Protocol and Treatment Plan	Reference
Pre/peri/post-ART	At low and high	MDT consultation to evaluate the case. Regular dental check-up at least 4 times a year. A thorough physical and subjective examination. Periodic correction of dental prostheses. Intraoral examination: posterior 2/3 tongue emerges as a valid indicator of the patient’s microbiota status. Initial consultation with X-ray, OPT. DMFT (Decayed, Missed and Filled Teeth). Oral Health-Related Quality of Life. Periodontal disease management. Removal of unrestored teeth/eradicate pathologies in atraumatic approach, at least 3 weeks prior ART for mucosal healing/4–6 weeks prior initiating ART for initial bone healing). Minimal restorative treatment and manual instruments/Er:YAG laser. Mouthwash regimens. Employing PBM for pain management, wound healing and anti-inflammatory effects. aPDT in management of periodontal diseases, viral and bacterial management. Periodic professional oral hygiene. Patient education and motivation and make them aware of the drug complications. Home oral-hygiene protocol.	Table 7 Subheadings 4.5.4. and 4.7.1. [27,200,201,202]

**Table 16:** Representation of authors suggested rationalised consensus and recommendations based on the Level of Evidence of the included studies of the present review and the current the scientific literature for robust and valid methodology for future extensive RCTs.

Key Factors		Suggested Recommendation		Citation of Evidence
**Recruiting subjects and sample size**		Mean age: >60 years old Gender: Same gender cohort in each interventional arm. Mixed-gender subjects can be determined based on the RCTs’ aims and objectives. Sample size: equal distribution of the sample size in the intervention and placebo groups. Sample size calculation should be employed to determine the significant number of participants, serving the study’s endpoints. Subjects with the same systemic disease, primary diagnosis and MRONJ grade. Oncology or non-oncology cohort. Mixed cohort with different BPs intake has a great impact on the management protocol. Subjects with one affected site either; maxilla or mandible in all interventional arms.		Subheadings 4.1 and 4.2, [208] and Table 2 and Table 14
**Randomisation and blinding processes**		Two independent blind investigators to assess the variables at all timepoints. Double-blind and record the data. Robust randomised process. Parallel arm study design.		Subheading 4.3.1 Evaluation of study design
**Risk factors**		Local; systemic; drug-related factors (drug class; bioavailability; administrative route; cumulative drug dose, which is influenced by drug half-shelf-life; and treatment duration). Holiday drug depends on MDT decision. MRONJ staging. Oncology or non-oncology cohort. Identifying patients at risk: detect disease early and treat appropriately based on severity.		Subheadings: 1.1.,1.4, 4.2., 4.4 and 4.3.2 Table 14
**Eligibility criteria**		Currently, AAOMS 2022 is very comprehensive to employ. Identifying subjects at highest risk for MRONJ treated with ART.		[58]
**Study protocol**		Needs for improvement in study methodology. Provision of data related to all aspects of study protocol. Provision of numerical data of treatment outcomes for quantitative analysis.		Subheadings: 3.13, 3.14 and 3.17. Table 2 and Table 9
**Interventional arms**		**Therapeutic**	Main arm: PBM Comparable arms: PBM + aPDT; LS + aPDT + PBM; PBM + CS + aPDT +/− piezo + PRF Standard treatment care/sham PBM arm	Subheading 4.5.4
		**Preventive**	Main arm: PBM Comparable arms: PBM + CS + aPDT; PBM + CS + aPDT Standard treatment care/sham PBM arm	Subheading 4.7.1
**Note: advanced stage III MRONJ**		Invasive surgery (resection) would be the primary treatment modality, and combined PBM and aPDT, as adjunct with or without bone augmentation (APCs or bone graft depending on the size of the defect). MDT consensus is crucial prior to the therapeutic		Subheading 4.5.3
**PBM and aPDT dosimetry and** **treatment protocols** (**therapeutic or preventive)**	**PBM**	WALT suggested dosimetry (oncology and non-oncology cohort) Based on the included studies [104,105], the authors suggested the following laser dosimetry: 808 nm at 5 J/cm^2^ is 7.5 p.J/cm^2^ which is 1.7 Einstein; 1060 nm at 6.5 J/cm^2^ is also 7.5 p.J/cm^2^ which is 1.7 Einstein.		Subheading 4.6[104,105,196]
	**aPDT**	0.01% methylene blue and its derivative, 0.01% phenothiazine chloride were the reported photosensitisers.		[120,209]
**Oral and dental assessment pathways**		Pre-, peri- and post-ART oral and dental care protocols Mouthwash regimens pre/peri/post-ART		Table 7 and Table 15
**Diagnostic criteria/assessment tools for preventive and therapeutic approaches**		OS measurement Oxidised glutathione (GSSG)/glutathione (GSH) ratio is a significant factor in predicting the development of MRONJ. Salivary levels of various biomarkers (hypotaurine, IL1alpha, IL-Beta, IL-1RAA and IL6). CTX, BAP, IL-17, neutrophil function, OS, NTX, metalloproteinase-9 (MMP-9), VEGF, CRP and leukocyte count. BTM for treatment response prediction/control of treatment compliance Imaging: CBCT, CT and MRI. Clinical examinations, clinical photos. Histological analysis (biopsy). Microbiological profile: detecting oral bacterial load; resistance, susceptibility.		Subheadings 1.3.3., 1.3.1. and 4.3.2; Table 8; and [47,89,210]
**CT**		treatment protocol increase MRONJ incidence. Medication type and dose;		Subheadings 1.2. 1.1.1 and 1.4.2; and [38]
**Comorbidity/primary lesion evaluation**		Validated Adult Comorbidity Evaluation (ACE)		Results section and Table 2 and Table 14
**Endpoints**		Quantifiable and clinically relevant.		Subheadings 2.2 and 2.7, Table 14 and [89]
**Response assessment criteria**		Complete healing time; lesion response criteria: resolved; recurrence; stable; complete healing; VAS for pain; Wound-healing grades for mucosal healing		[89]
**Follow-up timepoint**		1/12; 3/12; 6/12; 12/12; 18/12; 24/12 for both therapeutic and preventive approaches for both oncology and non-oncology cohorts. The authors advise the follow-up period to be up to 4 years in advanced MRONJ (stage III) and in oncology cases. MDT needs is the decision maker		Table 4 and Table 14

## Data Availability

All the data are presented in the text.

## Supplementary Materials

The following supporting information can be downloaded at: https://www.mdpi.com/article/10.3390/ph17081011/s1, Supplementary file 1 (S1): PRISMA 2020 Checklist.

## References

[1] Khan A.A., Morrison A., Kendler D.L., Rizzoli R., Hanley D.A., Felsenberg D., McCauley L.K., O’Ryan F., Reid I.R., Ruggiero S.L. (2017). Case-Based Review of Osteonecrosis of the Jaw (ONJ) and Application of the international recommendations for management from the international task force on ONJ. J. Clin. Densitom..

[2] King R., Tanna N., Patel V. (2019). Medication-related osteonecrosis of the jaw unrelated to bisphosphonates and denosumab—A review. Oral Surg. Oral Med. Oral Pathol. Oral Radiol..

[3] Shibahara T. (2019). Antiresorptive agent-related osteonecrosis of the jaw (ARONJ): A twist of fate in the bone. Tohoku J. Exp. Med..

[4] Eguia A., Bagán-Debón L., Cardona F. (2020). Review and update on drugs related to the development of osteonecrosis of the jaw. Med. Oral Patol. Oral Cir. Bucal.

[5] Santos-Silva A.R., Belizario Rosa G.A., Castro Junior G., Dias R.B., Prado Ribeiro A.C., Brandao T.B. (2013). Osteonecrosis of the mandible associated with bevacizumab therapy. Oral Surg. Oral Med. Oral Pathol. Oral Radiol..

[6] Lombard T., Neirinchx V., Rogister B., Gilon Y., Wislet S. (2016). Medications-Related Osteonecrosis of the Jaw: New Insights into Molecular Mechanisms and Cellular Therapeutic Approaches. Stem Cells Int..

[7] Yuan F., Peng W., Yang C., Zheng J. (2019). Teriparatide versus bisphosphonates for treatment of postmenopausal osteoporosis: A meta-analysis. Int. J. Surg..

[8] Bartl R., Frisch B., von Tresckow E., Bartl C. (2007). Bisphosphonates in Medical Practice Actions, Side Effects, Indication, Strategies.

[9] Diel I.J., Bergner R., Grötz K.A. (2007). Adverse effects of bisphosphonates: Current issues. J. Support. Oncol..

[10] Shkolnikova J., Flynn J., Choong P. (2013). Burden of bisphosphonate-associated femoral fractures. ANZ J. Surg..

[11] Orozco C., Maalouf N.M. (2012). Safety of bisphosphonates. Rheum. Dis. Clin. N. Am..

[12] Lasseter K.C., Porras A.G., Denker A., Santhanagopal A., Daifotis A. (2005). Pharmacokinetic considerations in determining the terminal elimination half-lives of bisphosphonates. Clin. Drug Investig..

[13] Walter C., Klein M.O., Pabst A., Al-Nawas B., Duschner H., Ziebart T. (2010). Influence of bisphosphonates on endothelial cells, fibroblasts, and osteogenic cells. Clin. Oral Investig..

[14] Hampson G., Fogelman I. (2012). Clinical role of bisphosphonate therapy. Int. J. Women’s Health.

[15] Abdelmoula L.C., Ben M’barek R., Ben Hadj Yahia C., Tekaya R., Testouri N., Chaabouni L., Zouari R. (2011). Indications des bisphosphonates dans les affections osseuses autres que l’ostéoporose [Bisphosphonates: Indications in bone diseases other than osteoporosis]. Tunis. Med..

[16] Otto S., Pautke C., Van den Wyngaert T., Niepel D., Schiødt M. (2018). Medication-related osteonecrosis of the jaw: Prevention, diagnosis and management in patients with cancer and bone metastases. Cancer Treat. Rev..

[17] Ferreira L.-H., Mendonça K.D., Chaves de Souza J., Soares Dos Reis D.C., do Carmo Faleiros Veloso Guedes C., de Souza Castro Filice L., Bruzadelli Macedo S., Soares Rocha F. (2021). Bisphosphonate-associated osteonecrosis of the jaw. Minerva Dent. Oral Sci..

[18] Zavras A.I. (2011). The impact of bisphosphonates on oral health: Lessons from the past and opportunities for the future. Ann. N. Y. Acad. Sci..

[19] Berenson J.R., Hillner B.E., Kyle R.A., Anderson K., Lipton A., Yee G.C., Biermann J.S. (2002). American Society of Clinical Oncology clinical practice guidelines: The role of bisphosphonates in multiple myeloma. J. Clin. Oncol..

[20] Cremers S., Ebetino F.H., Phipps R. (2020). On the pharmacological evaluation of bisphosphonates in humans. Bone.

[21] Qi W.X., Tang L.N., He A.N., Yao Y., Shen Z. (2014). Risk of osteonecrosis of the jaw in cancer patients receiving denosumab: A meta-analysis of seven randomized controlled trials. Int. J. Clin. Oncol..

[22] Cummings S.R., San Martin J., McClung M.R., Siris E.S., Eastell R., Reid I.R., Delmas P., Zoog H.B., Austin M., Wang A. (2009). Denosumab for prevention of fractures in postmenopausal women with osteoporosis. N. Engl. J. Med..

[23] Thumbigere-Math V., Tu L., Huckabay S., Dudek A.Z., Lunos S., Basi D.L., Hughes P.J., Leach J.W., Swenson K.K., Gopalakrishnan R. (2012). A retrospective study evaluating frequency and risk factors of osteonecrosis of the jaw in 576 cancer patients receiving intravenous bisphosphonates. Am. J. Clin. Oncol..

[24] Van den Wyngaert T., Claeys T., Huizing M.T., Vermorken J.B., Fossion E. (2009). Initial experience with conservative treatment in cancer patients with osteonecrosis of the jaw [ONJ] and predictors of outcome. Ann. Oncol..

[25] Henry D.H., Costa L., Goldwasser F., Hirsh V., Hungria V., Prausova J., Scagliotti G.V., Sleeboom H., Spencer A., Vadhan-Raj S. (2011). Randomized, double-blind study of denosumab versus zoledronic acid in the treatment of bone metastases in patients with advanced cancer [excluding breast and prostate cancer] or multiple myeloma. J. Clin. Oncol..

[26] Khosla S., Burr D., Cauley J., Dempster D.W., Ebeling P.R., Felsenberg D., Gagel R.F., Gilsanz V., Guise T., Koka S. (2007). Bisphosphonate-associated osteonecrosis of the jaw: Report of a task force American Society for Bone and Mineral Research. J. Bone Miner. Res..

[27] Ruggiero S.L., Dodson T.B., Fantasia J., Goodday R., Aghaloo T., Mehrotra B., O’Ryan F. (2014). American Association of Oral Maxillofacial Surgeons position paper on medication-related osteonecrosis of the jaw-2014 update. J. Oral Maxillofac. Surg..

[28] Then C., Hörauf N., Otto S., Pautke C., von Tresckow E., Röhnisch T., Baumann P., Schmidmaier R., Bumeder I., Oduncu F.S. (2012). Incidence and risk factors of bisphosphonate related osteonecrosis of the jaw in multiple myeloma patients having undergone autologous stem cell transplantation. Onkologie.

[29] Lo J.C., O’Ryan F.S., Gordon N.P., Yang J., Hui R.L., Martin D., Hutchinson M., Lathon P.V., Sanchez G., Silver P. (2010). Prevalence of osteonecrosis of the jaw in patients with oral bisphosphonate exposure. J. Oral Maxillofac. Surg..

[30] Jadu F., Lee L., Pharoah M., Reece D., Wang L. (2007). A retrospective study assessing the incidence, risk factors and comorbidities of pamidronate-related necrosis of the jaws in multiple myeloma patients. Ann. Oncol..

[31] Bittrich M., Hetterich R., Solimando A.G., Krebs M., Loda S., Danhof S., Anton S., Zhou X., Kerscher A., Beilhack A. (2023). Does medication-related osteonecrosis of the jaw affect survival of patients with Multiple Myeloma? Exploring a large single center database using artificial intelligence. Clin. Exp. Med..

[32] Hoff A.O., Toth B.B., Altudag K., Johnson M.M., Warneke C.L., Hu M., Nooka A., Sayegh G., Guarneri V., Desrouleaux K. (2008). Frequency and risk factors associated with osteonecrosis of the jaw in cancer patients related with intravenous bisphosphonates. J. Bone Miner. Res..

[33] Fehm T., Beck V., Banys M., Lipp H.P., Hairass M., Reinert S., Solomayer E., Wallwiener D., Krimmel M. (2009). Bisphosphonate-induced osteonecrosis of the jaw [ONJ]: Incidence and risk factors in patients with breast cancer and gynecological malignancies. Gynecol. Oncol..

[34] Assaf A.T., Smeets R., Riecke B., Weise E., Gröbe A., Blessmann M., Steiner T., Wikner J., Friedrich R.E., Heiland M. (2013). Incidence of bisphosphonate-related osteonecrosis of the jaw in consideration of primary diseases and concomitant therapies. Anticancer. Res..

[35] Troeltzsch M., Woodlock T., Kriegelstein S., Steiner T., Messlinger K. (2012). Physiology and pharmacology of nonbisphosphonate drugs implicated in osteonecrosis of the jaw. J. Can. Dent. Assoc..

[36] Ortega J., Vigil C.E., Chodkiewicz C. (2010). Current progress in targeted therapy for colorectal cancer. Cancer Control.

[37] Schmid T.A., Gore M.E. (2016). Sunitinib in the treatment of metastatic renal cell carcinoma. Ther. Adv. Urol..

[38] Estilo C.L., Fornier M., Farooki A., Carlson D., Bohle G., Huryn J.M. (2008). Osteonecrosis of the jaw related to bevacizumab. J. Clin. Oncol..

[39] Wimalawansa S.J. (2008). Insight into bisphosphonate-associated osteomyelitis of the jaw: Pathophysiology, mechanisms and clinical management. Expert Opin. Drug Saf..

[40] Marx R.E., Sawstari Y., Fortin M., Broumand V. (2005). Bisphosphonate-induced exposed bone [osteonecrosis/osteoporosis] of the jaws: Risk factors, recognition, prevention, and treatment. J. Oral Maxillofac. Surg..

[41] Sharma D., Hamlet S., Petcu E., Ivanovski S. (2016). The effect of bisphosphonates on the endothelial differentiation of mesenchymal stem cells. Sci. Rep..

[42] Walter C., Pabst A., Ziebart T., Klein M., Al-Nawas B. (2011). Bisphosphonates affect migration ability and cell viability of HUVEC, fibroblasts and osteoblasts in vitro. Oral Dis..

[43] Tamaoka J., Takaoka K., Hattori H., Ueta M., Maeda H., Yamamura M., Yamanegi K., Noguchi K., Kishimoto H. (2019). Osteonecrosis of the jaws caused by bisphosphonate treatment and oxidative stress in mice. Exp. Ther. Med..

[44] Hansen T., Kunkel M., Springer E., Walter C., Weber A., Siegel E., Kirkpatrick C.J. (2007). Actinomycosis of the jaws-histopathological study of 45 patients shows significant involvement in bisphosphonate-associated osteonecrosis and infected osteoradionecrosis. Virchows Arch..

[45] Landesberg R., Woo V., Cermers S., Cozin M., Marolt D., Vunjak-Novakovic G., Kousteni S., Raghavan S. (2011). Potential pathophysiological mechanisms in osteonecrosis of the jaw. Ann. N. Y. Acad. Sci..

[46] Yoneda T., Hagino H., Sugimoto T., Ohta H., Takahashi S., Soen S., Taguchi A., Toyosawa S., Nagata T., Urade M. (2010). Bisphosphonate-related osteonecrosis of jaw: Position paper form the Allied Task Force Committee of Japanese Society for Bone and Mineral Research, Japan Osteoporosis Society, Japanese Society of Periodontology, Japanese Society of Oral and Maxillofacial Radiology, and Japanese Society of Oral and Maxillofacial Surgeons. J. Bone Miner. Metab..

[47] Bagan J., Sáez G.T., Tormos M.C., Gavalda-Esteve C., Bagan L., Leopoldo-Rodado M., Calvo J., Camps C. (2014). Oxidative stress in bisphosphonate-related osteonecrosis of the jaws. J. Oral Pathol. Med..

[48] Papadopoulou E., Nicolatou-Galitis O., Papassotiriou I., Linardou H., Karagianni A., Tsixlakis K., Tarampikou A., Michalakakou K., Vardas E., Bafaloukos D. (2020). The use of crevicular fluid to assess markers of inflammation and angiogenesis, IL-17 and VEGF, in patients with solid tumors receiving zoledronic acid and/or bevacizumab. Support. Care Cancer.

[49] Marx R.E., Cillo J.E., Ulloa J.J. (2007). Oral bisphosphonate-induced osteonecrosis: Risk factors, prediction of risk using serum CTX testing prevention, and treatment. J. Oral Maxillofac. Surg..

[50] Aghaloo T.L., Kang B., Sung E.C., Shoff M., Ronconi M., Gotcher J.E., Bezouglaia O., Dry S.M., Tetradis S. (2011). Periodontal disease and bisphosphonates induced osteonecrosis of the jaws in the rat. J. Bone Min. Res..

[51] Otto S., Aljohani S., Fliefel R., Ecke S., Ristow O., Burian E., Troeltzsch M., Pautke C., Ehrenfeld M. (2021). Infection as an important factor in medication-related osteonecrosis of the jaw (MRONJ). Medicina.

[52] Neviaser A.S., Lane J.M., Lenart B.A., Edobor-Osula F., Lorich D.G. (2008). Low-energy femoral shaft fractures associated with alendronate use. J. Orthop. Trauma.

[53] O’Ryan F.S., Lo J.C. (2012). Bisphosphonate-related osteonecrosis of the jaw in patients with oral bisphosphonate exposure: Clinical course and outcomes. J. Oral Maxillofac. Surg..

[54] Di Fede O., Fusco V., Matranga D., Solazzo L., Gabriele M., Gaeta G.M., Favia G., Sprini D., Peluso F., Colella G. (2013). Osteonecrosis of the jaws in patients assuming oral bisphosphonates for osteoporosis: A retrospective multihospital-based study of 87 Italian cases. Eur. J. Intern. Med..

[55] Malden N., Lopes V. (2012). An epidemiological study of alendronate-related osteonecrosis of the jaws. A case series from the south-east of Scotland with attention given to case definition and prevalence. J. Bone Miner. Metab..

[56] Diniz-Freitas M., Lopez-Cendrun J.L., Fernandez-Sanroman J., Garcia-Garcia A., FernandezFeijoo J., Diz-Dios P. (2012). Oral bisphosphonate- related osteonecrosis of the jaws: Clinical charasteristics of a series of 20 cases in Spain. Med. Oral Patol. Oral Cir. Bucal..

[57] Zhong D.N., Wu J.Z., Li G.J. (2013). Association between CYP2C8 [rs1934951] polymorphism and bisphosphonate-related osteonecrosis of the jaws in patients on bisphosphonate therapy: A meta-analysis. Acta Haematol..

[58] Ruggiero S.L., Dodson T.B., Aghaloo T., Carlson E.R., Ward B.B., Kademani D. (2022). American Association of Oral and Maxillofacial Surgeons’ Position Paper on Medication-Related Osteonecrosis of the Jaws-2022 Update. J. Oral Maxillofac. Surg..

[59] On S.W., Cho S.W., Byun S.H., Yang B.E. (2021). Various Therapeutic Methods for the Treatment of Medication-Related Osteonecrosis of the Jaw (MRONJ) and Their Limitations: A Narrative Review on New Molecular and Cellular Therapeutic Approaches. Antioxidants.

[60] Hanna R., Bensadoun R.J., Beken S.V., Burton P., Carroll J., Benedicenti S. (2022). Outpatient Oral Neuropathic Pain Management with Photobiomodulation Therapy: A Prospective Analgesic Pharmacotherapy-ParalleledFeasibility Trial. Antioxidants.

[61] Hanna R., Dalvi S., Bensadoun R.J., Raber-Durlacher J.E., Benedicenti S. (2021). Role of Photobiomodulation Therapy in Neurological Primary Burning Mouth Syndrome. A Systematic Review and Meta-Analysis of Human Randomised Controlled Clinical Trials. Pharmaceutics.

[62] Hanna R., Dalvi S., Bensadoun R.J., Benedicenti S. (2021). Role of Photobiomodulation Therapy in Modulating Oxidative Stress in Temporomandibular Disorders. A Systematic Review and Meta-Analysis of Human Randomised Controlled Trials. Antioxidants.

[63] Hanna R., Dalvi S., Tomov G., Hopper C., Rebaudi F., Rebaudi A.L., Bensadoun J.R. (2023). Emerging potential of phototherapy in management of symptomatic oral lichen planus: A systematic review of randomised controlled clinical trials. J. Biophotonics.

[64] Hanna R., Dalvi S., Benedicenti S., Amaroli A., Sălăgean T., Pop I.D., Todea D., Bordea I.R. (2020). Photobiomodulation Therapy in Oral Mucositis and Potentially Malignant Oral Lesions: A Therapy Towards the Future. Cancers.

[65] Hanna R., Agas D., Benedicenti S., Ferrando S., Laus F., Cuteri V., Lacava G., Sabbieti M.G., Amaroli A. (2019). A Comparative Study Between the Effectiveness of 980 nm Photobiomodulation Delivered by Hand-Piece with Gaussian vs. Flat-Top Profiles on Osteoblasts Maturation. Front. Endocrinol..

[66] Hanna R., Dalvi S., Amaroli A., De Angelis N., Benedicenti S. (2021). Effects of photobiomodulation on bone defects grafted with bone substitutes: A systematic review of in vivo animal studies. J. Biophotonics.

[67] Abdolrahmani A., Epstein J.B., Samim F. (2024). Medication-related osteonecrosis of the jaw: Evolving research for multimodality medical management. Support. Care Cancer.

[68] Favia G., Tempesta A., Limongelli L., Crincoli V., Maiorano E. (2016). Medication-Related Osteonecrosis of the Jaws: Considerations on a New Antiresorptive Therapy (Denosumab) and Treatment Outcome after a 13-Year Experience. Int. J. Dent..

[69] Albanese M., Zotti F., Capocasale G., Bonetti S., Lonardi F., Nocini P.F. (2020). Conservative non-surgical management in medication related osteonecrosis of the jaw: A retrospective study. Clin. Exp. Dent Res..

[70] Kaibuchi N., Hoshi K., Yamazaki A., Miyamoto-Sangu N., Akagi Y., Okamoto T. (2021). The Progress of Medication-Related Osteonecrosis of the Jaw with Conservative Initial Treatment: A 12-Year Retrospective Study of 129 Patients. Bone Rep..

[71] Osaka R., Kato H., Hamada Y., Fujimoto Y., Mizusawa N., Watanabe D., Kaneko A. (2021). Clinicostatistical Analyses of Medication-Related Osteonecrosis of the Jaws (MRONJ): Evaluation of the Treatment Method and Prognosis. Oral Sci. Int..

[72] Pavlíková G., Foltán R., Horká M., Hanzelka T., Borunska H., Sedy J. (2011). Piezosurgery in oral and maxillofacial surgery. Int. J. Oral Maxillofac. Surg..

[73] Gera I., Szücs N. (2023). The recombinant human parathyroid hormone, teriparatide as an alternative remedy for the medication-related osteonecrosis of the jaw. Orv. Hetil..

[74] Jung J., Shim G.J., Kim M., Yoon Y., Kim J.E., Jue S.S., Al-Nawas B., Kwon Y.D. (2021). Effect and timing of parathyroid hormone analog administration for preventing medication-related osteonecrosis of the jaws in a murine model. J. Craniomaxillofacial Surg..

[75] Sarkarat F., Kalantar Motamedi M.H., Jahanbani J., Sepehri D., Kahali R., Nematollahi Z. (2014). Platelet-Rich Plasma in Treatment of Zoledronic Acid-Induced Bisphosphonate-Related Osteonecrosis of the Jaws. Trauma. Mon..

[76] Sohn D.-S., Heo J.-U., Kwak D.-H., Kim D.-E., Kim J.-M., Moon J.-W., Lee J.-H., Park I.-S. (2011). Bone Regeneration in the Maxillary Sinus Using an Autologous Fibrin-Rich Block with Concentrated Growth Factors Alone. Implant. Dent..

[77] Borsani E., Bonazza V., Buffoli B., Nocini P.F., Albanese M., Zotti F., Inchingolo F., Rezzani R., Rodella L.F. (2018). Beneficial Effects of Concentrated Growth Factors and Resveratrol on Human Osteoblasts In Vitro Treated with Bisphosphonates. Biomed Res. Int..

[78] Dohan Ehrenfest D.M., Bielecki T., Jimbo R., Barbé G., Del Corso M., Inchingolo F., Sammartino G. (2012). Do the Fibrin Architecture and Leukocyte Content Influence the Growth Factor Release of Platelet Concentrates? An Evidence-Based Answer Comparing a Pure Platelet-Rich Plasma (P-PRP) Gel and a Leukocyte- and Platelet-Rich Fibrin (L-PRF). Curr. Pharm. Biotechnol..

[79] Mijiritsky E., Assaf H.D., Kolerman R., Mangani L., Ivanova V., Zlatev S. (2022). Autologous Platelet Concentrates (APCs) for Hard Tissue Regeneration in Oral Implantology, Sinus Floor Elevation, Peri-Implantitis, Socket Preservation, and Medication-Related Osteonecrosis of the Jaw (MRONJ): A Literature Review. Biology.

[80] Wisniewska L.M., Dohan Ehrenfest D.M., Galindo-Moreno P., Segovia J.D., Inchingolo F., Wang H.-L., Fernandes-Cruz M. (2017). Molecular, Cellular and Pharmaceutical Aspects of Biomaterials in Dentistry and Oral and Maxillofacial Surgery. An Internationalization of Higher Education and Research Perspective. Curr. Pharm. Biotechnol..

[81] Del Corso M., Vervelle A., Simonpieri A., Jimbo R., Inchingolo F., Sammartino G., Dohan Ehrenfest D.M. (2012). Current Knowledge and Perspectives for the Use of Platelet-Rich Plasma (PRP) and Platelet-Rich Fibrin (PRF) in Oral and Maxillofacial Surgery Part 1: Periodontal and Dentoalveolar Surgery. Curr. Pharm. Biotechnol..

[82] Bonazza V., Borsani E., Buffoli B., Parolini S., Inchingolo F., Rezzani R., Rodella L.F. (2018). In Vitro Treatment with Concentrated Growth Factors (CGF) and Sodium Orthosilicate Positively Affects Cell Renewal in Three Different Human Cell Lines. Cell Biol. Int..

[83] Borsani E., Buffoli B., Bonazza V., Brunelli G., Monini L., Inchingolo F., Ballini A., Rezzani R., Rodella L.F. (2020). In Vitro Effects of Concentrated Growth Factors (CGF) on Human SH-SY5Y Neuronal Cells. Eur. Rev. Med. Pharm. Sci..

[84] Rusilas H., Balčiūnaitė A., Žilinskas J. (2020). Autologous Platelet Concentrates in Treatment of Medication Related Osteonecrosis of the Jaw. Stomatologija.

[85] Aljohani S., Fliefel R., Ihbe J., Kühnisch J., Ehrenfeld M., Otto S. (2017). What Is the Effect of Anti-Resorptive Drugs (ARDs) on the Development of Medication-Related Osteonecrosis of the Jaw (MRONJ) in Osteoporosis Patients: A Systematic Review. J. Craniomaxillofacal Surg..

[86] Simonpieri A., Del Corso M., Vervelle A., Jimbo R., Inchingolo F., Sammartino G., Dohan Ehrenfest D.M. (2012). Current Knowledge and Perspectives for the Use of Platelet-Rich Plasma (PRP) and Platelet-Rich Fibrin (PRF) in Oral and Maxillofacial Surgery Part 2: Bone Graft, Implant and Reconstructive Surgery. Curr. Pharm. Biotechnol..

[87] Giudice A., Barone S., Giudice C., Bennardo F., Fortunato L. (2018). Can Platelet-Rich Fibrin Improve Healing after Surgical Treatment of Medication-Related Osteonecrosis of the Jaw? A Pilot Study. Oral Surg. Oral Med. Oral Pathol. Oral Radiol..

[88] Asaka T., Ohga N., Yamazaki Y., Sato J., Satoh C., Kitagawa Y. (2017). Platelet-Rich Fibrin May Reduce the Risk of Delayed Recovery in Tooth-Extracted Patients Undergoing Oral Bisphosphonate Therapy: A Trial Study. Clin. Oral Investig..

[89] Yarom N., Shapiro C.L., Peterson D.E., Van Poznak C.H., Bohlke K., Ruggiero S.L., Migliorati C.A., Khan A., Morrison A., Anderson H. (2019). Medication-Related Osteonecrosis of the Jaw: MASCC/ISOO/ASCO Clinical Practice Guideline. J. Clin. Oncol..

[90] Hepburn J., Williams-Lockhart S., Bensadoun R.J., Hanna R. (2022). A Novel Approach of Combining Methylene Blue Photodynamic Inactivation, Photobiomodulation and Oral Ingested Methylene Blue in COVID-19 Management: A Pilot Clinical Study with 12-Month Follow-Up. Antioxidants.

[91] Hamblin M.R. (2017). Mechanisms and applications of the anti-inflammatory effects of photobiomodulation. AIMS Biophys..

[92] Kuffler D.P. (2016). Photobiomodulation in promoting wound healing: A review. Regen. Med..

[93] Dompe C., Moncrieff L., Matys J., Grzech-Lesniak K., Kocherova I., Bryja A., Bruska M., Dominiak M., Mozdziak P., Skiba T.H.I. (2020). Photobiomodulation-underlying mechanism and clinical applications. J. Clin. Med..

[94] de Freitas L.F., Hamblin M.R. (2016). Proposed Mechanism of Photobiomodulation or Low-Level Light. Ther. IEEE J. Sel. Top. Quantum Electron..

[95] McHugh M.L. (2012). Interrater reliability: The kappa statistic. Biochem. Med..

[96] Sterne J.A.C., Hernán M.A., Reeves B.C., Savović J., Berkman N.D., Viswanathan M., Henry D., Altman D.G., Ansari M.T., Boutron I. (2016). ROBINS-I: A tool for assessing risk of bias in non-randomised studies of interventions. BMJ.

[97] Sterne J.A.C., Higgins J.P.T., Elbers R.G., Reeves B.C., the Development Group for ROBINS-I Risk of Bias in Non-randomized Studies of Interventions (ROBINS-I): Detailed Guidance, Updated 12 October 2016. http://www.riskofbias.info.

[98] The Cochrane Collaboration (2020). Review Manager (RevMan) [Computer Program]. Version 5.4.1.

[99] Lau J., Ioannidis J.P., Schmid C.H. (1997). Quantitative synthesis in systematic reviews. Ann. Intern. Med..

[100] Higgins J.P.T., Thompson S.G. (2002). Quantifying heterogeneity in a meta-analysis. Stat. Med..

[101] Sterne J.A.C., Egger M. (2001). Funnel plots for detecting bias in meta-analysis: Guidelines on choice of axis. J. Clin. Epidemiol..

[102] Lin L., Chu H. (2018). Quantifying publication bias in meta-analysis. Biometrics.

[103] Scoletta M., Arduino P.G., Reggio L., Dalmasso P., Mozzati M. (2010). Effect of low-level laser irradiation on bisphosphonate-induced osteonecrosis of the jaws: Preliminary results of a prospective study. Photomed. Laser Surg..

[104] Altay M.A., Tasar F., Tosun E., Kan B. (2014). Low-level laser therapy supported surgical treatment of bisphosphonate related osteonecrosis of jaws: A retrospective analysis of 11 cases. Photomed. Laser Surg..

[105] Atalay B., Yalcin S., Emes Y., Aktas I., Aybar B., Issever H., Mandel N.M., Cetin O., Oncu B. (2011). Bisphosphonate-related osteonecrosis: Laser-assisted surgical treatment or conventional surgery?. Lasers Med. Sci..

[106] Favia G., Tempesta A., Limongelli L., Crincoli V., Maiorano E. (2018). Medication-related osteonecrosis of the jaw: Surgical or nonsurgical treatment?. Oral Dis..

[107] Merigo E., Cella L., Oppici A., Cristina Arbasi M., Clini F., Fontana M., Fornaini C. (2018). Combined Approach to Treat Medication-Related Osteonecrosis of the Jaws. J. Lasers Med. Sci..

[108] Şahin O., Tatar B., Ekmekcioğlu C., Aliyev T., Odabaşı O. (2020). Prevention of medication related osteonecrosis of the jaw after dentoalveolar surgery: An institution’s experience. J. Clin. Exp. Dent..

[109] Şahin O., Akan E., Tatar B., Ekmekcioğlu C., Ünal N., Odabaşı O. (2022). Combined approach to treatment of advanced stages of medication-related osteonecrosis of the jaw patients. Braz. J. Otorhinolaryngol..

[110] Vescovi P., Giovannacci I., Merigo E., Meleti M., Manfredi M., Fornaini C., Nammour S. (2015). Tooth extractions in high-risk patients under bisphosphonate therapy and previously affected with osteonecrosis of the jaws: Surgical protocol supported by low-level laser therapy. J. Craniofacial Surg..

[111] Vescovi P., Manfredi M., Merigo E., Guidotti R., Meleti M., Pedrazzi G., Fornaini C., Bonanini M., Ferri T., Nammour S. (2012). Early surgical laser-assisted management of bisphosphonate-related osteonecrosis of the jaws (BRONJ): A retrospective analysis of 101 treated sites with long-term follow-up. Photomed. Laser Surg..

[112] Vescovi P., Manfredi M., Merigo E., Meleti M., Fornaini C., Rocca J.P., Nammour S. (2010). Surgical approach with Er:YAG laser on osteonecrosis of the jaws (ONJ) in patients under bisphosphonate therapy (BPT). Lasers Med. Sci..

[113] Vescovi P., Merigo E., Meleti M., Manfredi M., Fornaini C., Nammour S. (2012). Surgical Approach and Laser Applications in BRONJ Osteoporotic and Cancer Patients. J. Osteoporos..

[114] Vescovi P., Meleti M., Merigo E., Manfredi M., Fornaini C., Guidotti R., Nammour S. (2013). Case series of 589 tooth extractions in patients under bisphosphonates therapy. Proposal of a clinical protocol supported by Nd:YAG low-level laser therapy. Med. Oral Patol. Oral Cir. Bucal.

[115] Tenore G., Zimbalatti A., Rocchetti F., Graniero F., Gaglioti D., Mohsen A., Caputo M., Lollobrigida M., Lamazza L., De Biase A. (2020). Management of Medication-Related Osteonecrosis of the Jaw (MRONJ) Using Leukocyte- and Platelet-Rich Fibrin (L-PRF) and Photobiomodulation: A Retrospective Study. J. Clin. Med..

[116] Manfredi M., Merigo E., Guidotti R., Meleti M., Vescovi P. (2011). Bisphosphonate-related osteonecrosis of the jaws: A case series of 25 patients affected by osteoporosis. Int. J. Oral Maxillofac. Surg..

[117] Martins M.A., Martins M.D., Lascala C.A., Curi M.M., Migliorati C.A., Tenis C.A., Marques M.M. (2012). Association of laser phototherapy with PRP improves healing of bisphosphonate-related osteonecrosis of the jaws in cancer patients: A preliminary study. Oral Oncol..

[118] Vescovi P., Merigo E., Meleti M., Fornaini C., Nammour S., Manfredi M. (2007). Nd:YAG laser biostimulation of bisphosphonate-associated necrosis of the jawbone with and without surgical treatment. Br. J. Oral Maxillofac. Surg..

[119] Vescovi P., Merigo E., Manfredi M., Meleti M., Fornaini C., Bonanini M., Rocca J.P., Nammour S. (2008). Nd:YAG laser biostimulation in the treatment of bisphosphonate-associated osteonecrosis of the jaw: Clinical experience in 28 cases. Photomed. Laser Surg..

[120] Poli P.P., Ávila Souza F., Susanna Ferrario S., Carlo Maiorana C. (2019). Adjunctive application of antimicrobial photodynamic therapy in the prevention of medication-related osteonecrosis of the jaw following dentoalveolar surgery: A case series. Photodiagnosis Photodyn. Ther..

[121] Tartaroti N.C., Marques M.M., Naclério-Homem M.D.G., Migliorati C.A., Zindel Deboni M.C. (2020). Antimicrobial photodynamic and photobiomodulation adjuvant therapies for prevention and treatment of medication-related osteonecrosis of the jaws: Case series and long-term follow-up. Photodiagnosis Photodyn. Ther..

[122] Erovigni F.M., Cabras M., Gambino A., Todaro D., Carcieri P., Dell’Acqua A. (2021). Photobiomodulation vs. photodynamic therapy as adjuvant treatments in patients with medication-related osteonecrosis of the jaw (mronj): A pilot study. Qeios.

[123] Vescovi P., Merigo E., Meleti M., Manfredi M., Fornaini C., Nammour S., Mergoni G., Sarraj A., Bagan J.V. (2014). Conservative surgical management of stage I bisphosphonate-related osteonecrosis of the jaw. Int. J. Dent..

[124] Angiero F., Sannino C., Borloni R., Crippa R., Benedicenti S., Romanos G.E. (2009). Osteonecrosis of the jaws caused by bisphosphonates: Evaluation of a new therapeutic approach using the Er:YAG laser. Lasers Med. Sci..

[125] Nica D.F., Riviș M., Roi C.I., Todea C.D., Duma V.-F., Sinescu C. (2021). Complementarity of Photo-Biomodulation, Surgical Treatment, and Antibiotherapy for Medication-Related Osteonecrosis of the Jaws (MRONJ). Medicina.

[126] Merigo E., Manfredi M., Meleti M., Guidotti R., Ripasarti A., Zanzucchi E., D’Aleo P., Corradi D., Corcione L., Sesenna E. (2006). Bone necrosis of the jaws associated with bisphosphonate treatment: A report of twenty-nine cases. Acta Biomed..

[127] Vescovi P., Merigo E., Meleti M., Manfredi M., Guidotti R., Nammour S. (2012). Bisphosphonates-related osteonecrosis of the jaws: A concise review of the literature and a report of a single-centre experience with 151 patients. J. Oral Pathol. Med..

[128] Ribeiro G.H., Minamisako M.C., Rath I.B.d.S., Santos A.M.B., Simões A., Pereira K.C.R., Grando L.J. (2018). Osteoradionecrosis of the jaws: Case series treated with adjuvant low-level laser therapy and antimicrobial photodynamic therapy. J. Appl. Oral Sci..

[129] Page M.J., McKenzie J.E., Bossuyt P.M., Boutron I., Hoffmann T.C., Mulrow C.D., Shamseer L., Tetzlaff J.M., Akl E.A., Brennan S.E. (2021). The PRISMA 2020 statement: An updated guideline for reporting systematic reviews. BMJ.

[130] Dioguardi M., Spirito F., Alovisi M., Aiuto R., Garcovich D., Crincoli V., Ballini A., Caloro G.A., Muzio L., Ballini A. (2023). Location and Gender Differences in Osteonecrosis of the Jaws in Patients Treated with Antiresorptive and Antineoplastic Drugs Undergoing Dentoalveolar Surgical, Systematic Review with Meta-Analysis and Trial Sequential Analysis. J. Clin. Med..

[131] Gaudin E., Seidel L., Bacevic M., Rompen E., Lambert F. (2015). Occurrence and risk indicators of medication-related osteonecrosis of the jaw after dental extraction: A systematic review and meta-analysis. J. Clin. Periodontol..

[132] Srivastava A., Nogueras Gonzalez G.M., Geng Y., Won A.M., Myers J., Li Y., Chambers M.S. (2021). Medication-Related Osteonecrosis of the Jaw in Patients Treated Concurrently with Antiresorptive and Antiangiogenic Agents: Systematic Review and Meta-Analysis. J. Immunother. Precis. Oncol..

[133] Momesso G.A.C., Lemos C.A.A., Santiago-Júnior J.F., Faverani L.P., Pellizzer E.P. (2020). Laser surgery in management of medication-related osteonecrosis of the jaws: A meta-analysis. Oral Maxillofac. Surg..

[134] Yarom N., Lazarovici T.S., Whitefield S., Weissman T., Wasserzug O., Yahalom R. (2018). Rapid onset of osteonecrosis of the jaw in patients switching from bisphosphonates to denosumab. Oral Surg. Oral Med. Oral Pathol. Oral Radiol..

[135] Ciobanu G.A., Mogoantă L., Popescu S.M., Ionescu M., Munteanu C.M., Staicu I.E., Mercuț R., Georgescu C.C., Scrieciu M., Vlad D. (2023). Correlations between Immune Response and Etiopathogenic Factors of Medication-Related Osteonecrosis of the Jaw in Cancer Patients Treated with Zoledronic Acid. Int. J. Mol. Sci..

[136] Taniguchi N., Osaki M., Onuma K., Ishikawa M., Ryoke K., Kodani I., Okada F. (2020). Bisphosphonate-induced reactive oxygen species inhibit proliferation and migration of oral fibroblasts: A pathogenesis of bisphosphonate-related osteonecrosis of the jaw. J. Periodontol..

[137] Ciobanu G.A., Camen A., Ionescu M., Vlad D., Mercut V., Staicu I.E., Petrescu G.S., Asan A.A., Popescu S.M. (2022). Bisphosphonates related osteonecrosis of the jaw in cancer patients—Epidemiological study. Rom. J. Oral Rehabil..

[138] Ciobanu G.A., Mogoantă L., Camen A., Ionescu M., Vlad D., Staicu I.E., Munteanu C.M., Gheorghit M.I., Mercut R., Sin E.C. (2023). Clinical and Histopathological Aspects of MRONJ in Cancer Patients. J. Clin. Med..

[139] Kim H.Y. (2021). Review and Update of the Risk Factors and Prevention of Antiresorptive-Related Osteonecrosis of the Jaw. Endocrinol. Metab..

[140] Querrer R., Ferrare N., Melo N., Stefani C.M., Dos Reis P.E.D., Mesquita C.R.M., Borges G.A. (2021). Differences between bisphosphonate-related and denosumab-related osteonecrosis of the jaws: A systematic review. Support. Care Cancer.

[141] Di Fede O., Canepa F., Panzarella V., Mauceri R., Del Gaizo C., Bedogni A., Fusco V., Tozzo P., Pizzo G., Campisi G. (2021). The Treatment of Medication-Related Osteonecrosis of the Jaw (MRONJ): A Systematic Review with a Pooled Analysis of Only Surgery versus Combined Protocols. Int. J. Environ. Res. Public Health.

[142] Moraschini V., Calasans-Maia M.D., Louro R.S., Arantes E.B.R., Calasans-Maia J.A. (2021). Weak evidence for the management of medication-related osteonecrosis of the jaw: An overview of systematic reviews and meta-analyses. J. Oral Pathol. Med..

[143] Campisi G., Bedogni A., Fusco V. Raccomandazioni Clinico-Terapeutiche sull’Osteonecrosi delle ossa Mascellari (ONJ) Farmaco-Relata e sua Prevenzione. Version 2.0-July 2020. https://www.sipmo.it/versione-2-0-delle-raccomandazioni-clinico-terapeutiche-sullosteonecrosi-delle-ossa-mascellari-onj-farmaco-relata-e-sua-prevenzione/.

[144] Aguirre J.I., Castillo E.J., Kimmel D.B. (2021). Biologic and pathologic aspects of osteocytes in the setting of medication-related osteonecrosis of the jaw (MRONJ). Bone.

[145] De Cicco D., Boschetti C.E., Santagata M., Colella G., Staglianò S., Gaggl A., Bottini G.B., Vitagliano R., D’amato S. (2023). Medication-Related Osteonecrosis of the Jaws: A Comparison of SICMF–SIPMO and AAOMS Guidelines. Diagnostics.

[146] Marx R.E. (2003). Pamidronate (Aredia) and zoledronate (Zometa) induced avascular necrosis of the jaws: A growing epidemic. J. Oral Maxillofac. Surg..

[147] Moraschini V., de Almeida D.C.F., Figueredo C.M., Calasans-Maia M.D. (2019). Association between biomarkers and medication-related osteonecrosis of the jaws: A systematic review. Oral Surg. Oral Med. Oral Pathol. Oral Radiol..

[148] Lorenzo-Pouso A.I., Pérez-Sayáns M., González-Palanca S., Chamorro-Petronacci C., Bagán J., García-García A. (2019). Biomarkers to predict the onset of biphosphonate-related osteonecrosis of the jaw: A systematic review. Med. Oral Patol. Oral Cir. Bucal..

[149] Prá K.D., Lemos C., Okamoto R., Soubhia A., Pellizzer E. (2017). Efficacy of the C-terminal telopeptide test in predicting the development of bisphosphonate-related osteonecrosis of the jaw: A systematic review. Int. J. Oral Maxillofac. Surg..

[150] Tsuchimochi M., Kurabayashi T. (2019). Symposium: Imaging modalities for drug-related osteonecrosis of the jaw (1), role of imaging in drug-related osteonecrosis of the jaw: An up-to-date review (secondary publication). Jpn. Dent. Sci. Rev..

[151] Melguizo-Rodríguez L., Costela-Ruiz V.J., Manzano-Moreno F.J., Ruiz C., Illescas-Montes R. (2020). Salivary Biomarkers and Their Application in the Diagnosis and Monitoring of the Most Common Oral Pathologies. Int. J. Mol. Sci..

[152] Bagan J., Sheth C.C., Soria J.M., Margaix M., Bagan L. (2012). Bisphosphonates-related osteonecrosis of the jaws: A preliminary study of salivary interleukins. J. Oral Pathol. Med..

[153] Bagan J., Sáez G., Tormos M., Hens E., Terol M., Bagan L., Diaz-Fernandez J., Lluch A., Camps C. (2013). Interleukin-6 concentration changes in plasma and saliva in bisphosphonate-related osteonecrosis of the jaws. Oral Dis..

[154] Thumbigere-Math V., Michalowicz B.S., De Jong E.P., Griffin T.J., Basi D.L., Hughes P.J., Tsai M.L., Swenson K.K., Rockwell L., Gopalakrishnan R. (2013). Salivary proteomics in bisphosphonate-related osteonecrosis of the jaw. Oral Dis..

[155] Thumbigere-Math V., Michalowicz B.S., Hughes P.J., Basi D.L., Tsai M.L., Swenson K.K., Rockwell L., Gopalakrishnan R. (2015). Serum Markers of Bone Turnover and Angiogenesis in Patients with Bisphosphonate-Related Osteonecrosis of the Jaw after Discontinuation of Long-Term Intravenous Bisphosphonate Therapy. J. Oral Maxillofac. Surg..

[156] Kim J.W., Kwak M.K., Han J.J., Lee S.T., Kim H.Y., Kim S.H., Jung J., Lee J.K., Lee Y.K., Kwon Y.D. (2021). Medication Related Osteonecrosis of the Jaw: 2021 Position Statement of the Korean Society for Bone and Mineral Research and the Korean Association of Oral and Maxillofacial Surgeons. J. Bone Metab..

[157] Hanley D.A., Adachi J.D., Bell A., Brown V. (2012). Denosumab: Mechanism of action and clinical outcomes. Int. J. Clin. Pract..

[158] Hansen T., Kunkel M., Weber A., James Kirkpatrick C. (2006). Osteonecrosis of the jaws in patients treated with bisphosphonates-histomorphologic analysis in comparison with infected osteoradionecrosis. J. Oral Pathol. Med..

[159] Koizumi G., Hayashi A., Takigawa A., Yamada R., Murata T., Shimizu K., Watanabe M., Arai N. (2024). Novel Histopathological Findings of Micro Bone Fragments and Epithelial Response in the Oral Mucosa in Bisphosphonate-Related Osteonecrosis of the Jaw. J. Investig. Med. High Impact Case Rep..

[160] Sedghizadeh P.P., Yooseph S., Fadrosh D.W., Zeigler-Allen L., Thiagarajan M., Salek H., Farahnik F., Williamson S.J. (2012). Metagenomic investigation of microbes and viruses in patients with jaw osteonecrosis associated with bisphosphonate therapy. Oral Surg. Oral Med. Oral Pathol. Oral Radiol..

[161] Pushalkar S., Li X., Kurago Z., Ramanathapuram L.V., Matsumura S., Fleisher K.E., Glickman R., Yan W., Li Y., Saxena D. (2014). Oral microbiota and host innate immune response in bisphosphonate-related osteonecrosis of the jaw. Int. J. Oral Sci..

[162] Govaerts D., Piccart F., Ockerman A., Coropciuc R., Politis C., Jacobs R. (2020). Adjuvant therapies for MRONJ: A systematic review. Bone.

[163] Kuroshima S., Sasaki M., Sawase T. (2019). Medication-related osteonecrosis of the jaw: A literature review. J. Oral Biosci..

[164] Ottesen C., Schiodt M., Gotfredsen K. (2020). Efficacy of a high-dose antiresorptive drug holiday to reduce the risk of medication-related osteonecrosis of the jaw (MRONJ): A systematic review. Heliyon.

[165] Khan L.A., Morrison A., Hanley D.A., Felsenberg D., McCauley L.K., O’Ryan F., Reid I.R., Ruggiero S.L., Taguchi A., Tetradis S. (2015). on behalf of the International Task Force on Osteonecrosis of the Jaw, Diagnosis and Management of Osteonecrosis of the Jaw: A Systematic Review and International Consensus. J. Bone Min. Res..

[166] Wilde F., Heufelder M., Winter K., Hendricks J., Frerich B., Schramm A., Hemprich A. (2011). The role of surgical therapy in the management of intravenous bisphosphonates-related osteonecrosis of the jaw. Oral Surg. Oral Med. Oral Pathol. Oral Radiol. Endod..

[167] Bermúdez-Bejarano E.-B., Serrera-Figallo M.Á., Gutiérrez-Corrales A., Romero-Ruiz M.-M., Castillo-De-Oyagüe R., Gutiérrez-Pérez J.-L., Torres-Lagares D. (2017). Prophylaxis and antibiotic therapy in management protocols of patients treated with oral and intravenous bisphosphonates. J. Clin. Exp. Dent..

[168] De Bruyn L., Coropciuc R., Coucke W., Politis C. (2018). Microbial population changes in patients with medication-related osteonecrosis of the jaw treated with systemic antibiotics. Oral Surg. Oral Med. Oral Pathol. Oral Radiol..

[169] Boff R.C., Salum F.G., Figueiredo M.A., Cherubini K. (2014). Important aspects regarding the role of microorganisms in bisphosphonate-related osteonecrosis of the jaws. Arch. Oral Biol..

[170] Shamsoddin E., Mahboobi F., Kargar K., Latifi F. (2019). Etiologic Role of Bacterial Microorganisms in Medication Related Osteonecrosis of the Jaws: A Systematic Review. Int. J. Pharm. Phytopharm..

[171] Kiss C., Connoley D., Connelly K., Horne K., Korman T., Woolley I., Lau J.S.Y. (2022). Long-Term Outcomes in Patients on Life-Long Antibiotics: A Five-Year Cohort Study. Antibiotics.

[172] Walker G.T., Quan J., Higgins S.G., Toraskar N., Chang W., Saeed A., Sapiro V., Pitzer K., Whitfield N., Lopansri B.K. (2019). Predicting antibiotic resistance in gram-negative bacilli from resistance genes. Antimicrob. Agents Chemother..

[173] Ji X., Pushalkar S., Li Y., Glickman R., Fleisher K., Saxena D. (2012). Antibiotic effects on bacterial profile in osteonecrosis of the jaw. Oral Dis..

[174] Ewald F., Wuesthoff F., Koehnke R., Friedrich R.E., Gosau M., Smeets R., Rohde H., Assaf A.T. (2021). Retrospective analysis of bacterial colonization of necrotic bone and antibiotic resistance in 98 patients with medication-related osteonecrosis of the jaw (MRONJ). Clin. Oral Investig..

[175] Sawada K., Fujioka-Kobayashi M., Kobayashi E., Schaller B., Miron R.J. (2016). Effects of Antiseptic Solutions Commonly Used in Dentistry on Bone Viability, Bone Morphology, and Release of Growth Factors. J. Oral Maxillofac. Surg..

[176] Fortunato L., Bennardo F., Buffone C., Giudice A. (2020). Is the application of platelet concentrates effective in the prevention and treatment of medication-related osteonecrosis of the jaw? A systematic review. J. Craniomaxillofacial Surg..

[177] Del Fabbro M., Gallesio G., Mozzati M. (2015). Autologous platelet concentrates for bisphosphonate-related osteonecrosis of the jaw treatment and prevention. A systematic review of the literature. Eur. J. Cancer.

[178] Scribante A., Ghizzoni M., Pellegrini M., Pulicari F., Spadari F. (2023). Laser Devices and Autologous Platelet Concentrates in Prevention and Treatment of Medication-Related Osteonecrosis of the Jaws: A Systematic Review. Medicina.

[179] Mücke T., Haarmann S., Wolff K.D., Hölzle F. (2009). Bisphosphonate related osteonecrosis of the jaws treated by surgical resection and immediate osseous microvascular reconstruction. J. Craniomaxillofacial Surg..

[180] Ristow O., Otto S., Troeltzsch M., Hohlweg-Majert B., Pautke C. (2015). Treatment perspectives for medication-related osteonecrosis of the jaw (MRONJ). J. Craniomaxillofacial Surg..

[181] Giudice A., Bennardo F., Barone S., Antonelli A., Figliuzzi M.M., Fortunato L. (2018). Can Autofluorescence Guide Surgeons in the Treatment of Medication-Related Osteonecrosis of the Jaw? A Prospective Feasibility Study. J. Oral Maxillofac. Surg..

[182] de Mello E.D., Pagnoncelli R.M., Munin E., Filho M.S., de Mello G.P., Arisawa E.A., de Oliveira M.G. (2008). Comparative histological analysis of bone healing of standardized bone defects performed with the Er:YAG laser and steel burs. Lasers Med. Sci..

[183] de Souza Tolentino E., de Castro T.F., Michellon F.C., Passoni A.C.C., Ortega L.J.A., Iwaki L.C.V., da Silva M.C. (2019). Adjuvant therapies in the management of medication-related osteonecrosis of the jaws: Systematic review. Head Neck.

[184] Holzinger D., Seemann R., Klug C., Ewers R., Millesi G., Baumann A., Wutzl A. (2013). Long-term success of surgery in bisphosphonate-related osteonecrosis of the jaws (BRONJs). Oral Oncol..

[185] Fusco V., Campisi G., Carcieri P., Fagioli F., Bertetto O., Mignogna M.D., Bedogni A. (2022). *ONJ (MRONJ) Update 2021*—Osteonecrosis of Jaw Related to Bisphosphonates and Other Drugs—Prevention, Diagnosis, Pharmacovigilance, Treatment: A 2021 Web Event. Oral.

[186] Katsarelis H., Shah N.P., Dhariwal D.K., Pazianas M. (2015). Infection and medication-related osteonecrosis of the jaw. J. Dent. Res..

[187] Ruggiero S.L. (2011). Bisphosphonate-related osteonecrosis of the jaw: An overview. Ann. N. Y. Acad. Sci..

[188] Lopez-Jornet P., Sanchez Perez A., Amaral Mendes R., Tobias A. (2016). Medication-Related Osteonecrosis of the Jaw: Is Autologous Platelet Concentrate Application Effective for Prevention and Treatment? A Systematic Review. J. Craniomaxillofacial Surg..

[189] Porcaro G., Caccianiga P., Bader A.A., Caccianiga G. (2022). Treatment of Medication-Related Osteonecrosis of the Jaw (MRONJ) with Er:YaG Laser and Ozone Therapy: A Case Series. Inventions.

[190] Lee L.W., Hsiao S.H., Chen L.K. (2014). Clinical treatment outcomes for 40 patients with bisphosphonates-related osteonecrosis of the jaws. J. Formos. Med. Assoc..

[191] Khan I., Rahman S.U., Tang E., Engel K., Hall B., Kulkarni A.B., Arany P.R. (2021). Accelerated burn wound healing with photobiomodulation therapy involves activation of endogenous latent TGF-β1. Sci. Rep..

[192] de Barros Silva P.G., de Lima Praxedes Praxedes Neto R.A., Lima L.A., Lemos J.V.M., De Queiroz Rodrigues M.I., Alves A.P.N.N., Dantas T.S., Lima R.A. (2022). Photodynamic therapy and photobiomodulation therapy in zoledronic acid-induced osteonecrosis in rats. Photodiagnosis Photodyn. Ther..

[193] Wehrhan F., Hyckel P., Guentsch A., Nkenke E., Stockmann P., Schlegel K.A., Neukam F.W., Amann K. (2011). Bisphosphonate-associated osteonecrosis of the jaw is linked to suppressed TGFβ1-signaling and increased Galectin-3 expression: A histological study on biopsies. J. Transl. Med..

[194] Kim S., Williams D.W., Lee C., Kim T., Arai A., Shi S., Li X., Shin K.-H., Kang M.K., Park N.-H. (2017). IL-36 induces bisphosphonate-related osteonecrosis of the jaw-like lesions in mice by inhibiting TGF-β-mediated collagen expression. J. Bone Min. Res..

[195] Lee K.H., Kim S.H., Kim C.H., Min B.J., Kim G.J., Lim Y., Kim H.-S., Ahn K.-M., Kim J.H. (2019). Identifying genetic variants underlying medication-induced osteonecrosis of the jaw in cancer and osteoporosis: A case control study. J. Transl. Med..

[196] Robijns J., Nair R.G., Lodewijckx J., Arany P., Barasch A., Bjordal J.M., Bossi P., Chilles A., Corby P.M., Epstein J.B. (2022). Photobiomodulation therapy in management of cancer therapy-induced side effects: WALT position paper 2022. Front. Oncol..

[197] Sim I.W., Sanders K.M., Borromeo G.L., Seymour J.F., Ebeling P.R. (2015). Declining incidence of medication-related osteonecrosis of the jaw in patients with cancer. J. Clin. Endocrinol. Metab..

[198] Nicolatou-Galitis O., Schiødt M., Mendes R.A., Ripamonti C., Hope S., Drudge-Coates L., Niepel D., Van den Wyngaert T. (2019). Medication-related osteonecrosis of the jaw: Definition and best practice for prevention, diagnosis, and treatment. Oral Surg. Oral Med. Oral Pathol. Oral Radiol..

[199] Matsumoto A., Sasaki M., Schmelzeisen R., Oyama Y., Mori Y., Voss P.J. (2017). Primary wound closure after tooth extraction for prevention of medication-related osteonecrosis of the jaw in patients under denosumab. Clin. Oral Investig..

[200] Hasegawa T., Kawakita A., Ueda N., Funahara R., Tachibana A., Kobayashi M., Kondou E., Takeda D., Kojima Y., Sato S. (2017). A multicenter retrospective study of the risk factors associated with medication-related osteonecrosis of the jaw after tooth extraction in patients receiving oral bisphosphonate therapy: Can primary wound closure and a drug holiday really prevent MRONJ?. Osteoporos. Int..

[201] Beth-Tasdogan N.H., Mayer B., Hussein H., Zolk O., Peter J.U. (2022). Interventions for managing medication-related osteonecrosis of the jaw. Cochrane Database Syst. Rev..

[202] Mosaico G., Casu C. (2024). Management and maintenance of oral health: Personalized primary prevention strategies and protocols in patients at risk of developing medication-related osteonecrosis of the jaw. INNOSC Pharmacol. Sci..

[203] Rupel K., Ottaviani G., Gobbo M., Contardo L., Tirelli G., Vescovi P., Di Lenarda R., Biasotto M. (2014). A systematic review of therapeutical approaches in bisphosphonates-related osteonecrosis of the jaw (BRONJ). Oral Oncol..

[204] Weber J.B., Camilotti R.S., Ponte M.E. (2016). Efficacy of laser therapy in the management of bisphosphonate-related osteonecrosis of the jaw (BRONJ): A systematic review. Lasers Med. Sci..

[205] Li F.L., Wu C.B., Sun H.J., Zhou Q. (2020). Effectiveness of laser-assisted treatments for medication-related osteonecrosis of the jaw: A systematic review. Br. J. Oral Maxillofac. Surg..

[206] Razavi P., Jafari A., Vescovi P., Fekrazad R. (2022). Efficacy of Adjunctive Photobiomodulation in the Management of Medication-Related Osteonecrosis of the Jaw: A Systematic Review. Photobiomodulation Photomed. Laser Surg..

[207] Somerfield M.R., Padberg J.R., Pfister D.G., Bennett C.L., Recht A., Smith T.J., Weeks R.J., Durant J.R. (2000). ASCO clinical practice guidelines: Process, progress, pitfalls, and prospects. Class. Pap. Curr. Comments.

[208] Kaptein M. (2019). A practical approach to sample size calculation for fixed populations. Contemp. Clin. Trials Commun..

[209] Das Graças Miranda Coutinho B., Gerônimo Caetano A.B., Oliveira A.L., De Castro Lara S.M., Wilker Mustafa Gomes Muniz F., Barbosa Calcia T.B. (2022). clinical effectiveness of photodynamic antimicrobial therapy in mronj—Systematic review. Oral Surg. Oral Med. Oral Pathol. Oral Radiol..

[210] Zheng Y., Dong X., Chen S., He Y., An J., Liu M., He L., Zhang Y. (2023). Low-level laser therapy prevents medication-related osteonecrosis of the jaw-like lesions via IL-1RA-mediated primary gingival wound healing. BMC Oral Health.

